# How animals distribute themselves in space: energy landscapes of Antarctic avian predators

**DOI:** 10.1186/s40462-021-00255-9

**Published:** 2021-05-17

**Authors:** Juan F. Masello, Andres Barbosa, Akiko Kato, Thomas Mattern, Renata Medeiros, Jennifer E. Stockdale, Marc N. Kümmel, Paco Bustamante, Josabel Belliure, Jesús Benzal, Roger Colominas-Ciuró, Javier Menéndez-Blázquez, Sven Griep, Alexander Goesmann, William O. C. Symondson, Petra Quillfeldt

**Affiliations:** 1 Department of Animal Ecology & Systematics, Justus Liebig University Giessen, Heinrich-Buff-Ring 26, D-35392 Giessen, Germany; 2 Department Ecología Evolutiva, Museo Nacional de Ciencias Naturales, CSIC, C/José Gutiérrez Abascal, 2, 28006 Madrid, Spain; 3 Centre d’Etudes Biologiques de Chizé, UMR7372 CNRS-Université La Rochelle, 79360 Villiers en Bois, France; 4 New Zealand Penguin Initiative, PO Box 6319, Dunedin, 9022 New Zealand; 5 Cardiff School of Biosciences, Cardiff University, The Sir Martin Evans Building, Museum Av, Cardiff, CF10 3AX UK; 6 Cardiff School of Dentistry, Heath Park, Cardiff, CF14 4XY UK; 7 Institute for Bioinformatics & Systems Biology, Justus Liebig University Giessen, Heinrich-Buff-Ring 58, D-35392 Giessen, Germany; 8 Littoral Environnement et Sociétés (LIENSs), UMR 7266 CNRS-Université de La Rochelle, 17000 La Rochelle, France; 9 Institut Universitaire de France (IUF), 1 rue Descartes, 75005 Paris, France; 10 GLOCEE - Global Change Ecology and Evolution Group, Universidad de Alcalá, Madrid, Spain; 11 Estación Experimental de Zonas Áridas, CSIC, Almería, Spain

**Keywords:** Antarctica, Breeding success, Chinstrap penguin *Pygoscelis antarcticus*, Energy costs, Energy landscapes, Gentoo penguin *Pygoscelis papua*, Physiological condition, Physiological stress, Population trends, Sub-Antarctic

## Abstract

**Background:**

Energy landscapes provide an approach to the mechanistic basis of spatial ecology and decision-making in animals. This is based on the quantification of the variation in the energy costs of movements through a given environment, as well as how these costs vary in time and for different animal populations. Organisms as diverse as fish, mammals, and birds will move in areas of the energy landscape that result in minimised costs and maximised energy gain. Recently, energy landscapes have been used to link energy gain and variable energy costs of foraging to breeding success, revealing their potential use for understanding demographic changes.

**Methods:**

Using GPS-temperature-depth and tri-axial accelerometer loggers, stable isotope and molecular analyses of the diet, and leucocyte counts, we studied the response of gentoo (*Pygoscelis papua*) and chinstrap (*Pygoscelis antarcticus*) penguins to different energy landscapes and resources. We compared species and gentoo penguin populations with contrasting population trends.

**Results:**

Between populations, gentoo penguins from Livingston Island (Antarctica), a site with positive population trends, foraged in energy landscape sectors that implied lower foraging costs per energy gained compared with those around New Island (Falkland/Malvinas Islands; sub-Antarctic), a breeding site with fluctuating energy costs of foraging, breeding success and populations. Between species, chinstrap penguins foraged in sectors of the energy landscape with lower foraging costs per bottom time, a proxy for energy gain. They also showed lower physiological stress, as revealed by leucocyte counts, and higher breeding success than gentoo penguins. In terms of diet, we found a flexible foraging ecology in gentoo penguins but a narrow foraging niche for chinstraps.

**Conclusions:**

The lower foraging costs incurred by the gentoo penguins from Livingston, may favour a higher breeding success that would explain the species’ positive population trend in the Antarctic Peninsula. The lower foraging costs in chinstrap penguins may also explain their higher breeding success, compared to gentoos from Antarctica but not their negative population trend. Altogether, our results suggest a link between energy landscapes and breeding success mediated by the physiological condition.

**Supplementary Information:**

The online version contains supplementary material available at 10.1186/s40462-021-00255-9.

## Background

The current degree of anthropogenic space use, both at sea and land, and climate change make it imperative to understand animal movement, if meaningful conservation and management measures are to be taken [1–3]. Animals move to find critical resources [4] but increasingly, they have to negotiate habitats that are intensively-used, fragmented, impoverished, or modified by climate change, which may determine individual survival and thus, population dynamics and persistence [5–7]. Simultaneously, a growing availability of high-resolution animal tracking technologies has greatly enhanced our ability to describe animal movements [4, 8, 9], which in turns guides and refines conservation and management measures [10, 11]. Moreover, current technologies offer a unique opportunity to explore pioneering questions in ecology, and to explain in depth the causes and fundamental mechanisms of movement patterns and their significance for ecological and evolutionary processes [8, 9, 12].

The first systematic attempts to understand the role of behaviour in the distribution of animals originated from optimal foraging theory [13, 14]. In this context, animals should exhibit behaviours that maximize energetic efficiency, selecting patches where the gain per unit cost is high, and the energy expenditure to reach them is minimized. As movement accounts for such a large proportion of animal energy budgets, energetic constraints with respect to space use, migration and foraging range are foreseeable factors [15–17]. Unnecessary movements and resulting energy deficits might increase the risk of predation, reduce body condition, increase physiological stress, affect fitness, and since the sum of individual responses is ultimately reflected at the population-level, be the cause of population declines [12, 18–22]. Animal movement has also been investigated in terms of the physical mechanics of motion (biomechanical paradigm), the movement-related decisions made by the individuals (cognitive paradigm), and the theories of random walk, diffusion, and anomalous diffusion (random paradigm) [6]. More recently, the paradigm of energy landscape has opened a new approach to the mechanistic basis of spatial ecology and decision-making in wild animals [12]. The energy landscape paradigm (sensu Wilson et al.) [23] allows the quantification of the variation in the energy costs of the movement through a given environment [12], as well as how these costs vary in time and for different animal populations moving there [21], using for instance environmentally dependent costs of transport generated by parameters such as incline, substrate type, vegetation, current speed, or direction [24]. Research conducted in organisms as diverse as fish, mammals, and birds showed that animals will move in areas of the energy landscape that result in minimized costs and maximised energy gain [19, 21, 23, 25–27].

In seabirds, variable oceanographic conditions and fluctuating food availability can affect the costs of moving and energy landscapes capture this variation successfully [21]. For instance, considering the energetic costs and duration of flights, dive and inter-dive phases, Wilson et al. [23] found that imperial cormorants *Phalacrocorax atriceps* selected foraging areas that varied greatly in the distance from the breeding colony and in water depth, but always indicated minimal energetic cost of movement compared with other areas in the available landscape. Likewise, evaluating the daily energy requirements of an individual using the biophysical properties of bodies (body shape and its heat flux) exposed to specific microclimatic conditions (sea surface temperature, SST, air temperature, cloud cover, relative humidity and wind speed), Amélineau et al. [27] found that little auks *Alle alle* targeted areas with moderately elevated energy landscapes in winter. In gentoo penguins *Pygoscelis papua* (hereafter gentoos), when considering mass-specific costs of foraging to dive to a particular depth plus commuting to a certain distance, and energy gained in terms of diving bottom time, the energy landscapes around two nearby colonies varied strongly between years. Yet, the birds consistently used the areas of the energy landscape that resulted in lower foraging costs. However, for these gentoos the breeding success was low in a year of higher energy expenditure, while it was high during a year of lower energy expenditure, suggesting the usefulness of energy landscapes to understand demographic changes and their consequences for conservation [21].

We combined information from previous work on the energy landscape in gentoos [21] with novel data on movement and diet and 1) studied the response of moving animals to different energy landscapes and resources, and 2) compared populations with contrasting population trends. Gentoos are facing strong environmental change both in Antarctic and sub-Antarctic regions. The Antarctic Peninsula is one of the places where current environmental change is fastest [28]. In both regions, gentoos are known to show considerable plasticity in their diet, diving, and foraging behaviour [29, 30], providing a buffer against changes in prey availability [31]. However, gentoos exhibit strikingly different population trends in sub-Antarctic and Antarctic populations. Since 1990, gentoos at the Falkland/Malvinas Islands showed a great degree of inter-annual variability in the number of breeding individuals, which has been related to the Southern Oscillation Index (SOI) and the El Niño Southern Oscillation (ENSO), yet the underlying mechanisms remain unknown [32]. In contrast, gentoos have been increasing at breeding colonies along the Antarctic Peninsula and expanded southwards since 1979 [33–35]. This positive population trend was understood as gentoos being the ‘winners’ among *Pygoscelis* penguins of the reduction in the sea-ice cover in the region because it positively affects its winter survival (sea-ice hypothesis) [36]. An alternative hypothesis postulated that penguin population dynamics in Antarctica were instead controlled through “top-down” factors such as competition for prey [37], while another related hypothesis suggested a link between penguin population trends and changes in the abundance of their main prey, Antarctic krill *Euphausia superba* [38]. However, it has been shown that sea-ice cover and krill abundance are interrelated [39, 40]. Even more, other aspects need to be considered, such as fine-scale spatial heterogeneity in population dynamics observed on the Antarctic Peninsula [41], intra-specific competition [40], and adaptive shifts in trophic position [42]. But, regardless of this research, no study has yet considered the cost of foraging. The energy landscape approach could provide a way to better understand the ecological processes involved, as the energetic balance between costs and benefits will affect how and which foraging areas are selected or avoided, and the condition of the birds which in turn will affect reproductive success and ultimately population dynamics.

In our present study we tested the following hypotheses: a) in optimal sites (Antarctic Peninsula and islands around it) gentoos forage in sectors of the energy landscapes where low energy is required, b) in suboptimal breeding sites like the Falkland/Malvinas Islands (fluctuating populations) gentoos are forced to forage in more expensive conditions in the poorer years, and c) foraging in areas of the energy landscapes that result in minimized energetic costs will lead to better individual condition, as shown by physiological parameters such as leucocyte counts. To understand our results in a wider context, we also investigated the diet and the energy landscape in chinstrap penguins *Pygoscelis antarcticus* (hereafter chinstraps), an Antarctic species with currently declining populations [35, 43, 44]. We tested the hypothesis that d) chinstraps show higher energy expenditure than Antarctic gentoos.

## Methods

### Study sites and species

**Fig. 1:**
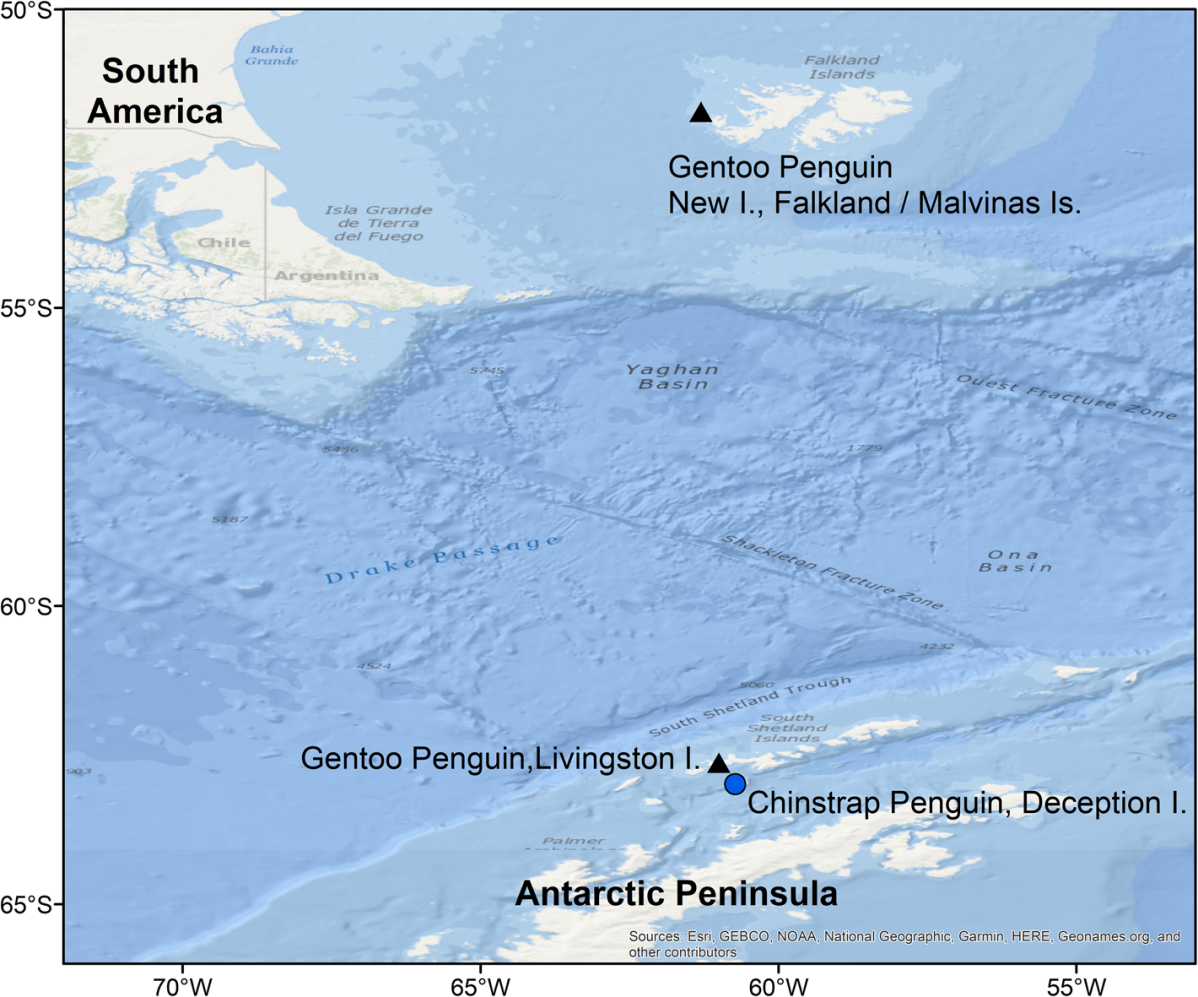
Overview of the location of the studied gentoo penguin *Pygoscelis papua* colonies at Devils Point, Byers Peninsula, Livingston Island, South Shetland Islands, maritime Antarctica, and New Island, Falkland/Malvinas Islands, and the chinstrap penguin *Pygoscelis antarcticus* colony at Vapour Col rookery, Deception Island, South Shetland Islands, maritime Antarctica

We collected data on three penguin populations: gentoos from an Antarctic and a sub-Antarctic breeding site, and chinstraps from an Antarctic breeding site. We studied a population of gentoos breeding at a colony located in Devils Point, Byers Peninsula, Livingston Island, South Shetland Islands, maritime Antarctica (hereafter Livingston; 3000 nests; 62°40′S, 61°13′W; Fig. 1) [45]. Byers is characterised by a high biological diversity due to relatively mild climatic conditions and a large ice-free area in summer [45]. This breeding population is located in an optimal breeding site, as gentoos are increasing in numbers in this location in the last decades [45], following the population increase and area expansion in this region [33, 41]. We furthermore investigated energy landscapes of chinstraps at Vapour Col rookery on the west side of Deception Island, South Shetland Islands (hereafter Deception; 20,000 breeding pairs; 63° 00′S, 62°40′W; Fig. 1) [43], a species declining on the Antarctic Peninsula [41, 44]. We further studied the foraging strategies and mechanism of gentoos of a fluctuating population, New Island in the Falkland/Malvinas Islands (hereafter New Island) [21, 32]. On New Island, we investigated two breeding colonies: one located at the North End (around 5000 breeding pairs; 51° 41.402′ S 61° 15.003′ W), and one at the South End (around 2000 breeding pairs 51° 44.677′ S 61°17.683′ W) [46]. The data previously obtained at New Island [21], as well as samples analysed in current study, are used for the comparisons between optimal and suboptimal breeding sites.

### Instrumentation and fieldwork procedures

We simultaneously deployed a combination of GPS-temperature-depth (GPS-TD; earth&OCEAN Technologies, Kiel) and micro tri-axial accelerometer loggers (Axy-2; Technosmart Europe, Rome, Italy) or Axy-Trek loggers only (including GPS, accelerometer, and both pressure and temperature sensors), on the penguins during chick guard. For sample sizes per study site and species see Table 1. We captured the birds mostly by hand, in the nests, with the occasional help of a hook attached to a rod [21] or a long-handle net [47]. To protect them from predators, we also captured the chicks during the handling of the adult. We kept handling time mostly below 15 min and always below 20 min. We took extreme care to minimize stress to the captured birds, covering the head during handling in order to minimize the risk of adults regurgitating. During this procedure none of the birds regurgitated. We attached the loggers on the adult penguin with adhesive Tesa® 4651 tape [21]. The loggers used (GPS-TD: 75 to 145 g and Axy-2: 19 g; Axy-Trek: 60 g) represented a maximum of a 3% of the adult gentoo body mass (mean for Livingston 5212.8 ± 478.2 g, *n* = 25) or 4% of the adult chinstrap body mass (mean for Deception 3743.5 ± 425.4 g, *n* = 20), and had a shape that matched the body contour to reduce drag [48]. In a previous study [49], we showed that handling and short-term logger attachments like the ones in this study showed limited effect on the behaviour and physiology of the birds. After the deployment procedure and immediately before the release of the adult bird, we returned the chicks to the nest, and released the adults some 20 m from their nests. All birds returned to their nests and attended their chicks shortly after being released. The loggers recorded detailed position (longitude, latitude; sampling interval: 5 min), dive depth (resolution: 3.5 cm; sampling interval: 1 s), time of day and acceleration (sampling interval: 50 Hz) measured in three directions (x, y, z, i.e. surge, sway, heave) [21]. The devices operated for three to 9 days and had to be recovered to access recorded data. We recaptured the birds in their nests. After device removal, we measured flipper and bill length, bill depth, and body mass, and collected blood samples (200 μl) from the foot (Antarctica) or the brachial (New Island) vein, and four small feathers from the lower back of the adults. Blood and feather samples were used for the study of stable isotopes (see *Stable isotope analysis of the diet* below) and molecular sexing (following standard methods) [50]. As in previous studies [21, 51], we detected no adverse effects related to blood sampling. One drop of blood was smeared and air dried on a glass slide directly after sampling, and fixed with absolute methanol and stained with Giemsa dye later in the laboratory [52]. Blood smears were used for differential leucocyte counts (see *Condition parameters* below). Additionally, we collected fresh scat samples opportunistically during the handling of the birds, as well as from randomly located ice or rock substrates around the penguin colonies, immediately after defecation. To avoid external contamination, we took special care to collect the central part of the scat and not the part that was in direct contact with the substrates. We kept scat samples cool with ice packs during fieldwork, froze them once back at the field station, and transported frozen until processed in the laboratory.

**Table 1:** Parameters of foraging trips used for the calculations of energy landscapes

	Gentoo					Chinstrap
	Dec 2013	Dec 2014		Dec 2016		Jan 2017
	New I., South	New I., South	New I., North	Livingston I.		Deception I.
				Short trips	Long trips	
Individuals tagged	16	8	8	26		18
Number of complete trips	13	4	6	26	19	18
Median trip length [km]	125.6 ^b^ (87.4–161.8)	88.7 ^a, b^ (40.8–144.7)	59.1 ^a^ (52.2–61.7)	27.1 ^c^ (19.9–33.4)	66.6 ^a^ (59.2–71.0)	37.7 ^d^ (21.7–49.5)
Kruskal-Wallis	χ^2^ = 72.1, df = 5, ***P*** **< 0.001**					
Median maximum distance from colony [km]	66.9 ^b^ (63.2–75.6)	47.7 ^a, b^ (23.7–75.6)	29.6 ^a, b^ (19.8–45.1)	11 ^c^ (8.6–13.4)	25.7 ^a^ (23.5–32.1)	15.5 ^d^ (8.7–20.2)
Kruskal-Wallis	χ^2^ = 75.3, df = 5, ***P*** **< 0.001**					
Median trip duration [min]	1727.3 ^a^ (1062.4–2432.6)	1579.6 ^a^ (765.2–2508.0)	1129 ^a^ (850.3–1538.9)	503.4 ^b^ (373.2–641.7)	1049 ^a^ (866.1–1182)	595.5 ^b^ (371.2–641.6)
Kruskal-Wallis	χ^2^ = 67, df = 5, ***P*** **< 0.001**					
Median start time of foraging (local time)	03:41:46 ^c^ (03:05:46–14:18:14)	16:49:26 ^a, b^ (11:47:02–18:25:55)	09:15:50 ^a, b, c^ (03:14:24–17:13:55)	14:52:48 ^a^ (10:10:34–17:45:36)	09:31:41 ^b, c^ (03:10:05–16:00:29)	15:38:53 ^a^ (07:16:19–18:34:34)
Kruskal-Wallis	χ^2^ = 17.3 df = 5, ***P*** **< 0.001**					

The data correspond to gentoo penguins *Pygoscelis papua* breeding at New Island (Falkland/Malvinas Islands), during chick guard (December) in 2013 and 2014, gentoo penguins breeding at Devils Point, Byers Peninsula, Livingston Island, South Shetland Islands, Antarctica, during chick guard (December 2016), and chinstrap penguins *Pygoscelis antarcticus* breeding at Vapour Col rookery, Deception Island, South Shetland Islands, Antarctica, during chick guard (January 2017). See also Figs. S7 and S8 in Additional file 1

*Note*: Sample sizes vary with respect to deployments, as not all parameters could be calculated for all individuals, mainly due to some batteries running out before the finalization of an ongoing trip. Statistically significant values are marked bold. Dunn’s homogenous subgroups are indicated in superscript similar letters

### Spatial and temporal data

We downloaded tri-axial acceleration data and GPS files, comprising location (WGS84) and time, and a separate file containing dive depth and water temperature data from the recovered loggers (Table 1). Sample sizes (Table 1) varied due to logger failures that prevented to produce complete data sets for some individuals. Failures corresponded to 1) loggers damaged by salt water reaching the electronic components, 2) broken GPS antennas, and 3) batteries that were unexpectedly depleted. As in previous studies [21, 51], we defined foraging trips from the time when the birds departed from the colony to the sea until returning to the colony. To obtain bathymetric data for Antarctica, we used the International Bathymetric Chart of the Southern Ocean (IBCSO) [53], while for the Falkland/Malvinas Islands, we used bathymetry data from the global sea floor topography from satellite altimetry and ship depth soundings (Global Topography) [21, 54]. We used QGIS 3.4 (QGIS Development Team) to plot and analyse positional data of the trips performed by the birds. We calculated trip length as the total cumulative linear distance between all positional fixes along the foraging trip, outside of the colony. For each trip, we determined the maximum distance from the colony as the linear grand circle distance between the furthest point of the recorded trip and the geographical coordinates of the departure colony, determined by GPS [21, 51]. We calculated trip duration as the time difference between the onset of the first dive performed after leaving and the end of the last dive event before arriving back at the colony. For the identification of foraging dives, we used purpose-written scripts in Matlab (The Mathworks Inc., Nattick, USA) and in IGOR Pro 6.3. (WaveMetrics, Lake Oswego, USA). Following Mattern et al. [55] and in order to avoid depth measurement inaccuracy in the upper part of the water column, we accepted dive events only when depths > 3 m were reached. We defined the bottom phase as a period of the dive between a steady pressure increase at the beginning of the dive (i.e. descent) and the continuous pressure decrease indicating the penguins’ ascent back to the surface [55, 56]. We also calculated the maximum depth (in m) reached during a dive event (hereafter event maximum depth), and the number of dive events during a particular foraging trip. For each dive, we calculated a geographical position either by using the half way point between GPS fixes recorded immediately before and after the dive, or by calculating the relative position along a linear interpolated line between the last fix obtained and before the first fix after the dive occurred based on the time the dive occurred relative to these fixes. Because in previous studies we found that gentoos at New Island take both benthic and pelagic prey [21, 51], we split the foraging dives performed by the individuals in benthic and pelagic ones for further analyses. We did this by calculating the index of benthic diving behaviour developed by Tremblay & Cherel [56]. This method assumes that benthic divers dive serially to a specific depth, and therefore consecutive dives reach the same depth zone. These are called intra-depth zone (IDZ) dives [56]. As in previous studies, we defined the IDZ as the depth ± 10% of the maximum depth reached by the preceding dive [21]. During the current study, gentoos performed a varying proportion of benthic and pelagic dives, which we considered in following analyses. As the inspection of histograms showed that the data for pelagic dives was left shifted, we used the median dive depth per colony per year for further calculations involving pelagic dives (Table 2; Additional file 1, Figs. S1, S2). We show the distribution of benthic and pelagic dives in Figs. S3, S4 (Additional file 1). We also calculated the median number of dives performed during the foraging trips (Table 2). In previous studies [21, 51], we found that gentoos showed no sexual differences in foraging behaviour parameters. Gentoos from Livingston showed also no sexual differences in foraging (Additional file 1, Figs. S5). Therefore, in this study, we pooled the data of males and females. We used the nonparametric fixed kernel density estimator to determine the 50% (core area) and 95% (home range) density contour areas (estimated foraging range) [57, 58] of dive locations (i.e. GPS position at the onset of a dive event). Kernel densities indicate the places in a foraging trip where birds spent most of their time [57]. For these calculations we used both the Geospatial Modelling Environment (Spatial Ecology LLC, http://www.spatialecology.com/gme/) and QGIS 3.4 (QGIS Development Team).

**Table 2.** Dive parameters used for the calculations of energy landscapes

	Gentoo					Chinstrap
	Dec 2013	Dec 2014		Dec 2016		Jan 2017
	New I., South	New I., South	New I., North	Livingston I.		Deception I.
				Short trip	Long trip	
Maximum dive depth [m]	188.3	178.2	156.3	79.9	109.9	111.9
Median dive depth of pelagic dives [m]	15.8 ^e^ (3–185.9)	12.7 ^a,b^ (3–176.6)	21.1 ^c^ (3–156.5)	14.9 ^a^ (3–79.9)	15.4 ^b^ (3–109.9)	12.3 ^d^ (3–105.3)
Kruskal-Wallis	χ^2^ = 322.3 df = 5, ***P*** **< 0.001**					
Median proportion of benthic dives (pBD) [%]	24 (19–30)	46 (33–66)	63 (50–67)	48 (39–53)	26 (24–39)	31 (24–43)
Median proportion of pelagic dives (pPD) [%]	76 ^d^ (70–81)	54 ^a,b^ (34–67)	37 ^a^ (33–50)	52 ^a^ (47–61)	74 ^c,d^ (61–76)	69 ^b,c^ (57–76)
Kruskal-Wallis	χ^2^ = 24.6 df = 5, ***P*** **< 0.001**					
Median number of dives per foraging trip (MND)	283 ^a, c^ (202–337)	291 ^a, b, c^ (193–471)	298 ^a, b, c^ (241–331)	215 ^a^ (156–268)	402 ^b^ (299–744)	369 ^c^ (205–497)
Kruskal-Wallis	χ^2^ = 19.6 df = 5, ***P*** **= 0.002**					
Median dive duration (DD), benthic dives [s]	156 ^a^ (142–177)	155 ^a^ (150–199)	176 ^a^ (157–202)	81 ^b^ (71–96)	90 ^b^ (82–95)	70 ^c^ (60–85)
Kruskal-Wallis	χ^2^ = 61.2 df = 5, ***P*** **< 0.001**					
Median dive duration (DD), pelagic dives [s]	103 ^a^ (92–119)	123 ^a, b^ (117–125)	130 ^a^ (127–138)	67 ^c^ (63–73)	83 ^b^ (72–88)	55 ^d^ (51–69)
Kruskal-Wallis	χ^2^ = 69.8 df = 5, ***P*** **< 0.001**					
Minimum benthic bottom time (mBBT) [s]	2	3	2	2	3	2

Parameters correspond to gentoo penguins *Pygoscelis papua* breeding at New Island (Falkland/Malvinas Islands), during chick guard (December) in 2013 and 2014, gentoo penguins breeding at Devils Point, Byers Peninsula, Livingston Island, South Shetland Islands, Antarctica, during chick guard (December 2016), and chinstrap penguins *Pygoscelis antarcticus* breeding at Vapour Col rookery, Deception Island, South Shetland Islands, Antarctica, during chick guard (January 2017). Only the first foraging trip of each individual was included in the calculations in order to avoid individuals with more than one trip having more weight in the analyses. See also Figs. S1 to S4 in Additional file 1

*Notes*: Statistically significant values are marked bold. Dunn’s homogenous subgroups are indicated in superscript similar letters

As for trip and dive parameters (Tables 1 and 2) normality and equality of variance were not satisfied (*P* < 0.05; Additional file 1, Figs. S7, S8), we investigated differences using the Kruskal–Wallis test (one-way ANOVA on ranks) and Dunn’s homogenous subgroups implemented in the R package dunn.test v1.3.5 (R Development Core Team, https://www.r-project.org/) [59].

### Calculation of energy

Using tri-axial acceleration data (Additional file 1, Fig. S6), we quantified energy landscapes as the mass-specific total cost of foraging, including diving and commuting, relative to the bottom time, which we selected as a proxy of energy gained from feeding. We considered the different proportion of benthic and pelagic dives carried out by the studied penguins. With the data obtained from the deployed penguins, we calculated the energy landscapes for a grid of the marine area around the islands with the breeding colonies for which detailed bathymetric data was available. We carried out the quantification as in Masello et al. [21], to allow comparisons, and followed a series of steps.

#### Step 1, calculation of the overall dynamic body acceleration

Since the major variable factor in modulating energy expenditure in vertebrates is movement and measurements of body acceleration correlate with energy expenditure (reviewed in [60]), we used tri-axial acceleration data to calculate the Overall Dynamic Body Acceleration (ODBA) for all first foraging trips of the deployed individuals. ODBA is a linear proxy for metabolic energy that can be further converted into energy expenditure [16, 23, 60, 61] but see also [62]. As in previous studies [21, 51], only the first foraging trip of each individual was included in the calculations to avoid individuals with more than one trip having more weight in the analyses, and to allow comparisons.

We calculated ODBA (expressed as gravitational force *g*) using a purpose-written script for IGOR Pro 6.3 (WaveMetrics, Lake Oswego, USA) and the sum of the absolute values of dynamic acceleration from each of the three spatial axes (i.e. surge, sway, and heave; sampling interval: 50 Hz) after subtracting the static acceleration (= smoothed acceleration; smoothing window: 1 s) from the raw acceleration values following Wilson et al. [23]:

1 $$ \mathrm{ODBA}=\left| Ax\right|+\left| Ay\right|+\left| Az\right| $$

A_x_, A_y_ and A_z_ are the derived dynamic accelerations at any point in time corresponding to the three orthogonal axes of the Axy-2 or the Axy-Trek acceleration loggers deployed on the penguins.

#### Step 2, calculation of benthic and pelagic ODBAs

In diving seabirds, power costs during dive vary with the depth exploited [63, 64], and penguins take both benthic and pelagic prey [21, 51, 65]. For both reasons, we split the foraging dives performed by the individuals in benthic and pelagic ones, calculated the corresponding benthic and pelagic ODBAs, and interpolated them for the available bathymetric data points around the breeding colonies.

For this step, we first investigated the relationship between the ODBAs calculated in Step 1 and penguins’ maximum dive depth. We found that the sum of ODBA during the dives carried out by the penguins was related to the maximum dive depth they reached (0.70 < *R*^2^ < 0.78; see also Additional file 1, Figs. S9-S12). However, using a general additive model implemented in the R package GAM [66] we found that this relationship differed between benthic and pelagic dives both for gentoos and chinstraps (Additional file 1, Table S1). Thus, we determined the regressions with the best fit for the different dive types, benthic and pelagic, in SigmaPlot 10 (Systat Software, San Jose, USA). We provide the regression descriptions and corresponding parameters in Table S2 (Additional file 1). We used the regressions between the sum of ODBA during the dive of the deployed penguins and the maximum dive depth (Additional file 1, Table S2), together with the bathymetric data points from IBCSO [53] to calculate benthic ODBAs for a grid of the marine area around the penguin colonies (approximately 100 km around the islands; *n* = 8130; grid spatial resolution as in IBCSO: 500 × 500 m, based on a polar stereographic projection) separately for each species. To calculate the pelagic ODBA, we used the regressions (Additional file 1, Table S2) and the median dive depth (Table 2), as pelagic dive depth data were not normally distributed but left-shifted.

#### Step 3, calculation of the cost of travelling

In seabirds like penguins, which cover large distances to reach their foraging grounds, it is important to include the energy cost of travelling for any calculations of the cost of foraging. In previous work [21, 51], we found that gentoos performed foraging trips of up to 282 km, while up to 139 km were reported for chinstraps [67]. We first calculated the distance between each point in the marine area grid around the islands with the penguin breeding colonies (see Step 2) with the Geospatial Modelling Environment and QGIS 3.4. Using this distance and the mean swimming speed previously calculated for gentoos (2.3 m s^− 1^) [68], we were able to calculate the travel time needed for the birds to reach each of the 8130 locations around the islands for which bathymetric data were available. The travel time (TT, in s), and their minimum metabolic cost of transportation previously determined in a swim canal and at sea (16.1 W kg^− 1^) [68, 69], allowed us subsequently to calculate the minimum cost of travelling (CT, in J kg^− 1^) to each location in the grid used to construct the energy landscapes:

2 $$ \mathrm{CT}=\mathrm{TT}\ast 16.1\kern0.5em \mathrm{W}\kern0.5em {\mathrm{kg}}^{\hbox{-} 1} $$

#### Step 4, calculation of the cost of a dive

To quantify the cost of a dive, including the cost of the pursuit of prey during a dive, we first had to measure its energy expenditure. The rate of oxygen consumption V_o_ (in ml min^− 1^) is an indirect measure of energy expenditure commonly used under laboratory conditions (for examples see [60]) but difficult, if not impossible, to use in diving seabirds like penguins. An alternative technique for free-ranging animals is to use ODBAs as a calibrated proxy for the rate of oxygen consumption V_o_ [61, 70], which can be used to calculate the total energy expenditure during a dive.

Previous research demonstrated a linear relationship between ODBA and energy expenditure in all species examined to date (summarised in [23]; but see [62, 71]). Following the method developed by Wilson et al. [70] and tested by Halsey et al. [61] in several species, we first calculated V_o_:

3 $$ {\mathrm{V}}_{\mathrm{o}}=9.16+\mathrm{ODBA}\ast 16.58\left(\mathrm{for}\ \mathrm{gentoos}\right)\mathrm{or}\ {\mathrm{V}}_{\mathrm{o}}=7.15+\mathrm{ODBA}\ast 12.04\left(\mathrm{for}\ \mathrm{chinstraps}\right) $$

We calculated the intercept and slope in (3) also following Halsey et al. [61]. These authors found that the intercept and the slope for the relationship between ODBA and V_o_ (in ml * min^− 1^) in all species studied could be calculated as: intercept, y = 2.75 * BM^0.73^ (*R*^2^ = 0.89), slope, y = 3.52 * BM^0.94^ (*R*^2^ = 0.94), with BM being the mean adult body mass in kg.

The uptake of 1 l of oxygen can be converted into an energy expenditure estimate of approximately 20 kJ [72], such that 1 ml O_2_/min equals 0.333 J s^− 1^. Finally, to derive the energy expenditure (in J kg^− 1^ s^− 1^) relative to the body mass of the penguins (also called mass-specific power, MP, e.g. [21, 23]), we divided the energy expenditure by the mean weight of the penguins (gentoos: 5.2 kg; chinstraps: 3.7 kg; individuals measured in this study):

4 $$ \mathrm{MP}={\mathrm{V}}_{\mathrm{o}}\ast 0.333/\mathrm{BM} $$

The equation in (4) allowed us to calculate the MP separately for benthic dives (MP_benthic_, using benthic ODBA from Step 2 in Eq. 3) and pelagic dives (MP_pelagic,_ using pelagic ODBA from Step 2 in Eq. 3) for each point in the grid around the islands used to construct the energy landscapes.

#### Step 5, integrating the cost of the actual number of dives performed

Subsequently, we calculated the MP for each point of the marine area’s grid around the islands with the studied breeding colonies for the number of benthic and pelagic dives carried out by the penguins. In the case of chinstraps, we used the median number of dives per foraging trip (MND; Table 2) together with the mean dive duration (DD, duration in s of the dive event; Table 2), assuming a gradient of bottom depths from 3 m (minimum depth consider a dive, see the justification in *Spatial and temporal data*) to the maximum depth (= bathymetric depth) for benthic dives, and a gradient of bottom depths from 3 m to median dive depth for pelagic dives as follows:

5 $$ {\mathrm{MP}}_{\mathrm{MND}\ \mathrm{benthic}}={\mathrm{DD}}_{\mathrm{benthic}}\ast \left({\mathrm{MP}}_{\mathrm{benthic}\left(3\ \mathrm{m}\ \mathrm{depth}\right)}+{\mathrm{MP}}_{\mathrm{benthic}}\right)\ast \mathrm{MND}/2\ast \mathrm{pBD} $$

6 $$ {\mathrm{MP}}_{\mathrm{MNDpelagic}}={\mathrm{DD}}_{\mathrm{pelagic}}\ast \left({\mathrm{MP}}_{\mathrm{pelagic}\left(3\ \mathrm{m}\ \mathrm{depth}\right)}+{\mathrm{MP}}_{\mathrm{pelagic}}\right)\ast \mathrm{MND}/2\ast \mathrm{pPD} $$

where pBD is the mean proportion of benthic dives and pPD the mean proportion of pelagic dives (Table 2), included accounting for the proportion of benthic and pelagic dive in a single foraging trip.

In the case of gentoos, which in addition to pelagic and benthic dives performed short and long trips and showed a relationship between the number of dives and the maximum distance from the colony during a foraging trip (Additional file 1, Fig. S13), we used the regression in Table S3 (Additional file 1) to compute MND.

#### Step 6, integrating the cost of diving and commuting

The parameters calculated in Step 5, together with previous calculations of CT (Step 4), allowed us to calculate the total cost of foraging (TCF, in J kg^− 1^) as:

7 $$ \mathrm{TCF}={\mathrm{MP}}_{\mathrm{MND}\ \mathrm{benthic}}+{\mathrm{MP}}_{\mathrm{MND}\ \mathrm{pelagic}}+\mathrm{CT}\ast 2 $$

CT is multiplied by two to account for the return to the breeding colony.

#### Step 7, calculating the energy gained during foraging

Previous studies on several penguin species have found a positive relationship between bottom times (duration in s of bottom dive phase) and prey capture: Southern rockhoppers *Eudyptes chrysocome* have been found to maximise bottom time, which in this species equalled feeding time [56]; chinstraps showed a positive linear relationship between bottom time and the number of underwater beak-opening events during dives, and that most (86%, *n* = 4910 events) of beak-openings occurred during the bottom times [73]; king *Aptenodytes patagonicus* and Adélie *Pygoscelis adeliae* penguins ingested prey mostly during the bottom phase of diving [74]; and little penguin *Eudyptula minor* showed longer bottom times associated with dives where prey was captured [75]. Thus, several studies have successfully used bottom time as a proxy for prey acquisition and energy gained both in penguins [21, 76] and other seabirds [77]. To build energy landscapes that also include the energy gained during foraging, we calculated bottom times and minimum benthic bottom times (mBBT; Table 2). The bottom times from the first foraging trip of each individual showed a relationship with maximum dive depth. This relationship also differed between benthic and pelagic dives (GAM; Additional file 1, Table S4). Again here, we determined the regressions with the best fit for the different dive types in SigmaPlot 10.0 (Additional file 1, Table S5; Figs. S14-S17). The regressions between bottom time and maximum dive depth (Additional file 1, Table S5), allowed us to calculate the sum of benthic bottom time (BBT) for each point of the grid of the marine area around the islands with the studied breeding colonies used to construct the energy landscapes, separately for each species. For pelagic bottom times (PBT), we used the corresponding regressions (Additional file 1, Table S5) and the median dive depth per species (Table 2). To calculate the total bottom time (TBT, in s), we took into account that the birds start diving close to the colony (as also found in [21, 51]) and increase dive depth while gaining distance. A mean is calculated and the mean multiplied per MND:

8 $$ \mathrm{TBT}=\left(\mathrm{mBBT}+\mathrm{BBT}\right)/2\ast \mathrm{MND}\ast \mathrm{pBD}+\mathrm{PBT}\ast \mathrm{MND}\ast \mathrm{pPD} $$

We also included pBD and pPD here to account for the proportion of benthic and pelagic dive in a single foraging trip.

#### Step 8, construction of the energy landscapes

Finally, dividing TCF (7) by TBT (8), we were able to calculate the total relative cost (TRC, in J kg^− 1^ s^− 1^), which is the mass-specific total cost of foraging (diving plus commuting) relative to the energy gained. Using TRC values calculated for the grid of the marine area around the islands with the breeding colonies, we constructed the energy landscape by applying the inverse distance weighted (IDW) interpolation in to the resulting data grid. As in our previous study [21], the IDW interpolation was chosen as 1) a large set of sample values was available, and 2) the sample data points represented the minimum and maximum values in our surface [78]. In brief, the energy landscapes here presented are based on the bathymetry of the area and the total cost of foraging (diving plus commuting) relative to the bottom time (= energy gained, in J kg^− 1^ s^− 1^), and take into account the different proportion of benthic and pelagic dives carried out by the penguins.

### Molecular analysis of the diet

We collected a total of 247 faecal samples from gentoos from the colony at Livingston, chinstraps from the colony at Deception, two colonies at New Island, and potential prey samples to obtain detailed information on diet composition (Additional file 1, Tables S6 and S7). Details on deoxyribonucleic acid (DNA) extraction, primers used, polymerase chain reaction (PCR) amplifications, library preparations, and next generation sequencing (NGS) are provided in the Additional File 1 (Table S8 and Additional Methods).

We used the raw Illumina sequence data to produce a list of molecular operational taxonomic units (MOTUs). Bioinformatics analyses included the following steps: assessing sequence quality with FASTQC (http://www.bioinformatics.babraham.ac.uk/projects/fastqc), adapter and quality trimming of the paired-end reads with TRIMMOMATIC (minimum quality score of 20 over a sliding window of 4 bp) [79], merging of the overlapping paired-end read pairs using FLASH [80], transforming sequence files to FASTA with the FASTX-Toolkit (http://hannonlab.cshl.edu/fastx_toolkit/), and extracting amplicons in MOTHUR [81]. We used USEARCH [82] to remove identical replicates (dereplicate; derep_fulllength), to detect and to remove chimeric sequences (uchime_denovo) and to cluster sequences into molecular operational taxonomic units (MOTUs). Using the BLASTn algorithm [83] we matched MOTU sequences to reference sequences in the National Center for Biotechnology Information (NCBI) GenBank nucleotide database, using a cut-off of 90% minimum sequence identity and a maximum e-value of 0.00001. For the bioinformatics analyses of the samples from Antarctica, we carried out all those analyses using a custom workflow in GALAXY (https://www.computational.bio.uni-giessen.de/galaxy) [84]. As next step, we manually discarded MOTUs that corresponded to regular fieldwork contaminants in faecal samples, such as bacteria, soil fungi, human or predator DNA. We based taxonomic assignment on the percentage similarity of the query and the reference sequences. Since short fragments are less likely to contain reliable taxonomic information, we only retained sequences with a minimum length of 190 bp and a BLASTn assignment match greater than 98% [85, 86]. We assigned MOTUs to species-level in cases when all retained hits of a MOTU with the same quality criteria (sequence identity, sequence length, e-value) corresponded to the same species, if not we assigned the MOTU to the lowest shared taxonomic level, e.g. genus or family, as in Kleinschmidt et al. [87]. We performed further filter steps to avoid contamination/false positives and to obtain reliable data [88] as follows: we accepted MOTUs in a sample only if they contained a minimum of 10 sequences or accounted for > 1% of the maximum total of hits. Additionally, we also discarded taxa with very distant or ecologically irrelevant distribution ranges (e.g. deserts). Negative controls were included and did not show any contaminations. For each taxonomical level found, we calculated the frequency of occurrence (FO) [89]. To visualize differences in diet compositions for the penguin species and for adults and chicks, we performed non-metric multidimensional scaling (NMDS) with the function *metaMDS* in the R package VEGAN [90]. NMDS uses rank orders to collapse information from multiple dimensions into usually two-dimensions to facilitate visualization and interpretation, and is generally considered as the most robust unconstrained ordination method in community ecology [91, 92]. The function *metaMDS* allowed us to investigate the agreement between the two-dimension configuration and the original configuration through a stress parameter. If the stress is < 0.05 the agreement is excellent, < 0.1 is very good, < 0.2 provides a good representation. In our models the stress was always < 0.04 (excellent). We performed permutational multivariate analysis of variance using distance matrices (PERMANOVA) with the function *adonis* and checked for the multivariate homogeneity of group dispersions (variances) with the function *betadisper*. We also used the functions *ordihull* and *ordiellipse* to add convex hulls and ellipses to the NMDS plots and improve visualization. To compare the diet composition for a certain number of sampled individuals, we additionally used species accumulation curves (SAC) with the function *specaccum* in the R package VEGAN [90].

### Stable isotope analysis of the diet

We analysed carbon (δ^13^C) and nitrogen (δ^15^N) stable isotope ratios of red blood cells. Stable isotope ratios allowed us to compare the diet the penguins fed during the study period, as red blood cells have a half-life of ca. 30 days [93]. We carried out carbon and nitrogen isotope analyses on 0.65–0.75 mg sample aliquots, weighed into tin cups. Subsequently, we determined carbon and nitrogen isotope ratios by a mass spectrometer (Delta V Plus with a Conflo IV interface, Thermo Scientific, Bremen, Germany) coupled to an elemental analyser (Flash 2000, Thermo Scientific, Milan, Italy) at the LIENSs laboratory from the University of La Rochelle, France. Replicate measurements of internal laboratory standards indicated measurement errors < 0.15 ‰ for δ13C and δ15N. Results are expressed in the δ unit notation as deviations from standards (Vienna Pee Dee Belemnite for δ^13^C and N_2_ in air for δ^15^N) following the formula: δ^13^C or δ^15^N = [(R_sample_/R_standard_) - 1] × 10^3^, where R is ^13^C/^12^C or ^15^N/^14^N, respectively. Internal laboratory standards (acetanilide) were used to check accuracy. Measurement errors were < 0.15‰ for both δ^13^C and δ^15^N.

We compared the isotopic niches of penguins using SIAR (Stable Isotope Analyses in R) [94] and SIBER (Stable Isotope Bayesian Ellipses in R) [95]. The location of the centroid (mean δ^13^C, mean δ^15^N) indicates where the niche is centred in isotope space. We used a Bayesian approach based on multivariate ellipse metrics to calculate the Bayesian standard ellipse area (SEAb), which represents the core isotope niche width as described by Jackson et al. [95]. In addition, we calculated standard ellipse areas based on Maximum Likelihood (SEA), and corrected for sample size (SEAc). We depicted ellipses using the draw.ellipse command of the R package PLOTRIX [96], with the lengths of the two semi-major axes and the angle of the semi-major axis of the ellipse with the x-axis as parameters. To describe the spread of the data points, we calculated parameters as described by Layman et al. [97]. As proxies of intra-population trophic diversity, we also calculated the mean distance to centroid (CD) and the mean nearest-neighbour distance (NND). We give information on the trophic length of the community as the δ^15^N range (NR), and provide an estimate of the diversity of basal resources by the δ^13^C range (CR). We split the data from gentoos into male and female adults, and first and second hatched chicks but, due to low samples size, we were not able to split chinstrap data.

### Condition parameters

The ratio of two types of leucocytes, the heterophils and lymphocytes (H/L ratio), has been successfully used as an indicator of physiological status and effort (high ratios = high stress) [98, 99]. Following Merino et al. [100], differential leucocyte counts were carried out with a light microscope (× 1000) in parts of the blood smears where erythrocytes had separated in a monolayer. The samples were crossed from down to up to minimize differences in the thickness of the blood smear. Leucocytes were counted following Dein [101] and Hawkey and Dennett [102]. A total of 100 leucocytes were counted in each smear, thus obtaining percentages of the different of leucocyte types and the H/L ratio.

### Additional data

We obtained the location of other gentoo and chinstrap penguin colonies in the South Shetland Islands, Antarctica, from the Mapping Application for Penguin Populations and Projected Dynamics [103] and Naveen et al. [104], and the locations of Fur Seal *Arctocephalus gazella* colonies from Hucke-Gaete et al. [105]. We downloaded Antarctic Krill *Euphausia superba* abundance data for the sector between 60 and 65°S and 55–65°W from KRILLBASE [106], and obtained Antarctic Krill catches for the Commission for the Conservation of Antarctic Marine Living Resources (CCAMLR) Area 48 from the Krill Fishery Report 2018 [107]. Breeding success data corresponds to the number of chicks per nest at the crèche, and was obtained as part of ongoing projects (Vapour Col rookery, Deception, [43, 108] and AB unpubl. Data; New Island, [21] and PQ unpubl. Data;) or from studies in the West Antarctic Peninsula region that followed the same methodology we used (Petermann Island, [109]; Goudier Island, [110]). Other available studies for the region were excluded, as their methodology clearly differed from the one here used. Due to logistics limitations of our expedition to Antarctica, breeding success data at Livingston, could not be gathered.

## Results

### Foraging trips and dive parameters

**Fig. 2:**
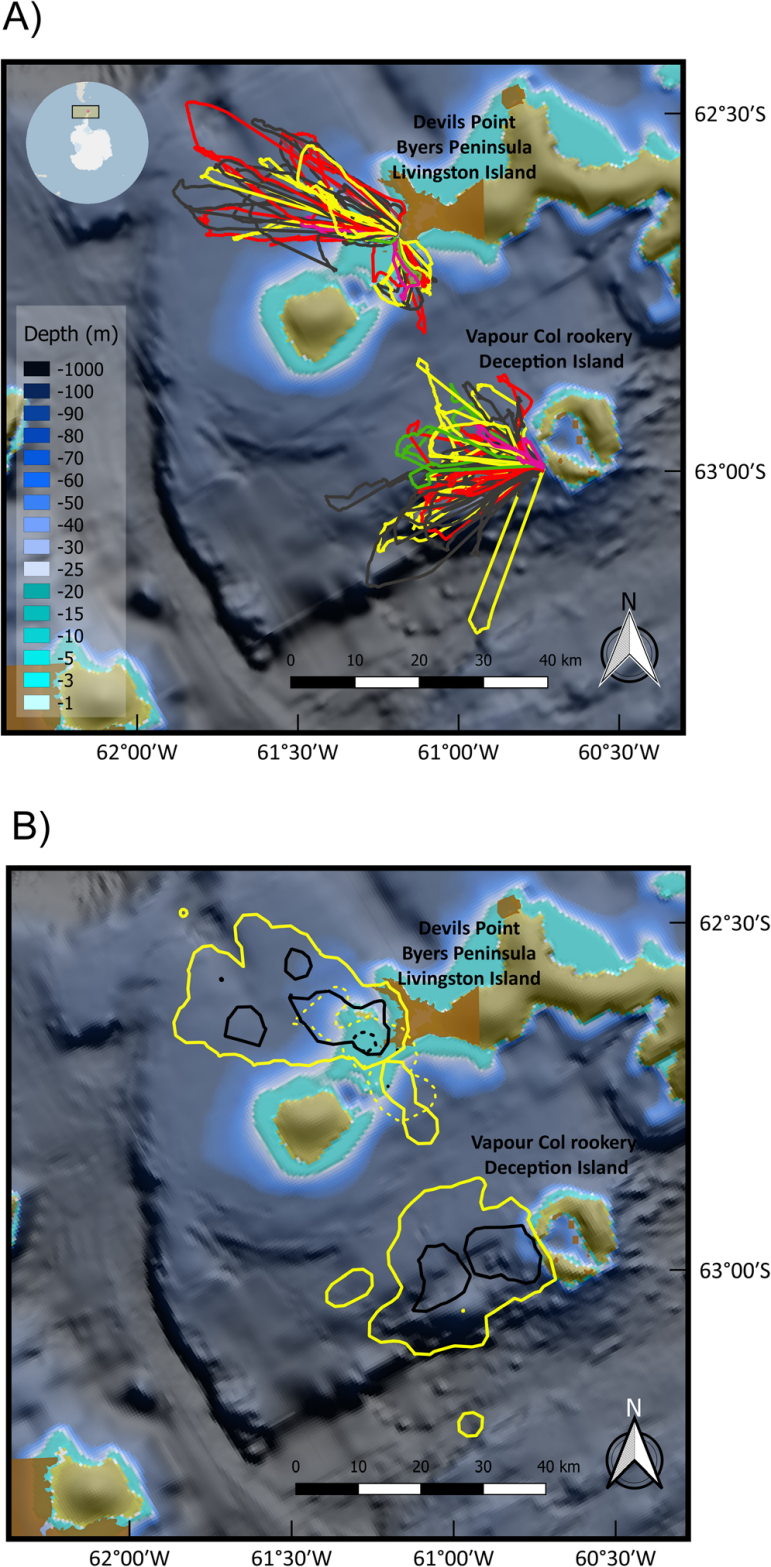
Foraging trips (**a**) and kernel density distribution of dive locations (**b**). Data from gentoo penguins *Pygoscelis papua* breeding at Devils Point, Byers Peninsula, Livingston Island, South Shetland Islands during chick guard (December 2016), and chinstrap penguins *Pygoscelis antarcticus* breeding at Vapour Col rookery, Deception Island, South Shetland Islands, during chick guard (January 2017). Trip lines are colour coded. Dark grey: first recorded trips, red: second trips; yellow: third trips, green: fourth trips; pink: fifth trips. The 50% core areas are denoted by black lines, while 95% home ranges by yellow lines. Kernels from gentoo penguins are further coded for short (dashed lines) and long trips (solid lines). Kernels from chinstrap penguins are denoted by solid lines only, as no distinction between short and long trips could be found. Depth (in m) is based on data from the International Bathymetric Chart of the Southern Ocean (IBCSO) [53]

In Antarctica, both gentoos and chinstraps foraged relatively close to their own colonies (Fig. 2), using the colony’s ‘hinterland’ (sensu Cairns [111]) and hence, avoided areas closer to the neighbouring colonies and those from potential predators (Additional file 1, Fig. S18), and performed trips with the usual loop shape (Fig. 2). Gentoos from Livingston performed short (19.9–33.4 km) and long (59.2–71 km) trips, which strongly differed in both length (median, short trip: 27.1 km, long trips 66.6 km; Table 1, Fig. 2a), and in the extent of the core areas and home ranges used (Fig. 2b; Additional file 1, Fig. S19). The short trips carried out by gentoos from Livingston were shorter than any of the trips performed by New Island birds (minimum trip: 40.8 km), while the long trips were similar to those carried out by New Island birds in 2014 (median, South: 88.7, North: 59.1 km) but substantially different than the much longer trips performed by New Island birds during 2013 (median, 125.6 km; Table 1). The trips performed by chinstraps from Deception (median 37.7 km) were intermediate between the long and short trips from gentoos from Livingston (Table 1). Other related trip parameters are provided in Table 1.

In Antarctica, the maximum dive depth was recorded in chinstraps (111.9 m, Table 2). However, maximum dive depth achieved by both gentoos (109.9 m) and chinstraps from Antarctica were lower than those from gentoos from New Island (188.3 m, Table 2), regardless of the much deeper waters present in marine areas close to Livingston and Deception (up to 1000 m depth, Fig. 2). When we considered the depth of the pelagic dives separately, we found that chinstraps dived less deep (median: 12.3 m) than gentoos (median, long trips: 15.4 m, short trips: 14.9 m). This is in line with the higher proportion of benthic dives carried out by chinstraps (31%) in comparison with gentoo long trips (26%). Gentoos from Livingston carried out the highest number of dives per foraging trip during their long trips (median: 402 dives) followed by the chinstraps (369 dives). During short trips, gentoos from Livingston carried out a similar number of dives per foraging trip (215 dives) as the birds from New Island (medians ranging from 283 to 298 dives, Table 2).

### Calculation of energy

**Fig. 3:**
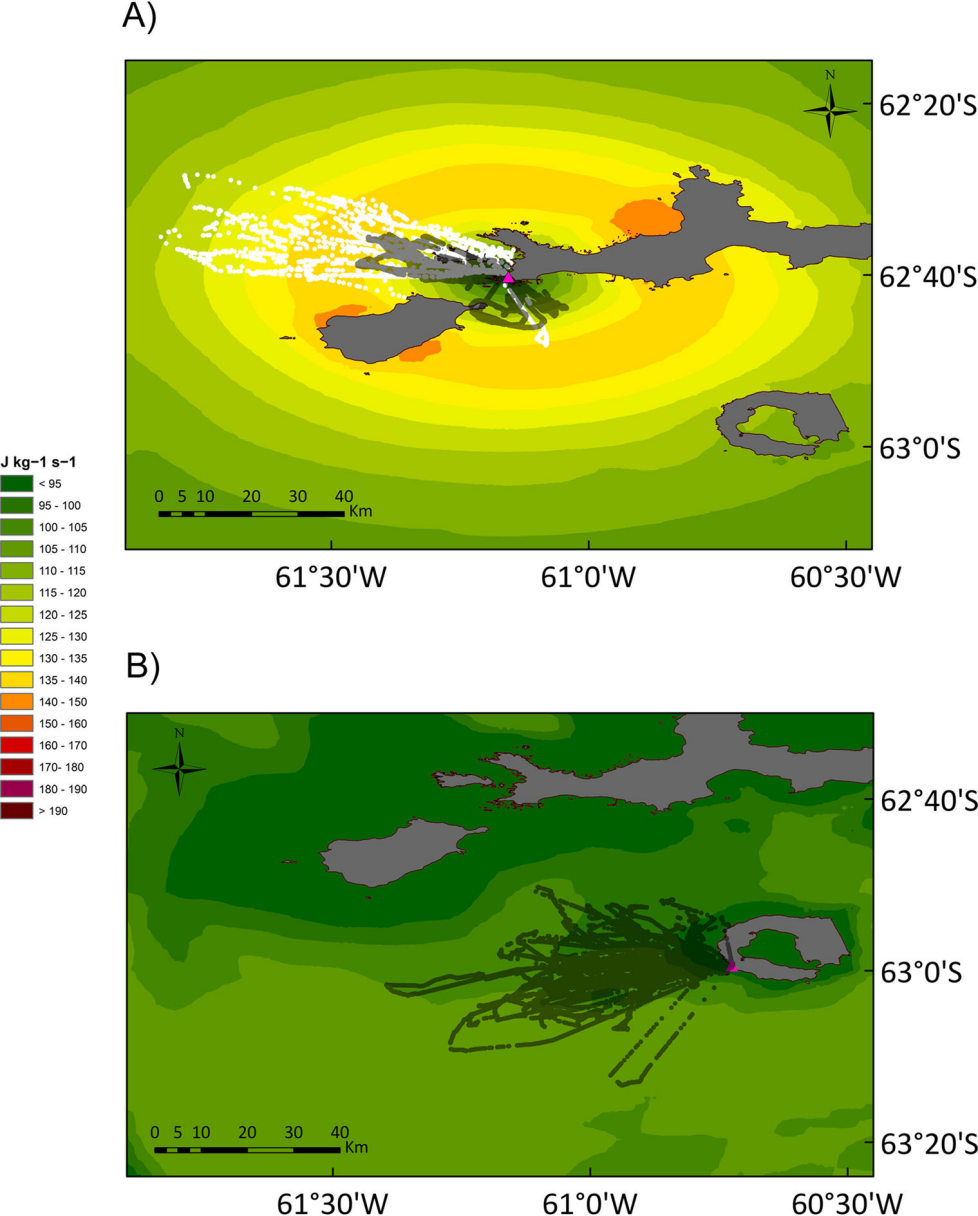
Gentoo and chinstrap penguin energy landscapes. Energy landscapes based on the bathymetry around Livingston Island, South Shetland Islands and the mass-specific total cost of foraging (diving plus commuting) by gentoo penguins *Pygoscelis papua* (**a**) and chinstrap penguins *Pygoscelis antarcticus* (**b**) relative to the bottom time (in J kg^−1^ s^−1^), considering the different proportion of benthic and pelagic dives carried out by the penguins. The energy landscape categories have been defined to make them easy comparable with the ones used for gentoo penguins from New Island, Falkland/Malvinas Islands, in [21]. The colony is marked by a triangle. The location of the dives performed by the tracked birds is plotted in semi-transparent black circles for those corresponding to gentoo short trips, and in white circles for those corresponding to gentoo long trips, and semi-transparent black circles for chinstraps

**Fig. 4:**
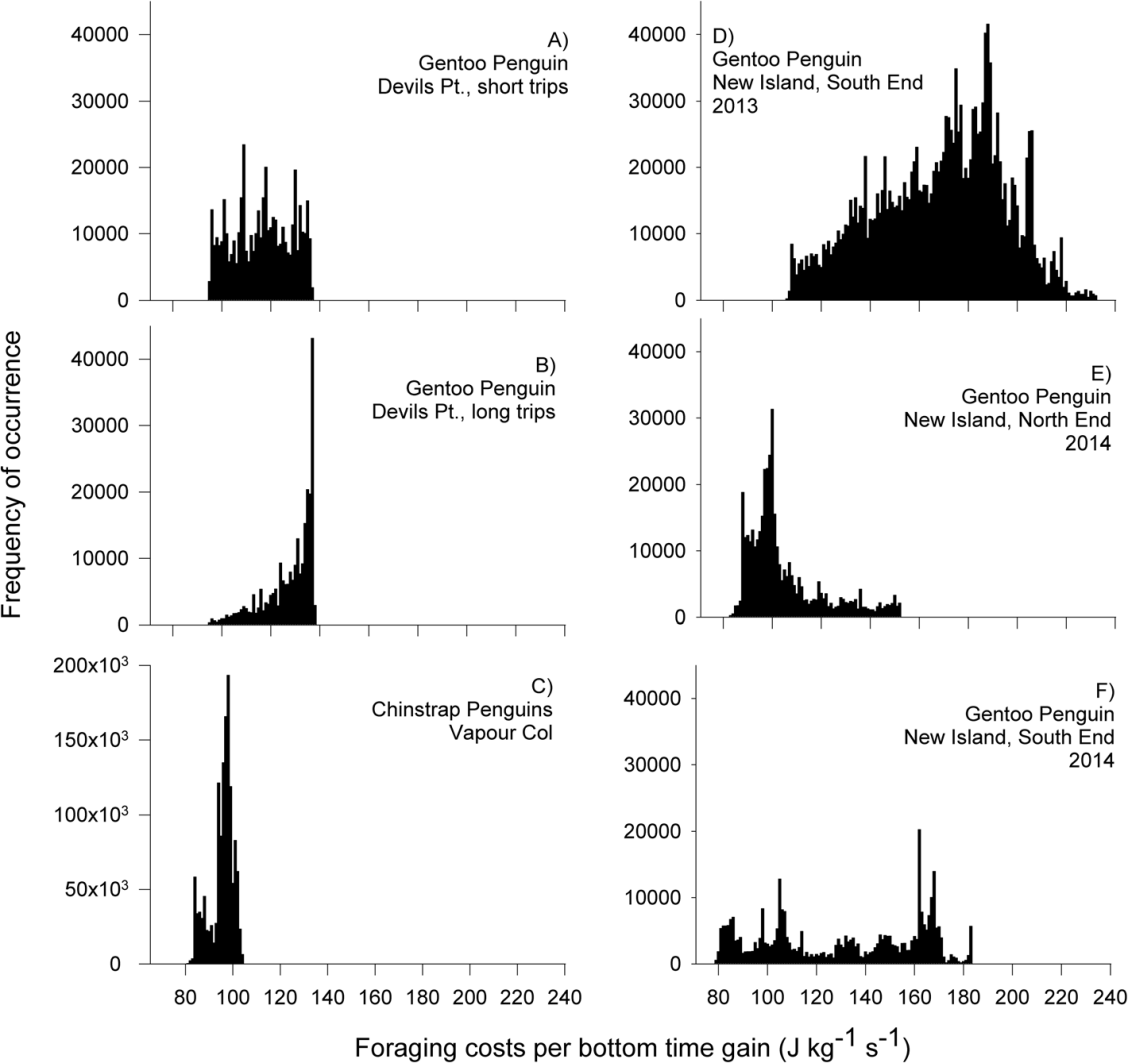
Frequencies of foraging costs per bottom time gain. Data are shown in J kg^-1^ s^-1^, for short (**a**) and long trips (**b**) carried out by gentoo penguins *Pygoscelis papua* breeding at Devils Point, Byers Peninsula, Livingston Island, South Shetland Islands during chick guard (December 2016), chinstrap penguins *Pygoscelis antarcticus* (**c**) breeding at Vapour Col rookery, Deception Island, South Shetland Islands, during chick guard (January 2017), and for gentoo penguins, breeding on New Island, Falkland / Malvinas Islands, at the South End colony, during the 2013 breeding season (**d**), at the North End colony during 2014 (**e**), and at the South End colony during 2014 (**f**)

Gentoos from Livingston used areas of the energy landscape that resulted in the lowest foraging costs relative to energy gain during foraging (up to 137.6 J kg^− 1^ s^− 1^), avoiding areas equally distant where the costs were higher (150 to 160 J kg^− 1^ s^− 1^, Fig. 3a). Moreover, the energy landscapes in the marine areas around Livingston (Fig. 3a) implied much lower costs than those around New Island (up to 232 J kg^− 1^ s^− 1^, Additional file 1, Figs. S20 to S22). During short trips, gentoos from Livingston incurred in foraging costs per bottom time gain with a median value of 115.2 J kg^− 1^ s^− 1^ (94.9 to 136.7, Fig. 4a). The median foraging cost per bottom time gain during the long trips performed by gentoos from Livingston was 130.5 J kg^− 1^ s^− 1^ (95.3 to 137.6, Fig. 4b). In the case of New Island, gentoos incurred in variable foraging costs per bottom time gain: 1) South End colony 2013, 167.1 (106.1 to 232.0; Fig. 4d), 2) South End 2014, 112.7 (78.7 to 183.1, Fig. 4e), 3) North End 2014, 99.0 (82.9 to 151.9, Fig. 4f) (medians and ranges in J kg^− 1^ s^− 1^). In this way, the foraging costs per bottom time gain of the short trips were lower than those of the long trips, while those from New Island South 2013 were the highest and those from New Island North End 2014 the lowest (Kruskal-Wallis χ^2^ = 23,852, d.f. = 5, *P* < 0.001; pairwise analyses in Additional file 1, Table S9).

Chinstraps used marine areas around Deception where the foraging costs per bottom time gain were below 105 J kg^− 1^ s^− 1^ (median 96.5, range 80.8 to 103.7; Figs. 3b and 4c). Chinstraps incurred significantly lower foraging costs per bottom time gain than the gentoos from Livingston (up to 137.6 J kg^− 1^ s^− 1^) or those from New Island South 2013 and 2014 (up to 232) but similar to those from New Island North 2014 (up to 151.9) (pairwise Kruskal-Wallis rank sum test in Additional file 1, Table S9; all *P*-values < 0.001 except for New Island North, *P* = 0.364).

### Molecular analysis of the diet

**Fig. 5:**
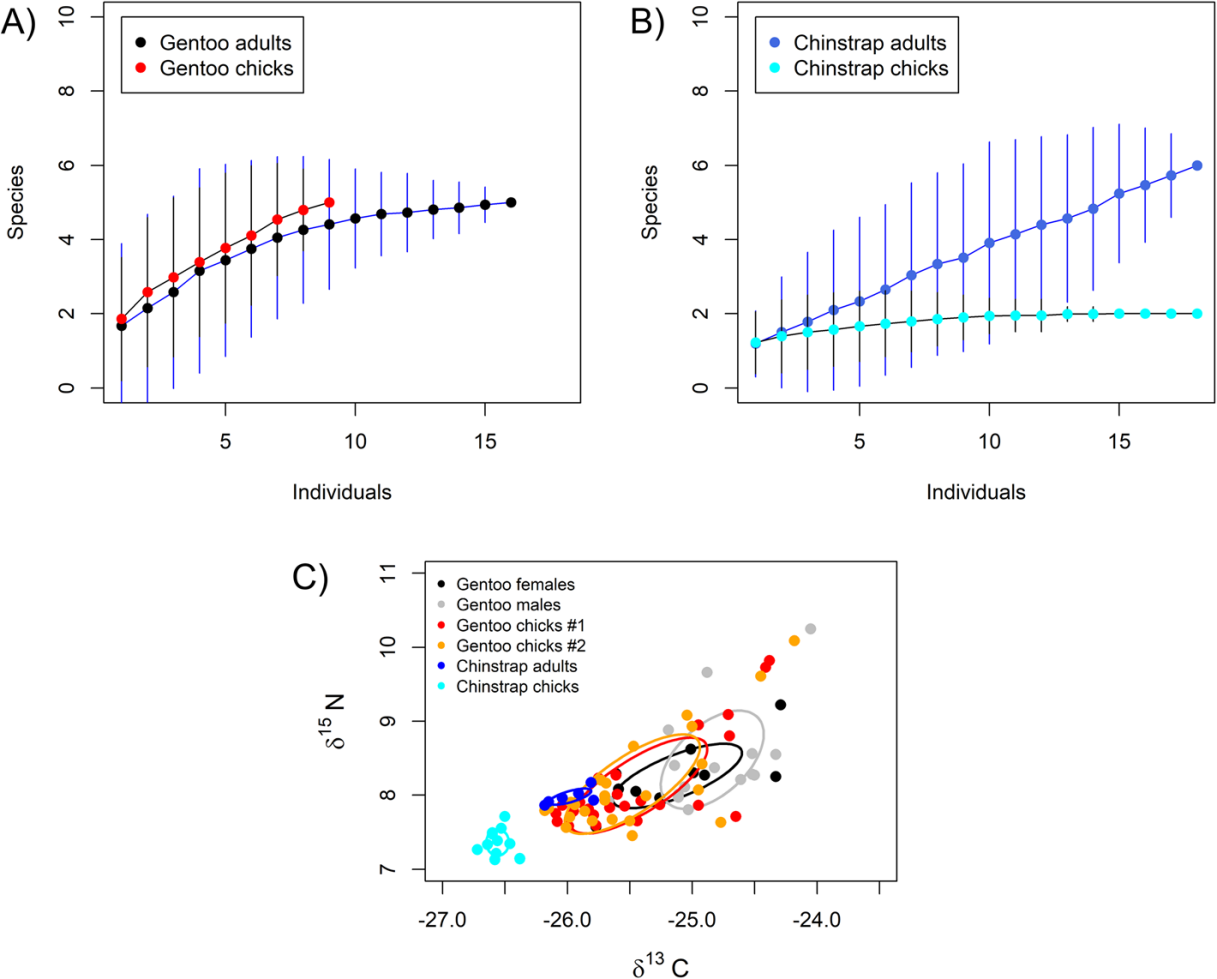
Prey consumed by the penguins. **a** shows the species accumulation curves corresponding to the prey consumed by adult and chick gentoo penguins *Pygoscelis papua* from the colony at Devils Point, Byers Peninsula, Livingston Island, South Shetland Islands during chick guard (December 2016), and **b** by adult and chick chinstrap penguins *Pygoscelis antarcticus* breeding at Vapour Col rookery, Deception Island, South Shetland Islands, during chick guard (January 2017). **c** illustrates the isotopic niches based on δ^13^C and δ^15^N. Values were measured in red blood cells of adult and chick gentoo penguins from the colony at Devils Point, and of adult and chick chinstrap penguins *Pygoscelis antarcticus* breeding at Vapour Col rookery

Gentoos from Livingston (Antarctica) and from New Island (sub-Antarctic) consumed different prey, with the birds from Antarctica consuming a less diverse diet (Table 3, Additional file 1, Table S10). When considering the quantitative data from Antarctic penguins, we found that chinstraps had a more restricted diet than gentoos preying mainly on Antarctic Krill, while gentoos from Antarctica, in addition to Antarctic Krill, included fish more frequently (NMDS: *F*_60,1_ = 3.7, *P* < 0.023, where the species explained 6% of the overall variation, *R*^2^ = 0.059; Table 3; Additional file 1, Fig. S23). When considering age in our analyses, we found that the diet composition differed among the groups (adult gentoo, chick gentoo, adult chinstrap, chick chinstrap; *F*_60,3_ = 2.2, *P* = 0.028; *R*^2^ = 0.10; Additional file 1, Fig. S24). Gentoo chicks had a slightly richer diet composition than adults, as they were fed more frequently with fish (Fig. 5a, Table 3). In chinstraps, chicks were fed more frequently with *Thysanoessa macrura*, which is taken only very occasionally in adults, and adults had a richer diet composition by consuming occasionally some fish (Fig. 5b, Table 3). However, permutation tests were not significant when gentoos (*F*_24,1_ = 1.4, *P* = 0.203, *R*^2^ = 0.059) or chinstraps (*F*_35,1_ = 1.1, *P* = 0.254, *R*^2^ = 0.032) were analysed apart (Additional file 1, Figs. S25, S26).

**Table 3:** Detected prey and their frequency of occurrence in scat samples

Taxa	Gentoo									Chinstrap		
	Dec 2013				Dec 2014		Dec 2016			Jan 2017		
	New I., South				New I., South	New I., North	Livingston I.			Deception I.		
	Adults	First chicks	Second chicks	Unk.	All ^a^	All ^a^	Adult (%)	First chicks (%)	Second chicks (%)	Adults (%)	First chicks (%)	Second chicks (%)
	*n* = 17 (17)	*n* = 24 (19)	*n* = 15 (14)	*n* = 17 (14)	*n* = 32 (31)	*n* = 29 (29)	*n* = 36 (16)	*n* = 11 (6)	*n* = 5 (3)	*n* = 19 (18)	*n* = 23 (10)	*n* = 23 (8)
Arthropoda, Crustacea, Malacostraca												
Amphipoda, Hyperiidae												
*Themisto* sp.	–	–	–	D	D	D	–	–	–	–	–	–
Decapoda												
Pandalidae (shrimps)												
*Pandalus* sp.	–	–	–	D	D	D	–	–	–	–	–	–
Galatheidae												
*Munida gregaria* lobster krill	–	–	–	D	D	D	–	–	–	–	–	–
Euphausiacea												
*Euphausia superba* Antarctic krill	–	–	–	–	–	–	100	100	100	100	100	100
*Thysanoessa macrura*	–	–	–	–	–	–	25	50	33	1	10	25
Mollusca, Cephalopoda												
Octopoda												
*Enteroctopus megalocyathus* Southern red octopus	–	–	–	D	–	–	–	–	–	–	–	–
Oegopsida												
Onychoteuthidae (squids)												
*Moroteuthis* sp.	D	–	–	D	D	D	`-	–	–	n	–	–
Ommastrephidae (squids)	–	–	–	–	–	D	–	–	–	–	–	–
Pyroteuthidae (fire squids)	–	–	–	–	D	–	–	–	–	–	–	–
Sepida												
Sepiolidae (bobtail squids)	–	–	–	–	–	D	–	–	–	–	–	–
Chordata, Vertebrata, Actinopterygii, Teleostei												
Clupeiformes												
Clupeidae												
*Sprattus* sp. (spratts)	D	D	D	D	D	D	–	–	–	–	–	–
Gadiformes												
Gadidae (codfishes)	D	D	D	D	D	D	–	–	–	–	–	–
*Micromesistius* sp. (blue whitings)	D	D	D	D	D	D	–	–	–	–	–	–
Myctophiformes												
Myctophidae	D	–	–	–	–	–	–	–	–	–	–	–
*Electrona* sp.	–	–	–	D	–	–	–	–	–	–	–	–
*Electrona antarctica* Antarctic lanternfish	–	–	–	–	–	–	–	–	–	1	–	–
*Gymnoscopelus nicholsi* Nichol’s lanternfish	–	–	–	–	–	–	–	–	–	1	–	–
Perciformes												
Channichthyidae (crocodile icefishes)	–	–	–	–	–	D	–	–	–	–	–	–
*Chaenodraco wilsoni* spiny icefish	–	–	–	–	–	–	–	–	33	–	–	–
*Champsocephalus gunnari* icefish	D	D	D	D	D	D	13	–	–	–	–	–
*Chionodraco* sp.	–	–	–	–	–	–	–	17	–	1	–	–
*Cryodraco antarcticus* long-fingered icefish	–	–	–	–	–	–	–	17	–	–	–	–
Nototheniidae												
*Dissostichus eleginoides* Chilean sea bass	–	D	D	–	–	–	–	–	–	–	–	–
*Notothenia coriiceps* black rockcod	–	–	–	–	–	–	19	–	–	–	–	–
*Patagonotothen* sp.	D	–	–	–	–	–	–	–	–	–	–	–
*Patagonotothen tessellata* black Southern cod	D	D	D	D	D	D	–	–	–	–	–	–
*Patagonotothen wiltoni*	–	D	–	–	D	–	–	–	–	–	–	–
*Paranotothenia* sp.	–	–	–	–	–	–	6	–	–	1	–	–
Scorpaeniformes												
Agonidae (alligatorfishes)	D	D	D	D	D	D	–	–	–	–	–	–
Psychrolutidae (blobfishes)												
*Psychrolutes* sp.	D	D	–	D	D	–	–	–	–	–	–	–

Data correspond to gentoo penguins *Pygoscelis papua* breeding at New Island, Falkland/Malvinas Islands, during chick guard (December) in 2013 and 2014, gentoo penguins breeding at Devils Point, Byers Peninsula, Livingston Island, South Shetland Islands, Antarctica, during chick guard (December 2016), and chinstrap penguins *Pygoscelis antarcticus* breeding at Vapour Col rookery, Deception Island, South Shetland Islands, Antarctica, during chick guard (January 2017)

Sample sizes: 1) *n* = number of DNA extractions from scat samples, and 2) in brackets: the number of successfully amplified samples. Unk.: age unknown, samples obtained at the colony. First chicks: first hatched chick. Second chicks: second hatched chick. ^a^ Samples from 2014 were not split by age group due to small sample sizes in most of the know age categories (see Additional file 1, Table S6). Samples from New Island are pooled (see Additional file 1, Additional Methods, Molecular analysis of the diet) and thus, frequency of occurrence cannot be calculated. Instead prey species detected are denoted with ‘D’. Best blast results for each detected taxa and corresponding accession number, the identity with the blast reference sequence, the sequence length and the bitscore are provided in Additional file 1, Table S10

### Stable isotope analysis of the diet

Mean isotope values differed among the Antarctic penguin groups (Kruskal Wallis ANOVA for δ^13^C: χ^2^ = 35.1, d.f. = 5, *P* < 0.001, for δ^15^N: χ^2^ = 46.9, d.f. = 5, *P* < 0.001; Fig. 5c, Additional file 1, Table S11). In gentoos, the differences in δ^13^C signature were related to higher values in adult males than in chicks (Fig. 5c, Additional file 1, Table S11), indicating a more benthic diet for adults, as also shown by the analyses of dive parameters in Table 2. Gentoos had also significantly higher δ^13^C than chinstraps (Fig. 5c, Additional file 1, Table S11), indicating again a more benthic diet for gentoos, in line with the significant differences in dive parameters (Table 2). In the case of δ^15^N, the differences among the groups were related to higher values in chinstrap adults than in their chicks (Fig. 5c, Additional file 1, Table S11), which is in line with the observation that chinstrap chicks were only fed with Euphausiacea (Table 3; Additional file 1, Fig. S26). All niche metrics (Fig. 5c, Additional file 1, Table S11) were larger in gentoos than in chinstraps indicating a higher variability in the feeding ecology among gentoos, as also suggested by the detected prey and their frequency of occurrence (Table 3) and the diet composition obtained using NMDS (Additional file 1, Fig. S23). Within species, gentoo males and chicks had larger niche metrics than females, while no differences were observed between first and second chicks, and between chinstrap adults and chicks (Additional file 1, Table S11). Additionally, niche metrics from the gentoos from Antarctica (Additional file 1, Table S11) were mostly larger than those from the previously studied gentoos from New Island, except in the case of New Island, South during December 2013, to which they were similar (Additional file 1, Table S12; Masello et al. 2017).

### Condition parameters

Comparisons of the H/L ratios revealed no differences between gentoos from New Island (sub-Antarctic; mean 2.7 ± 1.4) and those from Livingston (Antarctica, 2.8 ± 1.3; *t* = 0.213, *d.f.* = 20.4, *P* = 0.834). However, the mean H/L ratios of the gentoos from Livingston (2.8 ± 1.3) were higher than those measured in chinstraps (1.3 ± 0.7; Wilcoxon rank sum test with continuity correction, W = 47, *P* < 0.001; Additional file 1, Fig. S27).

## Discussion

**Fig. 6:**
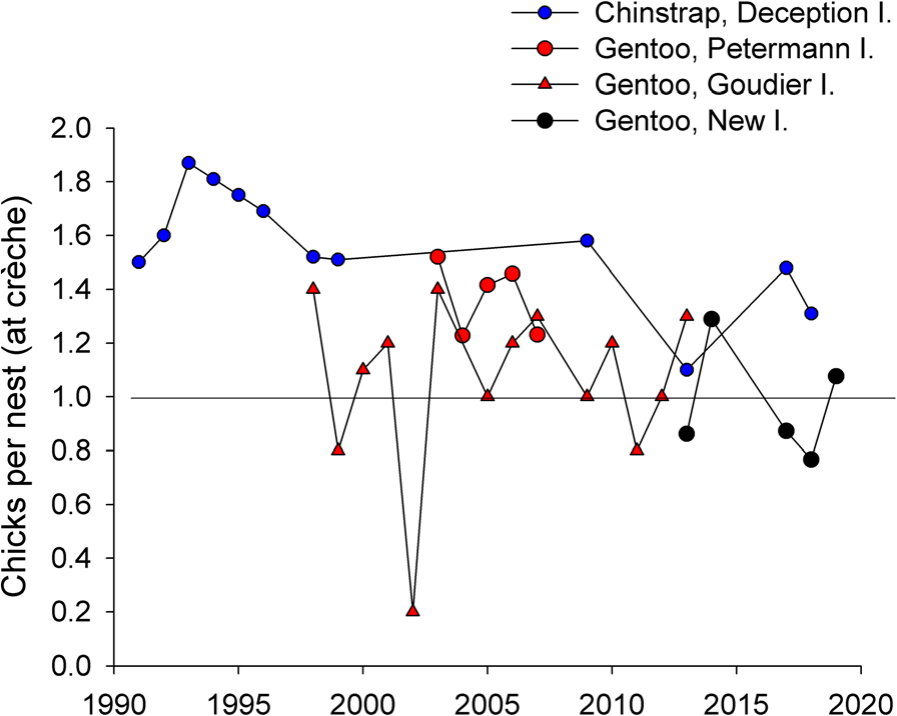
Gentoo and chinstrap penguin breeding success. Data correspond to the number of chicks per nest recorded at crèche for the chinstrap penguins *Pygoscelis antarcticus* breeding at Vapour Col rookery, Deception Island, South Shetland Islands, gentoo penguins *Pygoscelis papua* from New Island, Falkland/Malvinas Islands, and Petermann Island [109] and Goudier Island [110] in the West Antarctic Peninsula. The horizontal black line marks the value of one (1) chick per nest

Using the energy landscape paradigm (sensu Wilson et al. [23]), we tested hypotheses on the energetic balance between costs and benefits, the foraging areas selected, and the differences between different populations and different species. As predicted by our first hypothesis (a), we showed that gentoos from Livingston (Antarctica, ‘optimal’ site, positive population trends) foraged in sectors of the energy landscape where low foraging costs relative to energy gain were required (up to 137.6 J kg^− 1^ s^− 1^, Fig. 3a). Also, as predicted in our hypothesis comparing different gentoo populations (b), we found that the birds breeding at New Island (Falkland/Malvinas Islands, ‘suboptimal’ site, fluctuating populations) [32] were forced to forage in more expensive sectors of the energy landscape during poor conditions (Fig. 4d, Additional file 1, Figs. S20-S22) than those from Livingston. In the year of poor prey availability at New Island (2013) [21], the median foraging costs relative to energy gain during foraging was 167.1 J kg^− 1^ s^− 1^ and reached values up to 232, due to very long trips (90–160 km) and deeper diving (up to 188 m; Tables 1 and 2). During high prey availability at New Island (2014) [21], intermediate values of foraging costs relative to energy gain during foraging (112.7 J kg^− 1^ s^− 1^) were observed, and were comparable to those in gentoos from Livingston (115.2 J kg^− 1^ s^− 1^). The maximum foraging costs relative to energy gain at Livingston were merely 56% of those observed at New Island. Moreover, gentoos from Livingston foraged over shorter distances, closer to the colony, for less time, and dived less deep for shorter times than those from New Island (Tables 1 and 2). In a previous study [21], we showed that when the energy landscape was characterized by lower foraging costs per energy gain (2014) the breeding success was high (1.29 chicks per nest), while during a year of high foraging costs (2013) breeding success was low (0.86; see also Additional file 1, Table S13). Moreover, published records of breeding success in gentoos show a remarkable pattern: on the West Antarctic Peninsula 84% of records (16 of 19) fall above the mark of one (1) chick per nest, while on New Island this happens only in 40% (2 of 5) of the years, suggesting a generally better breeding success for Antarctic gentoos, irrespective of naturally occurring inter-annual oscillations (Fig. 6) [21, 109, 110]. All things considered, the above results are in line with our previous findings linking energy gain and variable energy costs of foraging to breeding success [21], and suggest that the lower foraging costs incurred by the gentoos from the Antarctic Peninsula, could favour a higher breeding success that, in turn, would explain the positive population trend of the species in the region, offering a plausible link between energy requirements and population dynamics (see also [20] and references therein).

A recent study [112] found that gentoos at sub-Antarctic Marion Island alternated trips of relatively short and long durations, with short trips likely associated to self-maintenance and longer trips associated to chick provisioning. Carpenter-Kling et al. [112] postulated that gentoos may be using this strategy of alternate short and long trips as a result of suboptimal feeding conditions related to environmental change. However, we did not find this behaviour at New Island, a ‘suboptimal’ site (Masello et al. 2010, 2017), while we found that gentoos performed short (20 to 33 km) and long trips (59 to 71 km) at Livingston, an ‘optimal’ site (Tables 1 and 2, Fig. 3a). Median dive depths and numbers of dives per trip were comparable (Additional file 1, Fig. S28) and thus, the swim distance had a major influence on the foraging costs relative to energy gain. An alternative explanation for this foraging behaviour could be that it allows gentoos to use the areas of the energy landscape that resulted in lower foraging costs, which show a bimodal distribution around the colony at Livingston (green areas in Fig. 3a). Also, in this case, the energy landscape paradigm offers a plausible explanation for a particular animal behaviour.

Our results do not support our hypothesis that chinstraps should show higher energy expenditure than Antarctic gentoos (between-species differences in energy expenditure, hypothesis d). Contrary to our expectations based on long-term population trends, chinstraps actually incurred lower foraging costs per bottom time gain than the gentoos from Livingston (Figs. 3a, b and 4 a-c). However, our results suggest also, in this case, a link between energy landscapes and breeding success: 1.48 chicks per nest reached the crèche stage in chinstraps at Deception during the studied season (AB unpubl. data), and this value was higher than most records for gentoos in the region (Fig. 6). The lower foraging costs per energy gain experienced by the chinstraps from Deception during this study could have allowed them to achieve a higher breeding success regardless of the long-term population trend [109], but see [113]; suggesting that the driver of population decline in this species does not operate during the breeding season [43].

Another pattern arose from the foraging behaviour of Deception chinstraps. At that island, chinstraps breed in eight different colonies, where up to 62,500 pairs breed (Additional file 1, Fig. S18) [44, 104]. All but one of the trips performed by chinstraps from Vapour Col rookery occurred outside the ‘hinterland’ (sensu Cairns [111]) of the other colonies of this species located on Deception, suggesting a potential avoidance of those areas (Additional file 1, Fig. S18). Lima & Estay [40] found that the population dynamics of chinstraps at the nearby King George Island is particularly regulated by intra-specific competition and the combined effects of Antarctic krill abundance and sea-ice cover. Our results appear to support this view. As previously found [65, 114–118], both our molecular and isotopic analyses showed, that chinstraps, particularly their chicks, had a more restricted diet than gentoos (Tables 3 and 4; Figs. 5a, c, Additional file 1, Fig. S23). Thus, in the case of chinstraps, intra-specific competition, the narrow foraging niche (Fig. 5c), and declining Antarctic krill abundances (Additional file 1, Fig. S29) [38] could explain the population trends of the species [35, 44].

**Table 4:** Diet and isotopic niche metrics

	Gentoo female adults	Gentoo male adults	Gentoo first chicks	Gentoo second chicks	Chinstrap adults	Chinstrap chicks
N	10	15	24	24	6	11
δ^13^C	-25.12±0.49^a,b^	-24.84±0.40 ^a^	-25.43±0.54 ^b,c^	-25.48±0.53^b,c^	-25.98±0.15 ^c,d^	-26.56±0.09 ^d^
δ^15^N	8.26±0.41^a^	8.48±0.65 ^a^	8.13±0.64 ^a^	8.15±0.66 ^a^	7.98±0.10 ^a,b^	7.36±0.17 ^b^
SEA	0.48	0.74	0.72	0.75	0.04	0.05
SEAc	0.55	0.80	0.75	0.78	0.05	0.06
SEAb	0.51	0.72	0.74	0.77	0.04	0.05
NR	1.65	2.45	2.24	2.64	0.31	0.58
CR	1.48	1.62	1.71	2.00	0.39	0.34
CD	0.55	0.62	0.70	0.70	0.17	0.17
NND	0.33	0.29	0.16	0.20	0.12	0.10

Data correspond to gentoo penguins *Pygoscelis papua* breeding at Devils Point, Byers Peninsula, Livingston Island, South Shetland Islands, Antarctica, during chick guard (December 2016), and chinstrap penguins *Pygoscelis antarcticus* breeding at Vapour Col rookery, Deception Island, South Shetland Islands, Antarctica, during chick guard (January 2017)

Dunn tests were used for pairwise comparisons, and homogenous subsets are marked with superscript letters. SEA: area of the standard ellipse (isotope niche width). SEAc: as SEA, corrected for sample size. SEAb: Bayesian standard ellipse area. NR: trophic length (range in δ^15^N). CR: diversity of basal resources (range in δ^13^C). CD: niche width 2 (mean distance to centroid). NND: mean nearest neighbour distance

In a previous study on gentoos [21], we showed the importance of nonlethal effects of predation as a way of better understanding animal movement. Several species of pinnipeds prey on penguins [119, 120], including Antarctic fur seals *Arctocephalus gazella*, Weddell seals *Leptonychotes weddellii* and leopard seal *Hydrurga leptonyx*. Antarctic fur seals have been observed patrolling the gentoo colony at Livingston, Weddell Seals have been found in the vicinity of this colony, and leopard seals successfully predated chinstraps during our fieldwork (JFM, AB pers. observ.). Furthermore, Antarctic fur seals breed in a large colony located close to the studied gentoo colony at Livingston (Additional file 1, Fig. S18). Still, in this study, we were not able to detect noticeable signs of active avoidance of particular areas in relationship to potential predators, as we previously observed for the gentoos from New Island [21, 51]. One explanation for this could be related to another large gentoo colony located at Barclay Bay, halfway between our studied colony and the large Antarctic fur seal colony on Cape Shirreff and San Telmo Islets, Livingston (Additional file 1, Fig. S18) [45, 105]. The foraging trips performed by gentoos from Livingston could have avoided the ‘hinterland’ of their conspecific large colony at Barclay Bay (and potentially three chinstrap colonies also present in this location), avoiding at the same time the Antarctic fur seal colony located further to the east (Additional file 1, Fig. S18). However, foraging data from the gentoos at Barclay Bay would be needed to ascertain this interpretation.

Current environmental changes in the Antarctic Peninsula [28] could affect animal physiological condition [99]. But, individuals may buffer challenging external conditions by behaviourally adjusting their exposure to costs and supplies of energy [20]. Moreover, Grémillet et al. [22] demonstrated in Adélie penguins *Pygoscelis adeliae* a relationship between individual condition and the rates of energy expenditure. As challenging environmental conditions could lead to greater energy expenditure and reduced individual condition [21, 98], and a link between energetics, individual condition and fitness has long been postulated (reviewed in [22]), we investigated physiological condition in our deployed penguins. We expected that foraging in areas of the energy landscapes with low energetic costs would lead to better individual condition (hypothesis c). Concerning populations, we found no differences in H/L ratios between gentoos from Livingston (‘optimal’ site) and New Island (‘suboptimal’), however, this probably happened because the samples from New Island were taken during the favourable conditions of 2014 (see also Additional file 1, Table S13) [21]. Unfortunately, samples from New Island during the unfavourable year 2013 were not available, leaving this a matter for future investigations. But, in support of our hypothesis, the H/L ratios of the chinstraps were lower than those measured in gentoos from Livingston (Additional file 1, Fig. S27), suggesting that foraging in areas of the energy landscapes that result in minimised energetic costs (Figs. 3b, 4c) could lead to lower physiological stress and better individual condition, which could help to achieve a higher breeding success, as in chinstraps during our study (Fig. 6). Higher H/L ratios values in gentoos compared to chinstraps have been found in several populations of both species along the Antarctic Peninsula [47, 121] supporting our interpretation of our results. However, other factors that can affect H/L ratios such as the presence of parasites and/or pathogens could also explain differences between the species.

To make the current study fully comparable with our previous work on penguins [21], we followed the approach of Wilson et al. [23] to energy landscapes. However, more recently, the definition of the energy landscape has expanded to include the effect of media flow on movement costs [12, 122]. Water current flow could have an effect on the energetic cost of penguins commuting to and from the foraging sites and should not be overlooked in future research.

## Conclusion

We applied energy landscapes to compare the foraging of penguins from colonies with different population trends. At all sites, penguins foraged in sectors of the energy landscape where low energy was required. However, when conditions were poorer, the birds were forced to forage in more expensive parts of the energy landscape. Our results also showed that lower foraging costs may favour a higher breeding success that would explain the positive population trend by the gentoo penguins from Livingston, in the Antarctic Peninsula, and the higher breeding success in chinstraps from Deception compared to gentoos. Foraging in areas of the energy landscapes that result in minimised energetic costs was associated with lower physiological stress and higher breeding success. The energy landscape approach may help to improve our understanding of the relationship between energy requirements, individual condition and breeding success and thus, between animal movement and complex ecological processes.

## Supplementary Information

**Additional file 1: Table S1**. Results of a General Additive Model (GAM) investigating the sum of Overall Dynamic Body Acceleration (ODBA) during dive as a function of maximum dive depth with the intra-depth zone (IDZ; foraging dives performed by the individuals split in benthic and pelagic), as factor. Gentoo penguins *Pygoscelis papua* were breeding at Devils Point, Byers Peninsula, Livingston Island, South Shetland Islands, Antarctica, while chinstrap penguins *Pygoscelis antarcticus* were breeding at Vapour Col rookery, Deception Island, South Shetland Islands, Antarctica. Data was obtained during chick guard. **Table S2**. The relationship between the sum of Overall Dynamic Body Acceleration (ODBA) during dive and maximum dive depth for benthic and pelagic dives (based on the index of benthic diving behaviour, intra-depth zone; IDZ). Gentoo penguins *Pygoscelis papua* were breeding at Devils Point, Byers Peninsula, Livingston Island, South Shetland Islands, Antarctica, while chinstrap penguins *Pygoscelis antarcticus* were breeding at Vapour Col rookery, Deception Island, South Shetland Islands, Antarctica. Data was obtained during chick guard. **Table S3.** The relationship between the number of dives per trip and the maximum distance from the colony during a foraging trip carried out by gentoo penguin *Pygoscelis papua* breeding at Devils Point, Byers Peninsula, Livingston Island, South Shetland Islands, Antarctica (chick guard; December 2016). See also **Figure S11. Table S4**. Results of a General Additive Model (GAM) investigating the bottom time as a function of event maximum depth (maximum depth [m] reached during dive event) with the intra-depth zone (IDZ; foraging dives performed by the individuals split in benthic and pelagic), as factor. Gentoo penguins *Pygoscelis papua* were breeding at Devils Point, Byers Peninsula, Livingston Island, South Shetland Islands, Antarctica, while chinstrap penguins *Pygoscelis antarcticus* were breeding at Vapour Col rookery, Deception Island, South Shetland Islands, Antarctica. Data was obtained during chick guard. **Table S5**. The relationship between bottom time and event maximum depth for benthic and pelagic dives (based on the index of benthic diving behaviour, intra-depth zone; IDZ). Gentoo penguins *Pygoscelis papua* were breeding at Devils Point, Byers Peninsula, Livingston Island, South Shetland Islands, Antarctica, while chinstrap penguins *Pygoscelis antarcticus* were breeding at Vapour Col rookery, Deception Island, South Shetland Islands, Antarctica. Data was obtained during chick guard. **Table S6**. The number of DNA extractions from scat samples of gentoo penguins *Pygoscelis papua* breeding at New Island, Falkland/Malvinas Islands, during chick guard (December) in 2013 and 2014, gentoo penguins breeding at Byers Peninsula, Livingston Island, Antarctica, during chick guard (December 2016), and chinstrap penguins *Pygoscelis antarcticus* breeding at Vapour Col rookery, Deception Island, South Shetland Islands, Antarctica, during chick guard (January 2017). **Table S7**. Control samples for the molecular detection of prey in scat samples from the studied penguins. **Table S8**. List of primers used in this study for the detection of prey species in scat samples from gentoo penguins *Pygoscelis papua* and chinstrap penguins *Pygoscelis antarcticus*. **Table S9.** Comparison of total foraging costs per bottom time gain (J kg^− 1^ s^− 1^) using Kruskal-Wallis rank sum test. The groups tested correspond to gentoo penguins *Pygoscelis papua* breeding at New Island, Falkland/Malvinas Islands, during chick guard (December) in 2013 and 2014, gentoo penguins breeding at Devils Point, Byers Peninsula, Livingston Island, South Shetland Islands, Antarctica, during chick guard (December 2016), and chinstrap penguins *Pygoscelis antarcticus* breeding at Vapour Col rookery, Deception Island, South Shetland Islands, Antarctica, during chick guard (January 2017). **Table S10**. Best blast results for each detected taxa and corresponding accession number, the identity with the blast reference sequence, the sequence length and the bitscore from gentoo penguins *Pygoscelis papua* breeding at New Island, Falkland/Malvinas Islands, during chick guard (December) in 2013 and 2014, gentoo penguins breeding at Devils Point, Byers Peninsula, Livingston Island, South Shetland Islands, Antarctica, during chick guard (December 2016), and chinstrap penguins *Pygoscelis antarcticus* breeding at Vapour Col rookery, Deception Island, South Shetland Islands, Antarctica, during chick guard (January 2017). **Table S11**. Diet and isotopic niche metrics. Data correspond to gentoo penguins *Pygoscelis papua* breeding at Devils Point, Byers Peninsula, Livingston Island, South Shetland Islands, Antarctica, during chick guard (December 2016), and chinstrap penguins *Pygoscelis antarcticus* breeding at Vapour Col rookery, Deception Island, South Shetland Islands, Antarctica, during chick guard (January 2017). **Table S12**. Isotopic niche metrics of gentoo penguins *Pygoscelis papua* breeding at New Island, Falkland/Malvinas Islands, during chick guard (December) in 2013 and 2014. Parameters are based on carbon (δ^13^C) and nitrogen (δ^15^N) stable isotopes of chick feather samples as a marker of breeding season foraging ecology from two colonies at New Island and two breeding seasons calculated with the SIAR package. SE: South End colony. NE: North End colony. For further details see Masello et al. (2017). **Table S13**. Gentoo penguins *Pygoscelis papua* breeding success at New Island, Falkland/Malvinas Islands. The number of chicks corresponds to crèche stage. For further details on the colonies see Masello et al. (2010, 2017). **Figure S1**. The distribution of dive depth data during benthic (A) and pelagic (B) foraging dives by gentoo penguin *Pygoscelis papua* breeding at Devils Point, Byers Peninsula, Livingston Island, South Shetland Islands, Antarctica, during chick guard (December 2016). Benthic and pelagic dives are defined with the use of the index of benthic diving behaviour, intra-depth zone (IDZ). **Figure S2.** The distribution of dive depth data during benthic (A) and pelagic (B) foraging dives by chinstrap penguins *Pygoscelis antarcticus* breeding at Vapour Col rookery, Deception Island, South Shetland Islands, Antarctica, during chick guard (January 2017). Benthic and pelagic dives are defined with the use of the index of benthic diving behaviour, intra-depth zone (IDZ). **Figure S3.** Example of the distribution in different depths of benthic (A) and pelagic (B) dives carried out by gentoo penguin *Pygoscelis papua* breeding at Devils Point, Byers Peninsula, Livingston Island, South Shetland Islands, Antarctica, during chick guard (December 2016). Depth (in m) is based on data from the International Bathymetric Chart of the Southern Ocean (IBCSO; Arndt et al. 2013). **Figure S4.** Example of the distribution in different depths of benthic (A) and pelagic (B) dives carried out by chinstrap penguins *Pygoscelis antarcticus* breeding at Vapour Col rookery, Deception Island, South Shetland Islands, Antarctica, during chick guard (January 2017). Depth (in m) is based on data from the International Bathymetric Chart of the Southern Ocean (IBCSO; Arndt et al. 2013). **Figure S5**. Foraging trips by female (red burgundy) and male (yellow) gentoo penguins *Pygoscelis papua* breeding at Devils Point, Byers Peninsula, Livingston Island, South Shetland Islands, Antarctica, during chick guard (December 2016) (A), and chinstrap penguins *Pygoscelis antarcticus* breeding at Vapour Col rookery, Deception Island, South Shetland Islands, Antarctica, during chick guard (January 2017) (B). Depth (m) is based on data from the International Bathymetric Chart of the Southern Ocean (IBCSO; Arndt et al. 2013). **Figure S6**. Example of dive profiles and tri-axial acceleration data during three consecutive dives by gentoo penguins *Pygoscelis papua* breeding at Devils Point, Byers Peninsula, Livingston Island, South Shetland Islands, Antarctica, during chick guard (December 2016). Acceleration data correspond to each of the three spatial axes: x, surge (green), y, heave (red), and z, sway (blue). Dive depth is given in metres and 0 (zero) corresponds to the water surface. **Figure S7.** The distribution of foraging parameter data used for the calculations of energy landscapes in gentoo penguins *Pygoscelis papua* breeding at Devils Point, Byers Peninsula, Livingston Island, South Shetland Islands, Antarctica, during chick guard (December 2016). See also Table 1. **Figure S8.** The distribution of foraging parameter data used for the calculations of energy landscapes in chinstrap penguins *Pygoscelis antarcticus* breeding at Vapour Col rookery, Deception Island, South Shetland Islands, Antarctica, during chick guard (January 2017). See also Table 1. **Figure S9.** The relationship between the sum of Overall Dynamic Body Acceleration (ODBA) during dive and maximum dive depth for benthic dives (based on the index of benthic diving behaviour, intra-depth zone; IDZ) carried out by gentoo penguin *Pygoscelis papua* breeding at Devils Point, Byers Peninsula, Livingston Island, Antarctica, during chick guard (December 2016). Details for the regression curve are given in **Table S2**. **Figure S10.** The relationship between the sum of Overall Dynamic Body Acceleration (ODBA) during dive and maximum dive depth for pelagic dives (based on the index of benthic diving behaviour, intra-depth zone; IDZ) carried out by gentoo penguin *Pygoscelis papua* breeding at Byers Peninsula, Livingston Island, Antarctica during chick guard (December 2016). Details for the regression curve are given in **Table S2**. **Figure S11.** The relationship between the sum of Overall Dynamic Body Acceleration (ODBA) during dive and maximum dive depth for benthic dives (based on the index of benthic diving behaviour, intra-depth zone; IDZ) carried out by chinstrap penguins *Pygoscelis antarcticus* breeding at Vapour Col rookery, Deception Island, South Shetland Islands, Antarctica, during chick guard (January 2017). Details for the regression curve are given in **Table S2**. **Figure S12.** The relationship between the sum of Overall Dynamic Body Acceleration (ODBA) during dive and maximum dive depth for pelagic dives (based on the index of benthic diving behaviour, intra-depth zone; IDZ) carried out by chinstrap penguins *Pygoscelis antarcticus* breeding at Vapour Col rookery, Deception Island, South Shetland Islands, Antarctica, during chick guard (January 2017). Details for the regression curve are given in **Table S2**. **Figure S13.** The relationship between the number of dives per trip and the maximum distance from the colony during a foraging trip carried out by gentoo penguin *Pygoscelis papua* breeding at Byers Peninsula, Livingston Island, Antarctica (chick guard; December 2016). Details for the regression curve are given in **Table S3**. **Figure S14.** The relationship between the bottom time and the event maximum depth for benthic dives (based on the index of benthic diving behaviour, intra-depth zone; IDZ) carried out by gentoo penguin *Pygoscelis papua* breeding at Byers Peninsula, Livingston Island, Antarctica, during chick guard (December 2016). Details for the regression curve are given in **Table S4**. **Figure S15.** The relationship between the bottom time and the event maximum depth for pelagic dives (based on the index of benthic diving behaviour, intra-depth zone; IDZ) carried out by gentoo penguin *Pygoscelis papua* breeding at Byers Peninsula, Livingston Island, Antarctica, during chick guard (December 2016). Details for the regression curve are given in **Table S4**. **Figure S16.** The relationship between the bottom time and the event maximum depth for benthic dives (based on the index of benthic diving behaviour, intra-depth zone; IDZ) carried out by chinstrap penguins *Pygoscelis antarcticus* breeding at Vapour Col rookery, Deception Island, South Shetland Islands, Antarctica, during chick guard (January 2017). Details for the regression curve are given in **Table S4**. **Figure S17**. The relationship between the bottom time and the event maximum depth for pelagic dives (based on the index of benthic diving behaviour, intra-depth zone; IDZ) carried out by chinstrap penguins *Pygoscelis antarcticus* breeding at Vapour Col rookery, Deception Island, South Shetland Islands, Antarctica, during chick guard (January 2017). Details for the regression curve are given in **Table S4**. **Figure S18**. Location and size of other colonies of gentoo penguins *Pygoscelis papua* (degrees of red dots), chinstrap penguins *Pygoscelis antarcticus* (degrees of blue dots), and Antarctic Fur Seals *Arctocephalus gazella* (degrees of brown dots) in the South Shetland Islands, Antarctica. The locations and size of the Fur Seal colonies were obtained from Hucke-Gaete et al. (2004), while those from the penguin colonies were obtained from Naveen et al. (2000) and the Mapping Application for Penguin Populations and Projected Dynamics (MAPPPD) (Humphries et al. 2017) available at http://www.penguinmap.com . Size for penguins: pairs. Size for fur seals: individuals. Foraging trip coded as in Fig. 2a. **Figure S19**. Kernel density distribution of dive locations and bathymetry. The 50% core areas are denoted by black lines, while 95% home ranges by yellow lines. Kernel density distributions represent the places where the penguins spent most of their forging time. Data from gentoo penguins *Pygoscelis papua* breeding at Devils Point, Byers Peninsula, Livingston Island, South Shetland Islands, Antarctica, during chick guard (December 2016), is further coded in short (dashed lines) and long trips (solid lines). Data from chinstrap penguins *Pygoscelis antarcticus* breeding at Vapour Col rookery, Deception Island, South Shetland Islands, Antarctica, during chick guard (January 2017) is denoted by solid lines only, as no distinction between short and long trips could be found. Depth (in m) is based on data from the International Bathymetric Chart of the Southern Ocean (IBCSO; Arndt et al. 2013). **Figure S20**. Energy landscapes based on the bathymetry around New Island, Falkland/Malvinas Islands, and the mass-specific total cost of foraging (diving plus commuting) by gentoo penguins *Pygoscelis papua* relative to the bottom time (in J kg^− 1^ s^− 1^), considering the different proportion of benthic and pelagic dives carried out by the penguins from the South End colony during the 2013 breeding season. For further details see Masello et al. (2017). **Figure S21**. Energy landscapes based on the bathymetry around New Island, Falkland/Malvinas Islands, and the mass-specific total cost of foraging (diving plus commuting) by gentoo penguins *Pygoscelis papua* relative to the bottom time (in J kg^− 1^ s^− 1^), considering the different proportion of benthic and pelagic dives carried out by the penguins from the South End colony during the 2014 breeding season. For further details see Masello et al. (2017). **Figure S22**. Energy landscapes based on the bathymetry around New Island, Falkland/Malvinas Islands, and the mass-specific total cost of foraging (diving plus commuting) by gentoo penguins *Pygoscelis papua* relative to the bottom time (in J kg^− 1^ s^− 1^), considering the different proportion of benthic and pelagic dives carried out by the penguins from the North End colony during the 2014 breeding season. For further details see Masello et al. (2017). **Figure S23**. Diet composition using non-metric multidimensional scaling (NMDS) of molecular operational taxonomic units (MOTUs). Data corresponds to gentoo penguins *Pygoscelis papua* at Devils Point, Byers Peninsula, Livingston Island, South Shetland Islands, Antarctica, during chick guard (Dec 2016), and chinstrap penguins *Pygoscelis antarcticus* at Vapour Col rookery, Deception Island, South Shetland Islands, Antarctica, during chick guard (Jan 2017). A) includes the identity of the prey consumed, while B) the ellipses and C) the convex hulls connecting similar categories. Gentoo: black dots and lines, and dark grey shade. Chinstrap: royal blue dots and lines, and royal blue shade. **Figure S24.** Diet composition using non-metric multidimensional scaling (NMDS) of molecular operational taxonomic units (MOTUs). Data corresponds to gentoo penguins *Pygoscelis papua* at Devils Point, Byers Peninsula, Livingston Island, South Shetland Islands, Antarctica, during chick guard (Dec 2016), and chinstrap penguins *Pygoscelis antarcticus* at Vapour Col rookery, Deception Island, South Shetland Islands, Antarctica, during chick guard (Jan 2017). A) includes the identity of the prey consumed, while B) the ellipses and C) the convex hulls connecting similar categories. The categories included are gentoo adults (back and dark grey), gentoo chicks (red), chinstrap adults (royal blue), and chinstrap chicks (cyan; not visible A) and C). **Figure S25.** Diet composition using non-metric multidimensional scaling (NMDS) of molecular operational taxonomic units (MOTUs). Data corresponds to gentoo penguins *Pygoscelis papua* at Devils Point, Byers Peninsula, Livingston Island, South Shetland Islands, Antarctica, during chick guard (Dec 2016). A) includes the identity of the prey consumed, while B) the ellipses and C) the convex hulls connecting similar categories. The categories included are adults (black and dark grey) and chicks (red). **Figure S26**. Diet composition using non-metric multidimensional scaling (NMDS) of molecular operational taxonomic units (MOTUs). Data corresponds to chinstrap penguins *Pygoscelis antarcticus* at Vapour Col rookery, Deception Island, South Shetland Islands, Antarctica, during chick guard (Jan 2017). A) includes the identity of the prey consumed, while B) the ellipses and C) the convex hulls connecting similar categories. The categories included are adults (royal blue) and chicks (cyan). **Figure S27**. Boxplot of the ratio of two types of leucocytes, the heterophils and lymphocytes (H/L ratio), belonging to chinstrap penguins *Pygoscelis antarcticus* at Vapour Col rookery, Deception Island, South Shetland Islands, Antarctica, during chick guard (Jan 2017), and gentoo penguins *Pygoscelis papua* at Devils Point, Byers Peninsula, Livingston Island, South Shetland Islands, Antarctica, during chick guard (Dec 2016). Boxplots include medians, whiskers indicating variability outside the upper and lower quartiles, and outliers (circles). **Figure S28**. Dive depth of pelagic dives corresponding to gentoo penguins *Pygoscelis papua* breeding at New Island (Falkland/Malvinas Islands), during chick guard (December) in 2013 and 2014, gentoo penguins breeding at Devils Point, Byers Peninsula, Livingston Island, South Shetland Islands, Antarctica, during chick guard (December 2016), and chinstrap penguins *Pygoscelis antarcticus* breeding at Vapour Col rookery, Deception Island, South Shetland Islands, Antarctica, during chick guard (January 2017). **Figure S29**. Standardised abundance of the Antarctic Krill *Euphausia superba* obtained from the KRILLBASE (Atkinson et al. 2017) for the sector between 60 and 65°S and 55–65°W (A), and Antarctic Krill catches for the CCAMLR Area 48 (B). **Additional Methods**, Molecular analysis of the diet. **Additional References**

## Data Availability

The GPS-TD and tri-axial acceleration data supporting the conclusions of this article are archived in the Movebank repository (IDs, 1108578788132, 500849549, 246072457, 246072308; https://www.movebank.org/). All other datasets supporting the conclusions of this article are included or cited within the article and its Additional file 1.

## Abbreviations

ANOVA: Analysis of variance
BBT: Sum of benthic bottom time
BM: Mean adult body mass in kg
CCAMLR: Commission for the Conservation of Antarctic Marine Living Resources
CD: Mean distance to centroid
CR: δ^13^C range
CT: Cost of travelling
DD: Mean dive duration
DD_benthic_: Mean dive duration for benthic dives
DD_pelagic_: Mean dive duration for pelagic dives
DNA: Deoxyribonucleic acid
ENSO: El Niño Southern Oscillation
FO: Frequency of occurrence
GPS-TD: Global Positioning System-temperature-depth
H/L: Heterophils and lymphocytes ratio
IBCSO: International Bathymetric Chart of the Southern Ocean
IDZ: Intra-depth zone
IDW: Inverse distance weighted interpolation
mBBT: Minimum benthic bottom times
MND: Median number of dives per foraging trip
MND_benthic_: Median number of benthic dives per foraging trip
MND_pelagic_: Median number of pelagic dives per foraging trip
MOTUs: Molecular operational taxonomic units
MP: Mass-specific power
MP_benthic_: Mass-specific power for benthic dives
MP_pelagic_: Mass-specific power for pelagic dives
NASA: National Aeronautics and Space Administration
NCBI: National Center for Biotechnology Information
NE: North End colony
NGS: Next generation sequencing
NMDS: Non-metric multidimensional scaling
NND: Mean nearest-neighbour distance
NR: δ^15^N range
ODBA: Overall Dynamic Body Acceleration
pBD: Mean proportion of benthic dives
PBT: Pelagic bottom times
PCR: Polymerase chain reaction
PERMANOVA: Permutational multivariate analysis of variance using distance matrices
pPD: Mean proportion of pelagic dives
SAC: Species accumulation curves
SE: South End colony
SEA: Standard ellipse areas
SEAb: Bayesian standard ellipse area
SEAc: Standard ellipse areas corrected for sample size
SIAR: Stable Isotope Analyses in R
SIBER: Stable Isotope Bayesian Ellipses in R
SOI: Southern Oscillation Index
TBT: Total bottom time
TCF: Total cost of foraging
TRC: Total relative cost
TT: Travel time
V_o_: Rate of oxygen consumption

## References

[1] Allen AM, Singh NJ. Linking movement ecology with wildlife management and conservation. Front Ecol Evol. 2016;3:e155. 10.3389/fevo.2015.00155.

[2] Doherty TS, Driscoll DA. Coupling movement and landscape ecology for animal conservation in production landscapes. Proc R Soc B. 2018;285:e20172272. 10.1098/rspb.2017.2272. PMC5784197 29298935

[3] Ripple WJ, Wolf C, Newsome TM, Barnard P, Moomaw WR. World scientists’ warning of a climate emergency. BioScience. 2020;70:8–12. 10.1093/biosci/biz088.

[4] Kays R, Crofoot MC, Jetz W, Wikelski M. Terrestrial animal tracking as an eye on life and planet. Science. 2015;348:aaa2478. 10.1126/science.aaa2478. 10.1126/science.aaa2478 26068858

[5] Foley JA, DeFries R, Asner GP, Barford C, Bonan G, Carpenter SR (2005). Global consequences of land use. Science..

[6] Nathan R, Getz WM, Revilla E, Holyoak M, Kadmon R, Saltz D, Smouse PE (2008). A movement ecology paradigm for unifying organismal movement research. Proc Natl Acad Sci U S A.

[7] Dugger KM, Ballard G, Ainley DG, Lyver POB, Schine C. Adélie penguins coping with environmental change: results from a natural experiment at the edge of their breeding range. Front Ecol Evol. 2014;2:art68. 10.3389/fevo.2014.00068.

[8] Hays GC, Ferreira LC, Sequeira AMM, Meekan MG, Duarte CM, Bailey H, Bailleul F, Bowen WD, Caley MJ, Costa DP, Eguíluz VM, Fossette S, Friedlaender AS, Gales N, Gleiss AC, Gunn J, Harcourt R, Hazen EL, Heithaus MR, Heupel M, Holland K, Horning M, Jonsen I, Kooyman GL, Lowe CG, Madsen PT, Marsh H, Phillips RA, Righton D, Ropert-Coudert Y, Sato K, Shaffer SA, Simpfendorfer CA, Sims DW, Skomal G, Takahashi A, Trathan PN, Wikelski M, Womble JN, Thums M (2016). Key questions in marine megafauna movement ecology. Trends Ecol Evol.

[9] López-López P. Individual-based tracking systems in ornithology: welcome to the era of big data. Ardeola. 2016;63:03–36. 10.13157/arla.63.1.2016.rp5.

[10] Augé AA, Dias MP, Lascelles B, Baylis AMM, Black A, Boersma PD, Catry P, Crofts S, Galimberti F, Granadeiro JP, Hedd A, Ludynia K, Masello JF, Montevecchi W, Phillips RA, Pütz K, Quillfeldt P, Rebstock GA, Sanvito S, Staniland IJ, Stanworth A, Thompson D, Tierney M, Trathan PN, Croxall JP (2018). Framework for mapping key areas for marine megafauna to inform marine spatial planning: the Falkland Islands case study. Mar Pol.

[11] Baylis AMM, Tierney M, Orben RA, Warwick-Evans V, Wakefield E, Grecian WJ, Trathan P, Reisinger R, Ratcliffe N, Croxall J, Campioni L, Catry P, Crofts S, Boersma PD, Galimberti F, Granadeiro JP, Handley J, Hayes S, Hedd A, Masello JF, Montevecchi WA, Pütz K, Quillfeldt P, Rebstock GA, Sanvito S, Staniland IJ, Brickle P (2019). Important at-sea areas of colonial breeding marine predators on the southern Patagonian shelf. Sci Rep.

[12] Gallagher AJ, Creel S, Wilson RP, Cooke SJ (2017). Energy landscapes and the landscape of fear. Trends Ecol Evol.

[13] MacArthur RH, Pianka ER (1966). On the optimal use of a patchy environment. Am Nat.

[14] Schoener TW (1971). Theory of feeding strategies. Annu Rev Ecol Syst.

[15] Thorpe SKS, Crompton RH, Alexander RM (2007). Orangutans use compliant branches to lower the energetic cost of locomotion. Biol Lett.

[16] Wilson RP, Shepard ELC, Liebsch N. Prying into the intimate details of animal lives: use of a daily diary on animals. Endanger Species Res. 2008;4:23–37. 10.3354/esr00064.

[17] Lempidakis E, Wilson RP, Luckman A, Metcalfe RS. What can knowledge of the energy landscape tell us about animal movement trajectories and space use? A case study with humans. J Theo Biol. 2018;57:101–11. 10.1016/j.jtbi.2018.08.024. 10.1016/j.jtbi.2018.08.024 30130547

[18] Gaillard J-M, Hebblewhite M, Loison A, Fuller M, Powell R, Basille M, van Moorter B (2010). Habitat–performance relationships: finding the right metric at a given spatial scale. Phil Trans R Soc B.

[19] Mosser AA, Avgar T, Brown GS, Walker CS, Fryxell JM (2014). Towards an energetic landscape: broad-scale accelerometry in woodland caribou. J Anim Ecol.

[20] Long RA, Bowyer RT, Porter WP, Mathewson P, Monteith KL, Findholt SL, Dick BL, Kie JG (2016). Linking habitat selection to fitness-related traits in herbivores: the role of the energy landscape. Oecologia..

[21] Masello JF, Kato A, Sommerfeld J, Mattern T, Quillfeldt P. How animals distribute themselves in space: variable energy landscapes. Front Zool. 2017;14:art33. 10.1186/s12983-017-0219-8. 10.1186/s12983-017-0219-8 PMC5499017 28694838

[22] Grémillet D, Lescroël A, Ballard G, Dugger KM, Massaro M, Porzig EL, Ainley DG (2018). Energetic fitness: field metabolic rates assessed via 3D accelerometry complement conventional fitness metrics. Funct Ecol.

[23] Wilson RP, Quintana F, Hobson VJ (2012). Construction of energy landscapes can clarify the movement and distribution of foraging animals. Proc R Soc B.

[24] Shepard ELC, Wilson RP, Rees WG, Grundy E, Lambertucci SA, Simon BV (2013). Energy landscapes shape animal movement ecology. Am Nat.

[25] Wall J, Douglas-Hamilton I, Vollrath F (2006). Elephants avoid costly mountaineering. Curr Biol.

[26] Brownscombe JW, Gutowsky LF, Danylchuk AJ, Cooke SJ (2014). Foraging behaviour and activity of a marine benthivorous fish estimated using tri-axial accelerometer biologgers. Mar Ecol Prog Ser.

[27] Amélineau F, Fort J, Mathewson P, Speirs D, Courbin N, Perret S (2018). Energyscapes and prey fields shape a North Atlantic seabird wintering hotspot under climate change. R Soc Open Sci.

[28] Convey P, Peck LS. Antarctic environmental change and biological responses. Sci Adv. 2019;5:eaaz0888. 10.1126/sciadv.aaz0888. 10.1126/sciadv.aaz0888 PMC6881164 31807713

[29] Hinke JT, Salwicka K, Trivelpiece SG, Watters GM, Trivelpiece WZ (2007). Divergent responses of *Pygoscelis* penguins reveal a common environmental driver. Oecologia..

[30] Handley JM, Baylis AM, Brickle P, Pistorius P (2016). Temporal variation in the diet of gentoo penguins at the Falkland Islands. Polar Biol.

[31] Miller AK, Karnovsky NJ, Trivelpiece WZ (2009). Flexible foraging strategies of gentoo penguins *Pygoscelis papua* over 5 years in the South Shetland Islands, Antarctica. Mar Biol.

[32] Baylis AMM, Zuur AF, Brickle P, Pistorius PA (2012). Climate as a driver of population variability in breeding Gentoo penguins *Pygoscelis papua* at the Falkland Islands. Ibis..

[33] Lynch HJ, Naveen R, Fagan WF (2008). Censuses of penguin, blue-eyed shag *Phalacrocorax atriceps* and southern giant petrel *Macronectes giganteus* populations on the Antarctic Peninsula, 2001–2007. Mar Ornithol.

[34] Schofield O, Ducklow HW, Martinson DG, Meredith MP, Moline MA, Fraser WR (2010). How do polar marine ecosystems respond to rapid climate change?. Science..

[35] Clucas GV, Dunn MJ, Dyke G, Emslie SD, Naveen R, Polito MJ, et al. A reversal of fortunes: climate change ‘winners’ and ‘losers’ in Antarctic peninsula penguins. Sci Rep. 2014;4:5024. 10.1038/srep05024. 10.1038/srep05024 PMC4034736 24865774

[36] Fraser WR, Trivelpiece WZ, Ainley DG, Trivelpiece SG. Increases in Antarctic penguin populations: reduced competition with whales or a loss of sea ice due to environmental warming? Polar Biol. 1992;11:525–31. 10.1007/BF00237945.

[37] Ainley D, Ballard G, Ackley S, Blight LK, Eastman JT, Emslie SD, Lescroël A, Olmastroni S, Townsend SE, Tynan CT, Wilson P, Woehler E (2007). Paradigm lost, or is top-down forcing no longer significant in the Antarctic marine ecosystem?. Antarct Sci.

[38] Trivelpiece WZ, Hinke JT, Miller AK, Reiss CS, Trivelpiece SG, Watters GM (2011). Variability in krill biomass links harvesting and climate warming to penguin population changes in Antarctica. Proc Natl Acad Sci U S A.

[39] Atkinson A, Siegel V, Pakhomov E, Rothery P (2004). Long-term decline in krill stock and increase in salps within the Southern Ocean. Nature..

[40] Lima M, Estay S (2013). Warming effects in the western Antarctic Peninsula ecosystem: the role of population dynamic models for explaining and predicting penguin trends. Popul Ecol.

[41] Lynch HJ, Naveen R, Trathan PN, Fagan WF (2012). Spatially integrated assessment reveals widespread changes in penguin populations on the Antarctic Peninsula. Ecology..

[42] McMahon KW, Michelson CI, Hart T, McCarthy MD, Patterson WP, Polito MJ (2019). Divergent trophic responses of sympatric penguin species to historic anthropogenic exploitation and recent climate change. Proc Natl Acad Sci U S A.

[43] Barbosa A, Benzal J, De León A, Moreno J (2012). Population decline of chinstrap penguins (*Pygoscelis antarctica*) on Deception Island, South Shetlands, Antarctica. Polar Biol.

[44] Naveen R, Lynch HJ, Forrest S, Mueller T, Polito M. First direct, site-wide penguin survey at Deception Island, Antarctica, suggests significant declines in breeding chinstrap penguins. Polar Biol. 2012;35:1879–88. 10.1007/s00300-012-1230-3.

[45] Gil-Delgado JA, González-Solís J, Barbosa A. Populations of breeding birds in Byers Peninsula, Livingston Island, South Shetland Islands. Antarct Sci. 2013;25(Special Issue 02):303–6. 10.1017/S0954102012000752.

[46] Strange I, Catry P, Strange G, Quillfeldt P. New Island, Falkland Islands. A South Atlantic wildlife sanctuary for conservation management: New Island Conservation Trust; 2007.

[47] Barbosa A, Merino S, Benzal J, Martinez J, García-Fraile S. Geographic variation in the immunoglobulin levels in pygoscelid penguins. Polar Biol. 2007;30:219–25. 10.1007/s00300-006-0175-9.

[48] Bannasch R, Wilson R, Culik B (1994). Hydrodynamic aspects of design and attachment of a back-mounted device in penguins. J Exp Biol.

[49] Ludynia K, Dehnhard N, Poisbleau M, Demongin L, Masello JF, Quillfeldt P (2012). Evaluating the impact of handling and logger attachment on foraging parameters and physiology in southern Rockhopper penguins. PLoS One.

[50] Griffiths R, Double M, Orr K, Dawson R (1998). A DNA test to sex most birds. Mol Ecol.

[51] Masello JF, Mundry R, Poisbleau M, Demongin L, Voigt CC, Wikelski M, et al. Diving seabirds share foraging space and time within and among species. Ecosphere. 2010;1:art19. 10.1890/ES10-00103.1.

[52] Palacios MJ, Valera F, Colominas-Ciuró R, Barbosa A (2018). Cellular and humoral immunity in two highly demanding energetic life stages: reproduction and moulting in the chinstrap penguin. J Ornithol.

[53] Arndt JE, Schenke HW, Jakobsson M, Nitsche FO, Buys G, Goleb YB, et al. The International Bathymetric Chart of the Southern Ocean (IBCSO) Version 1.0—a new bathymetric compilation covering circum-Antarctic waters. Geophys Res Lett. 2013;40:3111–7. 10.1002/grl.50413.

[54] Smith WH, Sandwell DT (1997). Global Sea floor topography from satellite altimetry and ship depth soundings. Science..

[55] Mattern T, Ellenberg U, Houston DM, Davis LS (2007). Consistent foraging routes and benthic foraging behaviour in yellow-eyed penguins. Mar Ecol Prog Ser.

[56] Tremblay Y, Cherel Y (2000). Benthic and pelagic dives: a new foraging behaviour in rockhopper penguins. Mar Ecol Prog Ser.

[57] Wood AG, Naef-Daenzer B, Prince PA, Croxall JP (2000). Quantifying habitat use in satellite-tracked pelagic seabirds: application of kernel estimation to albatross location. J Avian Biol.

[58] Laver PN, Kelly MJ (2008). A critical review of home range studies. J Wildl Manag.

[59] Dinno A. dunn.test: Dunn’s test of multiple comparisons using rank sums. R package version 1.3.5. 2017. https://cran.r-project.org/web/packages/dunn.test/index.html. Accessed 28 Oct 2020.

[60] Gleiss AC, Wilson RP, Shepard EL (2011). Making overall dynamic body acceleration work: on the theory of acceleration as a proxy for energy expenditure. Methods Ecol Evol.

[61] Halsey LG, Shepard ELC, Quintana F, Gómez Laich A, Green JA, Wilson RP (2009). The relationship between oxygen consumption and body acceleration in a range of species. Comp Biochem Physiol Part A.

[62] Wilson RP, Börger L, Holton MD, Scantlebury DM, Gómez-Laich A, Quintana F, Rosell F, Graf PM, Williams H, Gunner R, Hopkins L, Marks N, Geraldi NR, Duarte CM, Scott R, Strano MS, Robotka H, Eizaguirre C, Fahlman A, Shepard ELC (2020). Estimates for energy expenditure in free-living animals using acceleration proxies: a reappraisal. J Anim Ecol.

[63] Quintana F, Wilson RP, Yorio P (2007). Dive depth and plumage air in wettable birds: the extraordinary case of the imperial cormorant. Mar Ecol Prog Ser.

[64] Shepard ELC, Wilson RP, Quintana F, Gómez Laich A, Forman DW (2009). Pushed for time or saving on fuel: fine-scale energy budgets shed light on currencies in a diving bird. Proc R Soc B.

[65] Rombolá EF, Marschoff E, Coria N (2010). Inter-annual variability in chinstrap penguin diet at South Shetland and South Orkneys Islands. Polar Biol.

[66] Hastie T. gam: Generalized additive models. R package version 1.16.1. 2019. https://CRAN.R-project.org/package=gam. Accessed 28 Oct 2020.

[67] Lowther AD, Trathan P, Tarroux A, Lydersen C, Kovacs KM (2018). The relationship between coastal weather and foraging behaviour of chinstrap penguins, *Pygoscelis antarctica*. ICES J Mar Sci.

[68] Culik B, Wilson R, Dannfeld R, Adelung D, Spairani H, Coria NRC. Pygoscelid penguins in a swim canal. Polar Biol. 1991;11:277–82. 10.1007/BF00238463.

[69] Davis RW, Croxall JP, O'Connell MJ (1989). The reproductive energetics of Gentoo (*Pygoscelis papua*) and macaroni (*Eudyptes chrysolophus*) penguins at South Georgia. J Anim Ecol.

[70] Wilson RP, White CR, Quintana F, Halsey LG, Liebsch N, Martin GR, Butler PJ (2006). Moving towards acceleration for estimates of activity-specific metabolic rate in free-living animals: the case of the cormorant. J Anim Ecol.

[71] Elliott KH (2016). Measurement of flying and diving metabolic rate in wild animals: review and recommendations. Comp Biochem Physiol Part A.

[72] Heldmaier G, Neuweiler G, Rössler W. Vergleichende Tierphysiologie: Springer; 2013.

[73] Takahashi A, Dunn M, Trathan P, Croxall J, Wilson RP, Sato K, Naito Y (2004). Krill-feeding behaviour in a chinstrap penguin compared to fish-eating in Magellanic penguins: a pilot study. Mar Ornithol.

[74] Bost CA, Handrich Y, Butler PJ, Fahlman A, Halsey LG, Woakes AJ, Ropert-Coudert Y (2007). Changes in dive profiles as an indicator of feeding success in king and Adelie penguins. Deep-Sea Res II.

[75] Carroll G, Slip D, Jonsen I, Harcourt R (2014). Supervised accelerometry analysis can identify prey capture by penguins at sea. J Exp Biol.

[76] Pütz K, Rey AR, Huin N, Schiavini A, Pütz A, Luthi BH (2006). Diving characteristics of southern rockhopper penguins (*Eudyptes c. chrysocome*) in the Southwest Atlantic. Mar Biol.

[77] Elliott KH, Davoren GK, Gaston AJ (2008). Time allocation by a deep-diving bird reflects prey type and energy gain. Anim Behav.

[78] Johnston K, Ver Hoef JM, Krivoruchko K, Lucas N. Using ArcGIS geostatistical analyst: Esri Redlands; 2001.

[79] Bolger AM, Lohse M, Usadel B (2014). Trimmomatic: a flexible trimmer for Illumina sequence data. Bioinformatics..

[80] Magoč T, Salzberg SL (2011). FLASH: fast length adjustment of short reads to improve genome assemblies. Bioinformatics..

[81] Schloss PD, Westcott SL, Ryabin T, Hall JR, Hartmann M, Hollister EB, Lesniewski RA, Oakley BB, Parks DH, Robinson CJ, Sahl JW, Stres B, Thallinger GG, van Horn DJ, Weber CF (2009). Introducing mothur: open-source, platform-independent, community-supported software for describing and comparing microbial communities. Appl Environ Microbiol.

[82] Edgar RC (2010). Search and clustering orders of magnitude faster than BLAST. Bioinformatics..

[83] Altschul SF, Gish W, Miller W, Myers EW, Lipman DJ (1990). Basic local alignment search tool. J Mol Biol.

[84] Afgan E, Baker D, Batut B, van den Beek M, Bouvier D, Čech M, Chilton J, Clements D, Coraor N, Grüning BA, Guerler A, Hillman-Jackson J, Hiltemann S, Jalili V, Rasche H, Soranzo N, Goecks J, Taylor J, Nekrutenko A, Blankenberg D (2018). The galaxy platform for accessible, reproducible and collaborative biomedical analyses: 2018 update. Nucleic Acids Res.

[85] Deagle BE, Kirkwood R, Jarman SN (2009). Analysis of Australian fur seal diet by pyrosequencing prey DNA in faeces. Mol Ecol.

[86] Vesterinen EJ, Lilley T, Laine VN, Wahlberg N (2013). Next generation sequencing of fecal DNA reveals the dietary diversity of the widespread insectivorous predator Daubenton’s bat (*Myotis daubentonii*) in southwestern Finland. PLoS One.

[87] Kleinschmidt B, Burger C, Dorsch M, Nehls G, Heinänen S, Morkūnas J (2019). The diet of red-throated divers (*Gavia stellata*) overwintering in the German Bight (North Sea) analysed using molecular diagnostics. Mar Biol.

[88] Crisol-Martínez E, Moreno-Moyano LT, Wormington KR, Brown PH, Stanley D (2016). Using next-generation sequencing to contrast the diet and explore pest-reduction services of sympatric bird species in macadamia orchards in Australia. PLoS One.

[89] Barrett RT, Camphuysen K, Anker-Nilssen T, Chardine JW, Furness RW, Garthe S (2007). Diet studies of seabirds: a review and recommendations. ICES J Mar Sci.

[90] Oksanen J, Blanchet FG, Kindt R, Legendre P, Minchin PR, O’Hara R, et al. vegan: community ecology package. R package version 2.0–2. 2012. https://cran.r-project.org/web/packages/vegan/index.html. Accessed 28 Oct 2020.

[91] Faith DP, Minchin PR, Belbin L (1987). Compositional dissimilarity as a robust measure of ecological distance. Vegetatio..

[92] Minchin PR (1987). An evaluation of the relative robustness of techniques for ecological ordination. Vegetatio..

[93] Hobson KA, Clark R (1993). Turnover of ^13^C in cellular and plasma fractions of blood: implications for nondestructive sampling in avian dietary studies. Auk..

[94] Parnell A, Inger R, Bearhop S, Jackson AL. Stable isotope analysis in R (SIAR). 2013 http://cran.r-project.org/web/packages/siar/index.html. Accessed 28 Oct 2020.

[95] Jackson AL, Inger R, Parnell AC, Bearhop S (2011). Comparing isotopic niche widths among and within communities: SIBER – stable isotope Bayesian ellipses in R. J Anim Ecol.

[96] Lemon J (2006). Plotrix: a package in the red light district of R. R-news..

[97] Layman CA, Arrington DA, Montaña CG, Post DM (2007). Can stable isotope ratios provide for community-wide measures of trophic structure?. Ecology..

[98] Davis AK, Maney DL, Maerz JC (2008). The use of leukocyte profiles to measure stress in vertebrates: a review for ecologists. Funct Ecol.

[99] Plischke A, Quillfeldt P, Lubjuhn T, Merino S, Masello JF (2010). Leucocytes in adult burrowing parrots *Cyanoliseus patagonus* in the wild: variation between contrasting breeding seasons, gender and condition. J Ornithol.

[100] Merino S, Martínez J, Møller AP, Sanabria L, de Lope F, Pérez J (1999). Phytohaemagglutinin injection assay and physiological stress in nestling house martins. Anim Behav.

[101] Dein FJ. Hematology. In: Harrison GJ, Harrison WR, editors. Clinical avian medicine and surgery: W. B. Saunders Company; 1986. p. 174–91.

[102] Hawkey CM, Dennet PB. A colour atlas of comparative veterinary haematology: Wolfe; 1989.

[103] Humphries GRW, Naveen R, Schwaller M, Che-Castaldo C, McDowall P, Schrimpf M, Lynch HJ (2017). Mapping application for penguin populations and projected dynamics (MAPPPD): data and tools for dynamic management and decision support. Polar Rec.

[104] Naveen R, Forrest S, Dagit R, Blight L, Trivelpiece W, Trivelpiece S (2000). Censuses of penguin, blue-eyed shag, and southern giant petrel populations in the Antarctic Peninsula region, 1994–2000. Polar Rec.

[105] Hucke-Gaete R, Osman LP, Moreno CA, Torres D (2004). Examining natural population growth from near extinction: the case of the Antarctic fur seal at the South Shetlands, Antarctica. Polar Biol.

[106] Atkinson A, Hill SL, Pakhomov EA, Siegel V, Anadon R, Sanae C (2017). KRILLBASE: a circumpolar database of Antarctic krill and salp numerical densities, 1926-2016. Earth Sys Sci Data.

[107] CCAMLR. Krill fishery report 2018: Commission for the Conservation of Antarctic Marine Living Resources; 2018.

[108] Viñuela J, Moreno J, Carrascal LM, Sanz JJ, Amat JA, Ferrer M (1996). The effect of hatching date on parental care, chick growth, and chick mortality in the chinstrap penguin *Pygoscelis antarctica*. J Zool.

[109] Lynch H, Fagan W, Naveen R (2010). Population trends and reproductive success at a frequently visited penguin colony on the western Antarctic Peninsula. Polar Biol.

[110] Dunn MJ, Forcada J, Jackson JA, Waluda CM, Nichol C, Trathan PN (2019). A long-term study of gentoo penguin (*Pygoscelis papua*) population trends at a major Antarctic tourist site, Goudier Island, Port Lockroy. Biodivers Conserv.

[111] Cairns DK (1989). The regulation of seabird colony size: a hinterland model. Am Nat.

[112] Carpenter-Kling T, Handley JM, Green DB, Reisinger RR, Makhado AB, Crawford RJM, Pistorius PA (2017). A novel foraging strategy in gentoo penguins breeding at sub-Antarctic Marion Island. Mar Biol.

[113] BirdLife International. IUCN red list for birds. 2020. http://www.birdlife.org. Accessed 28 Oct 2020.

[114] Polito MJ, Trivelpiece WZ, Karnovsky NJ, Ng E, Patterson WP, Emslie SD (2011). Integrating stomach content and stable isotope analyses to quantify the diets of pygoscelid penguins. PLoS One.

[115] Polito MJ, Trivelpiece WZ, Patterson WP, Karnovsky NJ, Reiss CS, Emslie SD (2015). Contrasting specialist and generalist patterns facilitate foraging niche partitioning in sympatric populations of Pygoscelis penguins. Mar Ecol Prog Ser.

[116] Herman RW, Valls FCL, Hart T, Petry MV, Trivelpiece WZ, Polito MJ (2017). Seasonal consistency and individual variation in foraging strategies differ among and within *Pygoscelis* penguin species in the Antarctic peninsula region. Mar Biol.

[117] Dimitrijević D, Paiva VH, Ramos JA, Seco J, Ceia FR, Chipev N, Valente T, Barbosa A, Xavier JC (2018). Isotopic niches of sympatric Gentoo and chinstrap penguins: evidence of competition for Antarctic krill?. Polar Biol.

[118] Panasiuk A, Wawrzynek-Borejko J, Musiał A, Korczak-Abshire M (2020). *Pygoscelis* penguin diets on King George Island, South Shetland Islands, with a special focus on the krill *Euphausia superba*. Antarct Sci.

[119] du Toit M, Bartlett P, Bester M, Roux J. Seabird predation by individual seals at Ichaboe Island, Namibia. S Afr J Wildl Res. 2004;34:45–54. https://hdl.handle.net/10520/EJC117184.

[120] Visser IN, Drennan MP, White RW, MacLean SF, Lagerstrom LC, Francis JM (2008). Antarctic fur seals (*Arctocephalus gazella*) observed predating Adélie (*Pygoscelis adeliae*) and chinstrap penguins (*P. antarctica*), Antarctic Peninsula. Aquat Mamm.

[121] D’Amico VL, Bertellotti M, Benzal J, Coria N, Vidal V, Diaz JI (2016). Leukocyte counts in different populations of Antarctic Pygoscelid penguins along the Antarctic Peninsula. Polar Biol.

[122] Shepard E, Cole E-L, Neate A, Lempidakis E, Ross A (2019). Wind prevents cliff-breeding birds from accessing nests through loss of flight control. eLife.

