# Supplementary material for: How animals distribute themselves in space: energy landscapes of Antarctic avian predators
Source: Mov Ecol. 2021 May 17;9:24. doi: 10.1186/s40462-021-00255-9 (PMC8127181; doi:10.1186/s40462-021-00255-9)
Supplement: Supplementary file 1 — Additional file 1: Table S1. Results of a General Additive Model (GAM) investigating the sum of Overall Dynamic Body Acceleration (ODBA) during dive as a function of maximum dive depth with the intra-depth zone (IDZ; foraging dives performed by the individuals split in benthic and pelagic), as factor. Gentoo penguins Pygoscelis papua were breeding at Devils Point, Byers Peninsula, Livingston Island, South Shetland Islands, Antarctica, while chinstrap penguins Pygoscelis antarcticus were breeding at Vapour Col rookery, Deception Island, South Shetland Islands, Antarctica. Data was obtained during chick guard. Table S2. The relationship between the sum of Overall Dynamic Body Acceleration (ODBA) during dive and maximum dive depth for benthic and pelagic dives (based on the index of benthic diving behaviour, intra-depth zone; IDZ). Gentoo penguins Pygoscelis papua were breeding at Devils Point, Byers Peninsula, Livingston Island, South Shetland Islands, Antarctica, while chinstrap penguins Pygoscelis antarcticus were breeding at Vapour Col rookery, Deception Island, South Shetland Islands, Antarctica. Data was obtained during chick guard. Table S3. The relationship between the number of dives per trip and the maximum distance from the colony during a foraging trip carried out by gentoo penguin Pygoscelis papua breeding at Devils Point, Byers Peninsula, Livingston Island, South Shetland Islands, Antarctica (chick guard; December 2016). See also Figure S11. Table S4. Results of a General Additive Model (GAM) investigating the bottom time as a function of event maximum depth (maximum depth [m] reached during dive event) with the intra-depth zone (IDZ; foraging dives performed by the individuals split in benthic and pelagic), as factor. Gentoo penguins Pygoscelis papua were breeding at Devils Point, Byers Peninsula, Livingston Island, South Shetland Islands, Antarctica, while chinstrap penguins Pygoscelis antarcticus were breeding at Vapour Col rookery, Deception Island, S [file 40462_2021_255_MOESM1_ESM.docx]

**Additional file 1**

**How animals distribute themselves in space: energy landscapes of Antarctic avian predators**

Juan F. Masello*, Andres Barbosa, Akiko Kato, Thomas Mattern, Renata Medeiros, Jennifer E. Stockdale, Marc N. Kümmel, Paco Bustamante, Josabel Belliure, Jesús Benzal, Roger Colominas-Ciuró, Javier Menéndez-Blázquez, Sven Griep, Alexander Goesmann, William O. C. Symondson and Petra Quillfeldt

* Correspondence: [juan.f.masello@bio.uni-giessen.de](mailto:juan.f.masello@bio.uni-giessen.de)

Content:

Additional Tables S1 to S12

Additional Figures S1 to S30

Additional Methods

Additional References

**Table S1**. Results of a General Additive Model (GAM) investigating the sum of Overall Dynamic Body Acceleration (ODBA) during dive as a function of maximum dive depth with the intra-depth zone (IDZ; foraging dives performed by the individuals split in benthic and pelagic), as factor. Gentoo penguins *Pygoscelis papua* were breeding at Devils Point, Byers Peninsula, Livingston Island, South Shetland Islands, Antarctica, while chinstrap penguins *Pygoscelis antarcticus* were breeding at Vapour Col rookery, Deception Island, South Shetland Islands, Antarctica. Data was obtained during chick guard.

|  |  | *n* | d.f. | *F* | *P* |
| --- | --- | --- | --- | --- | --- |
| Gentoos | dive depth | 16938 | 1 | 30674.3 | < 0.001 |
|  | IDZ |  | 1 | 438.3 | < 0.001 |
| Chinstraps | dive depth | 11172 | 1 | 27701.6 | < 0.001 |
|  | IDZ |  | 1 | 93.8 | < 0.001 |

**Table S2**. The relationship between the sum of Overall Dynamic Body Acceleration (ODBA) during dive and maximum dive depth for benthic and pelagic dives (based on the index of benthic diving behaviour, intra-depth zone; IDZ). Gentoo penguins *Pygoscelis papua* were breeding at Devils Point, Byers Peninsula, Livingston Island, South Shetland Islands, Antarctica, while chinstrap penguins *Pygoscelis antarcticus* were breeding at Vapour Col rookery, Deception Island, South Shetland Islands, Antarctica. Data was obtained during chick guard.

| **IDZ** | **regression** | ***R^2^*** | **Description** | Paramenters | | | | |
| --- | --- | --- | --- | --- | --- | --- | --- | --- |
|  |  |  |  | **a** | **b** | **c** | **d** | **y0** |
| Gentoos | | | | | | | | |
| Benthic | f=a*x/(b+x)+c*x/(d+x) | 0.70 | Hyperbola, Double Rectangular, 4 Parameter | 8.1 | 6.4 | 52.0 | 34.3 |  |
| Pelagic | f=y0+a*x^b | 0.75 | Power, 3 Parameter | 38.2 | 0.2 |  |  | -42.5 |
| Chinstraps | | | | | | | | |
| Benthic | f=a*x/(b+x)+c*x/(d+x) | 0.78 | Hyperbola, Double Rectangular, 4 Parameter | 10.0 | 3.9 | 57.7 | 67.3 |  |
| Pelagic | f=a*x/(b+x)+c*x/(d+x) | 0.75 | Hyperbola, Double Rectangular, 4 Parameter | 18.7 | 7.9 | 131.0 | 356.7 |  |

**Table S3.** The relationship between the number of dives per trip and the maximum distance from the colony during a foraging trip carried out by gentoo penguin *Pygoscelis papua* breeding at Devils Point, Byers Peninsula, Livingston Island, South Shetland Islands, Antarctica (chick guard; December 2016). See also **Figure S11.**

| **regression** | ***R^2^*** | **Description** | Paramenters | | | |
| --- | --- | --- | --- | --- | --- | --- |
|  |  |  | **a** | **b** | **c** | **y0** |
| f=y0+a*x+b*x^2+c*x^3 | 0.69 | Polynomial, Cubic | -3.95 | -0.0025 | 0.0182 | 227.3 |

**Table S4**. Results of a General Additive Model (GAM) investigating the bottom time as a function of event maximum depth (maximum depth [m] reached during dive event) with the intra-depth zone (IDZ; foraging dives performed by the individuals split in benthic and pelagic), as factor. Gentoo penguins *Pygoscelis papua* were breeding at Devils Point, Byers Peninsula, Livingston Island, South Shetland Islands, Antarctica, while chinstrap penguins *Pygoscelis antarcticus* were breeding at Vapour Col rookery, Deception Island, South Shetland Islands, Antarctica. Data was obtained during chick guard.

|  |  | *n* | d.f. | *F* | *P* |
| --- | --- | --- | --- | --- | --- |
| Gentoos | dive depth | 17124 | 1 | 7530.0 | < 0.001 |
|  | IDZ |  | 1 | 1158.0 | < 0.001 |
| Chinstraps | dive depth | 12696 | 1 | 8697.6 | < 0.001 |
|  | IDZ |  | 1 | 407.5 | < 0.001 |

**Table S5**. The relationship between bottom time and event maximum depth for benthic and pelagic dives (based on the index of benthic diving behaviour, intra-depth zone; IDZ). Gentoo penguins *Pygoscelis papua* were breeding at Devils Point, Byers Peninsula, Livingston Island, South Shetland Islands, Antarctica, while chinstrap penguins *Pygoscelis antarcticus* were breeding at Vapour Col rookery, Deception Island, South Shetland Islands, Antarctica. Data was obtained during chick guard.

| **IDZ** | **regression** | ***R^2^*** | **Description** | Paramenters | | | | |  |
| --- | --- | --- | --- | --- | --- | --- | --- | --- | --- |
|  |  |  |  | **a** | **b** | **c** | **x0** | **y0** | |
| Gentoos | | | | | | | | |  |
| Benthic | f=x/(a+b*x) | 0.32 | Hyperbola, Modified Hyperbola II | 0.33 | 0.02 |  |  |  | |
| Pelagic | f = a*x/(b+x) | 0.38 | Hyperbola; Single Rectangular, 2 Parameter | 48.0 | 17.7 |  |  |  | |
| Chinstraps | | | | | | | | |  |
| Benthic | f=y0+a/(1+exp(-(x-x0)/b))^c | 0.47 | Sigmoidal, Sigmoid, 5 Parameter | 38.0 | 6.7 | 0.3 | 28.9 | -4.4 | |
| Pelagic | f=y0+a*(1-exp(-b*x))^c | 0.47 | Sigmoidal, Chapman, 4 Parameter | 29.2 | 0.03 | 1.009 |  | 4.1 | |

**Table S6**. The number of DNA extractions from scat samples of gentoo penguins *Pygoscelis papua* breeding at New Island, Falkland/Malvinas Islands, during chick guard (December) in 2013 and 2014, gentoo penguins breeding at Byers Peninsula, Livingston Island, Antarctica, during chick guard (December 2016), and chinstrap penguins *Pygoscelis antarcticus* breeding at Vapour Col rookery, Deception Island, South Shetland Islands, Antarctica, during chick guard (January 2017).

|  | Gentoos | | | |  | Chinstraps |
| --- | --- | --- | --- | --- | --- | --- |
|  | Dec 2013 | Dec 2014 | | Dec 2016 |  | Jan 2017 |
|  | South End | South End | North End | Byers Peninsula |  | Deception Island |
| Adults | 17 | 6 | 3 | 36 |  | 19 |
| First chicks | 24 | 4 | 4 | 11 |  | 23 |
| Second chicks | 15 | 6 | − | 5 |  | 23 |
| Unknown age individuals | 17 | 15 | 22 | − |  | − |

*Note*: sample sizes vary as not all individuals defecated during handling. Apart from logger deployment and recovery, the birds were not disturbed in order to avoid influencing nestling attendance behaviour. The North End colony was only briefly accessible in 2013−4 preventing us to take faecal samples (Masello et al. 2017, <https://frontiersinzoology.biomedcentral.com/articles/10.1186/s12983-017-0219-8> ).

**Table S7**. Control samples for the molecular detection of prey in scat samples from the studied penguins.

| Classification | Species | Sampling location | n |
| --- | --- | --- | --- |
| Arthropoda, Crustacea, Malacostraca, Euphausiacea | *Euphausia superba* Antarctic Krill | King George Island, South Shetland Islands, Antarctica | 1 |
|  |  | Deception Island, South Shetland Islands, Antarctica | 7 |
| Chordata, Thaliacea, Salpida, Salpidae | *unknown* | Deception Island, South Shetland Islands, Antarctica | 1 |
| Chordata, Vertebrata, Actinopterygii, Teleostei, Perciformes, Harpagiferidae | *Harpagifer* sp. | King George Island, South Shetland Islands, Antarctica | 1 |
| Chordata, Vertebrata, Actinopterygii, Teleostei, Perciformes, Nototheniidae | *Notothenia rossii* | King George Island, South Shetland Islands, Antarctica | 1 |
|  | *Notothenia coriiceps* | King George Island, South Shetland Islands, Antarctica | 1 |
|  | *Pleuragramma antarctica* | King George Island, South Shetland Islands, Antarctica | 1 |
|  | *Trematomus newnesi* | King George Island, South Shetland Islands, Antarctica | 1 |

**Table S8**. List of primers used in this study for the detection of prey species in scat samples from gentoo penguins *Pygoscelis papua* and chinstrap penguins *Pygoscelis antarcticus*.

| **Prey Target** | **Gene** | **Primer name** | **Sequence 5’-3’** | **Annealing temperature (°C)** | **Amplicon size (bp)** | **Reference** |
| --- | --- | --- | --- | --- | --- | --- |
| *mtDNA markers* | | | | | | |
| Euphausiidae | 12S | EuphMLSUF  EuphMLSUR | TTTATTGGGGCGATAAAAAT  TCGAGGTCGYAATCTTTCTTGT | 54 | 169 | Deagle et al. 2007 |
| Metazoa | COI | mlCOlintF  jgHCO2198 | GGWACWGGWTGAACWGTWTAYCCYCC  TAIACYTCIGGRTGICCRAARAAYCA | 48 | 313 | Leray et al. 2013 |
| Osteichthyes | 12S | FishF1  FishR1 | CGGTAAAACTCGTGCC  CCGCCAAGTCCTTTGGG | 56 | ~300 | Jarman unpubl., in Medeiros-Mirra 2010 |
| *Nuclear markers* | | | | | | |
| Amphipoda | 18S rDNA | AmphNSSF1  AmphNSSR1 | CTGCGGTTAAAAGGCTCGTAGTTGAA  ACTGCTTTRAGCACTCTGATTTAC | 51 | 204–375 | Jarman et al. 2006 |
| Cephalopoda | 28S rDNA | Squid28SF  Squid28SR | CGCCGAATCCCGTCGCMAGTAAAMGGCTTC  CCAAGCAACCCGACTCTCGGATCGAA | 60 | ~180 | Deagle et al. 2005 |

**Table S9.** Comparison of total foraging costs per bottom time gain (J kg^-1^ s^-1^) using Kruskal-Wallis rank sum test. The groups tested correspond to gentoo penguins *Pygoscelis papua* breeding at New Island, Falkland/Malvinas Islands, during chick guard (December) in 2013 and 2014, gentoo penguins breeding at Devils Point, Byers Peninsula, Livingston Island, South Shetland Islands, Antarctica, during chick guard (December 2016), and chinstrap penguins *Pygoscelis antarcticus* breeding at Vapour Col rookery, Deception Island, South Shetland Islands, Antarctica, during chick guard (January 2017).

|  | Gentoos  New I. South  2013 | Gentoos  New I. South  2014 | Gentoos  New I. North  2014 | Gentoo  Devils Point  short trips | Gentoo  Devils Point  long trips |
| --- | --- | --- | --- | --- | --- |
| Gentoos  New I. South, 2014 | 56.5  **< 0.001** | − | − | − | − |
| Gentoos  New I. North, 2014 | -100.5  **< 0.001** | -29.2  **< 0.001** | − | − | − |
| Gentoo  Devils Point, short trips | -57.2  **< 0.001** | 4.2  **< 0.001** | 35.9  **< 0.001** | − | − |
| Gentoo  Devils Point, long trips | -25.9  **< 0.001** | 18. 5  **< 0.001** | 45.1  **< 0.001** | 15.7  **< 0.001** | − |
| Chinstraps  Vapor Col | -145.0  **< 0.001** | -34.8  **< 0.001** | 0.349  0.364 | -44.4  **< 0.001** | -51.5  **< 0.001** |

The upper value in a cell corresponds to χ^2^, the lower value corresponds to *P*-value. Significant P-values marked **bold**

**Table S10**. Best blast results for each detected taxa and corresponding accession number, the identity with the blast reference sequence, the sequence length and the bitscore from gentoo penguins *Pygoscelis papua* breeding at New Island, Falkland/Malvinas Islands, during chick guard (December) in 2013 and 2014, gentoo penguins breeding at Devils Point, Byers Peninsula, Livingston Island, South Shetland Islands, Antarctica, during chick guard (December 2016), and chinstrap penguins *Pygoscelis antarcticus* breeding at Vapour Col rookery, Deception Island, South Shetland Islands, Antarctica, during chick guard (January 2017).

| Taxa | Accession number | Ident % (blast) | Sequence length | E-value | Bit-score |  |
| --- | --- | --- | --- | --- | --- | --- |
| Arthropoda, Crustacea, Malacostraca | | | | | |  |
| Amphipoda, Hyperiidae | | | | | |  |
| *Themisto* sp. | DQ378048.1 | 99 | 183 | 7e-88 | 333 |  |
| Decapoda | | | | | |  |
| …Pandalidae (shrimps) | | | | | |  |
| *Pandalus* sp. | KJ193758.1 | 99 | 142 | 2e-64 | 255 |  |
| …Galatheidae | | | | | |  |
| *Munida gregaria*  Lobster krill | KU521508.1 | 100 | 313 | 2e-161 | 579 |  |
| Euphausiacea | | | | | |  |
| *Euphausia superba*  Antarctic Krill | JQ286350.1 | 100 | 128 | 9.48e-56 | 226 |  |
| *Thysanoessa macrura* | Z73802.1 | 99.2 | 127 | 7.25e-57 | 230 |  |
| Mollusca, Cephalopoda | | | | | |  |
| Octopoda | | | | | |  |
| *Enteroctopus megalocyathus*  Southern Red Octopus | KF774312.1 | 100 | 313 | 2e-161 | 579 | |
| Oegopsida | | | | | |  |
| Onychoteuthidae (squids) | | | | | |  |
| Moroteuthis sp. | AY176331.1 | 99 | 124 | 4e-55 | 224 |  |

**Table S10**. (cont.)

| Taxa | Accession number | Ident % (blast) | Sequence length | E-value | Bit-score | |
| --- | --- | --- | --- | --- | --- | --- |
| Ommastrephidae (squids) | KY387931.1 | 98 | 123 | 4e-51 | 211 | |
| Pyroteuthidae (fire squids) | KY387932.1 | 98 | 125 | 9e-52 | 213 | |
| Sepida | | | | | |  |
| Sepiolidae (bobtail squids) | AJ310242.1 | 98 | 120 | 2e-48 | 202 | |
| Chordata, Vertebrata, Actinopterygii, Teleostei | | | | | | |
| Clupeiformes | | | | | | |
| Clupeidae | | | | | | |
| *Sprattus* sp. (spratts) | AP011607.1 | 99 | 271 | 1e-134 | 490 | |
| Gadiformes | | | | | | |
| Gadidae (codfishes) | FR751401.1 | 98 | 269 | 1e-128 | 470 | |
| *Micromesistius* sp.  (blue whitings) | FR751401.1 | 99 | 269 | 3e-135 | 492 | |
| Myctophiformes | | | | | | |
| Myctophidae | AP012248.1 | 98 | 270 | 3e-129 | 472 | |
| Electrona sp. | AP012248.1 | 99 | 270 | 7e-131 | 477 | |
| *Electrona antarctica*  Antarctic Lanternfish | KF713404.1 | 99.1 | 311 | 2e-155 | 558 | |
| *Gymnoscopelus nicholsi*  Nichol's Lanternfish | AP012250.1 | 99.3 | 270 | 2.70e-134 | 488 | |
| Perciformes |  |  |  |  |  | |
| Channichthyidae  (crocodile icefishes) | GU217678.1 | 98 | 268 | 4e-128 | 468 | |

**Table S10**. (cont.)

| Taxa | Accession number | Ident % (blast) | Sequence length | E-value | Bit-score |
| --- | --- | --- | --- | --- | --- |
| *Chaenodraco wilsoni*  Spiny Icefish | JN640812.1 | 99.7 | 308 | 5.16e-157 | 564 |
| *Champsocephalus gunnari*  Icefish | GU217678.1 | 100 | 268 | 2e-136 | 496 |
| *Chionodraco* sp. | KU341409.1 | 98.5 | 269 | 2.10e-130 | 475 |
| *Cryodraco antarcticus*  Long-fingered Icefish | JN640869.1 | 99.03 | 310 | 8.63e-155 | 556 |
| Nototheniidae | | | | | |
| *Dissostichus eleginoides*  Chilean Sea Bass | AB723627.1 | 100 | 269 | 6e-137 | 497 |
| *Notothenia coriiceps*  Black Rockcod | EU326389.1 | 99.7 | 308 | 5.16e-157 | 564 |
| *Patagonotothen* sp. | KY773221.1 | 98 | 313 | 4e-153 | 551 |
| *Patagonotothen tessellata*  Black Southern Cod | KY773217.1 | 100 | 313 | 2e-161 | 579 |
| *Patagonotothen wiltoni* | KY773206.1 | 100 | 313 | 2e-161 | 579 |
| *Paranotothenia* sp. | AY227780.1 | 98.6 | 70 | 8.71e-25 | 124 |
| Agonidae (alligatorfishes) | KM057995.1 | 99 | 268 | 2e-131 | 479 |
| Psychrolutidae (blobfishes) | | | | | |
| *Psychrolutes* sp. | KM057982.1 | 99 | 268 | 4e-133 | 484 |

**Table S11**. Diet and isotopic niche metrics. Data correspond to gentoo penguins *Pygoscelis papua* breeding at Devils Point, Byers Peninsula, Livingston Island, South Shetland Islands, Antarctica, during chick guard (December 2016), and chinstrap penguins *Pygoscelis antarcticus* breeding at Vapour Col rookery, Deception Island, South Shetland Islands, Antarctica, during chick guard (January 2017).

|  | Gentoo  female  adults | Gentoo  male  adults | Gentoo  first  chicks | Gentoo  second  chicks | Chinstrap  adults | Chinstrap  chicks |
| --- | --- | --- | --- | --- | --- | --- |
| N | 10 | 15 | 24 | 24 | 6 | 11 |
| δ^13^C | -25.12±0.49 ^a,b^ | -24.84±0.40 ^a^ | -25.43±0.54 ^b,c^ | -25.48±0.53^b,c^ | -25.98±0.15 ^c,d^ | -26.56±0.09 ^d^ |
| δ^15^N | 8.26±0.41 ^a^ | 8.48±0.65 ^a^ | 8.13±0.64 ^a^ | 8.15±0.66 ^a^ | 7.98±0.10 ^a,b^ | 7.36±0.17 ^b^ |
| SEA | 0.48 | 0.74 | 0.72 | 0.75 | 0.04 | 0.05 |
| SEAc | 0.55 | 0.80 | 0.75 | 0.78 | 0.05 | 0.06 |
| SEAb | 0.51 | 0.72 | 0.74 | 0.77 | 0.04 | 0.05 |
| NR | 1.65 | 2.45 | 2.24 | 2.64 | 0.31 | 0.58 |
| CR | 1.48 | 1.62 | 1.71 | 2.00 | 0.39 | 0.34 |
| CD | 0.55 | 0.62 | 0.70 | 0.70 | 0.17 | 0.17 |
| NND | 0.33 | 0.29 | 0.16 | 0.20 | 0.12 | 0.10 |

Dunn tests were used for pairwise comparisons, and homogenous subsets are marked with superscript letters. SEA: area of the standard ellipse (isotope niche width). SEAc: as SEA, corrected for sample size. SEAb: Bayesian standard ellipse area. NR: trophic length (range in δ^15^N). CR: diversity of basal resources (range in δ^13^C). CD: niche width 2 (mean distance to centroid). NND: mean nearest neighbour distance.

**Table S12**. Isotopic niche metrics of gentoo penguins *Pygoscelis papua* breeding at New Island, Falkland/Malvinas Islands, during chick guard (December) in 2013 and 2014. Parameters are based on carbon (δ^13^C) and nitrogen (δ^15^N) stable isotopes of chick feather samples as a marker of breeding season foraging ecology from two colonies at New Island and two breeding seasons calculated with the SIAR package. SE: South End colony. NE: North End colony. For further details see Masello et al. (2017).

|  | New I., South  Dec 2013 | New I., North  Dec 2013 | New I., South  Dec 2014 | New I., North  Dec 2014 |
| --- | --- | --- | --- | --- |
| N | 20 | 20 | 18 | 18 |
| δ^13^C | -15.49 | -15.73 | -15.94 | -16.04 |
| δ^15^N | 14.39 | 14.45 | 14.71 | 14.75 |
| SEA | 0.63 | 0.34 | 0.19 | 0.15 |
| SEAc | 0.67 | 0.36 | 0.20 | 0.16 |
| NR | 1.74 | 1.46 | 0.99 | 1.40 |
| CR | 2.19 | 0.96 | 0.99 | 0.82 |
| CD | 0.52 | 0.43 | 0.29 | 0.31 |
| NND | 0.26 | 0.16 | 0.15 | 0.13 |

**Table S13**. Gentoo penguins *Pygoscelis papua* breeding success at New Island, Falkland/Malvinas Islands. The number of chicks corresponds to crèche stage. For further details on the colonies see Masello et al. (2010, 2017).

| Year | Number of nests | | |  | Number of hicks | | | Breeding success  (total) |
| --- | --- | --- | --- | --- | --- | --- | --- | --- |
|  | South End colony | North End colony | Total |  | South End colony | North End colony | Total |  |
| 2013 | 2378 | 2044 | 4422 |  | 2458 | 1352 | 3810 | 0.86 |
| 2014 | 2071 | 2072 | 4143 |  | 2171 | 3172 | 5343 | 1.29 |
| 2017 | 1511 | 3108 | 4619 |  | 1020 | 3009 | 4029 | 0.87 |
| 2018 | 1007 | 2285 | 3292 |  | 636 | 1891 | 2527 | 0.77 |
| 2019 | 1241 | 2453 | 3694 |  | 1202 | 2773 | 3975 | 1.08 |

**Figure S1**. The distribution of dive depth data during benthic (A) and pelagic (B) foraging dives by gentoo penguin *Pygoscelis papua* breeding at Devils Point, Byers Peninsula, Livingston Island, South Shetland Islands, Antarctica, during chick guard (December 2016). Benthic and pelagic dives are defined with the use of the index of benthic diving behaviour, intra-depth zone (IDZ).


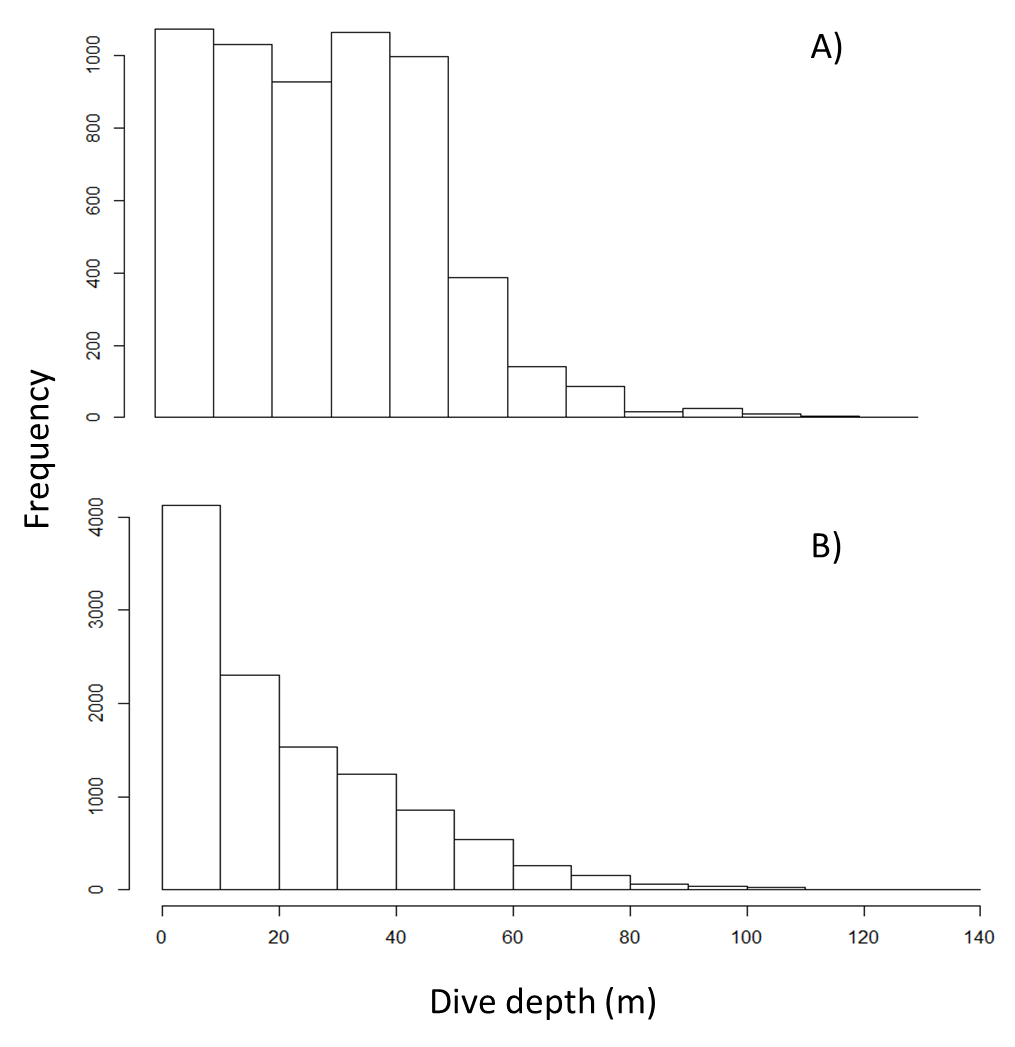


**Figure S2.** The distribution of dive depth data during benthic (A) and pelagic (B) foraging dives by chinstrap penguins *Pygoscelis antarcticus* breeding at Vapour Col rookery, Deception Island, South Shetland Islands, Antarctica, during chick guard (January 2017). Benthic and pelagic dives are defined with the use of the index of benthic diving behaviour, intra-depth zone (IDZ).


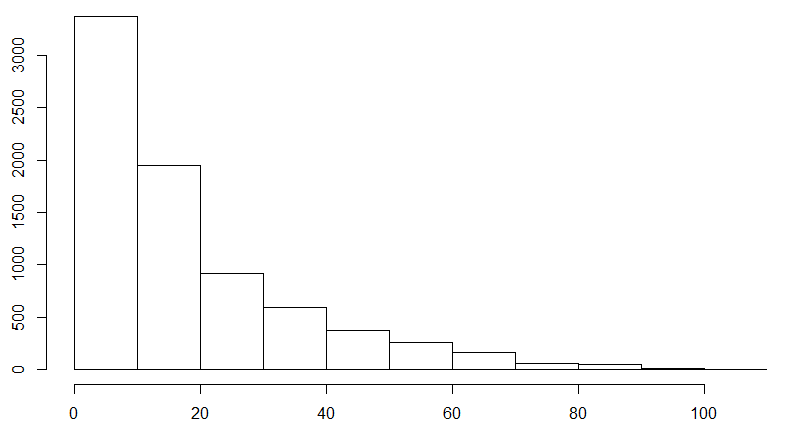

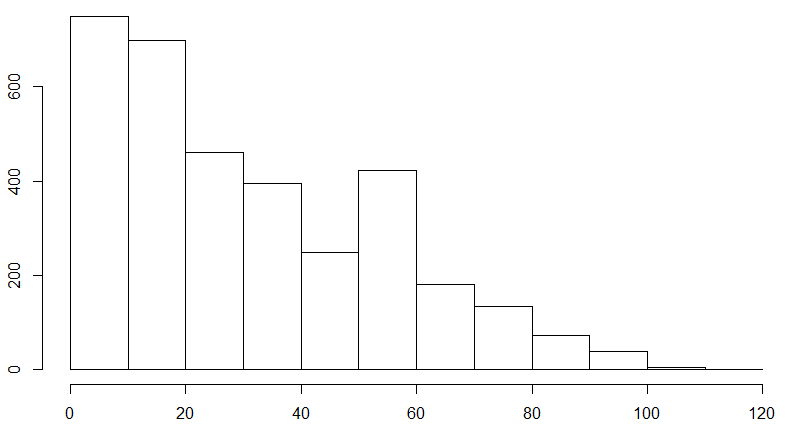


B)

Dive depth (m)

A)

Frequency

**Figure S3.** Example of the distribution in different depths of benthic (A) and pelagic (B) dives carried out by gentoo penguin *Pygoscelis papua* breeding at Devils Point, Byers Peninsula, Livingston Island, South Shetland Islands, Antarctica, during chick guard (December 2016). Depth (in m) is based on data from the International Bathymetric Chart of the Southern Ocean (IBCSO; Arndt et al. 2013).


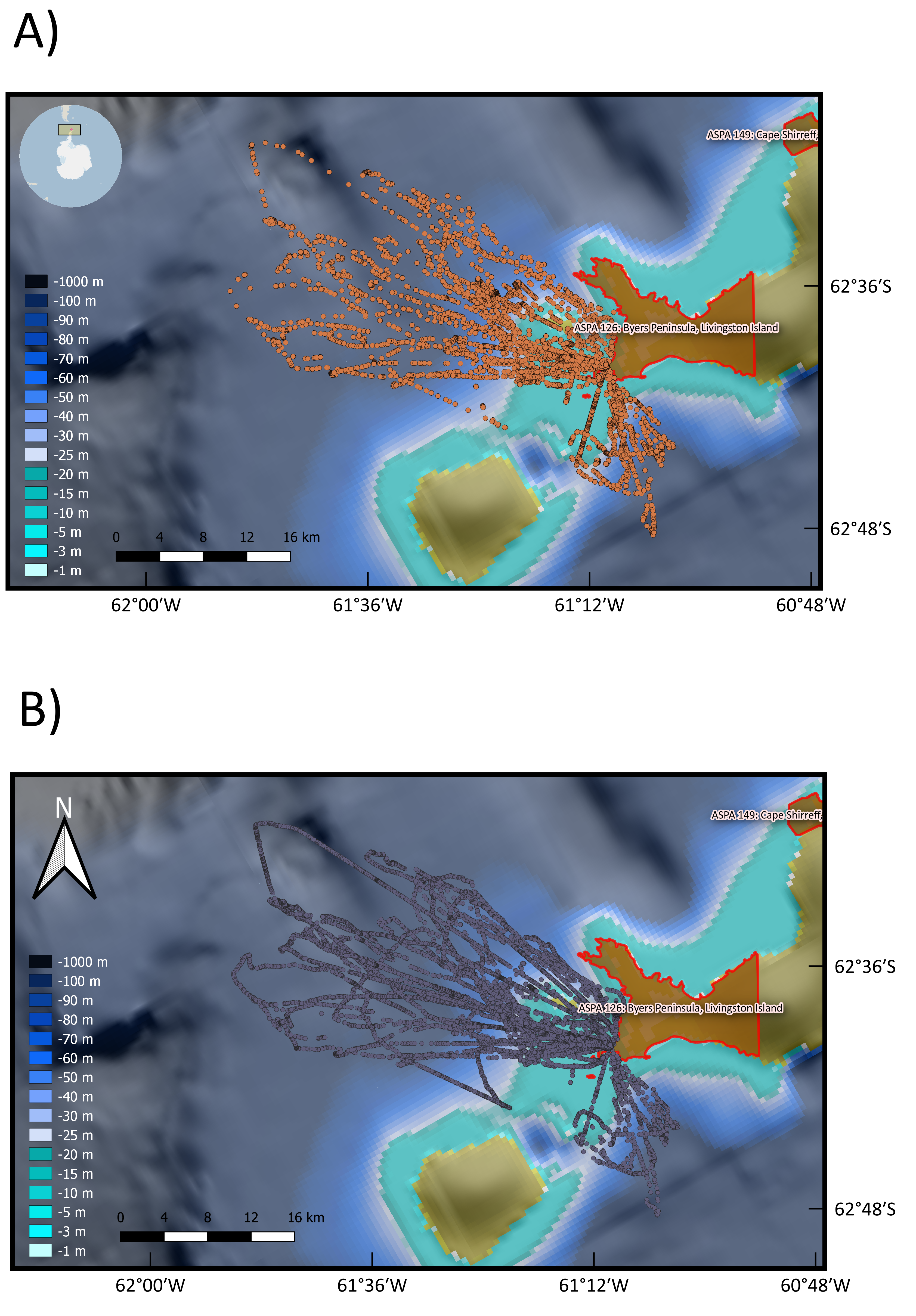


**Figure S4.** Example of the distribution in different depths of benthic (A) and pelagic (B) dives carried out by chinstrap penguins *Pygoscelis antarcticus* breeding at Vapour Col rookery, Deception Island, South Shetland Islands, Antarctica, during chick guard (January 2017). Depth (in m) is based on data from the International Bathymetric Chart of the Southern Ocean (IBCSO; Arndt et al. 2013).


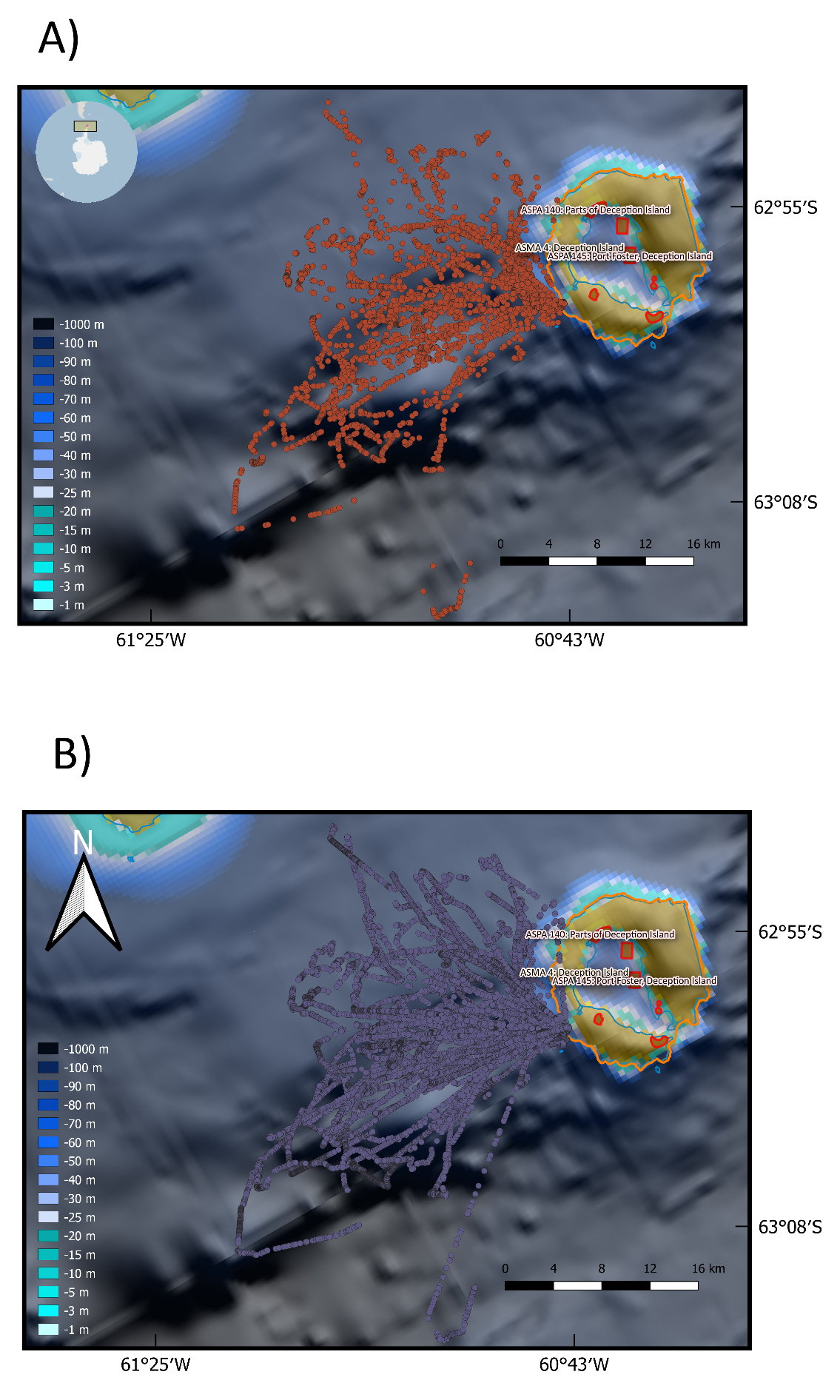


**Figure S5**. Foraging trips by female (red burgundy) and male (yellow) gentoo penguins *Pygoscelis papua* breeding at Devils Point, Byers Peninsula, Livingston Island, South Shetland Islands, Antarctica, during chick guard (December 2016) (A), and chinstrap penguins *Pygoscelis antarcticus* breeding at Vapour Col rookery, Deception Island, South Shetland Islands, Antarctica, during chick guard (January 2017) (B). Depth (m) is based on data from the International Bathymetric Chart of the Southern Ocean (IBCSO; Arndt et al. 2013).

A)


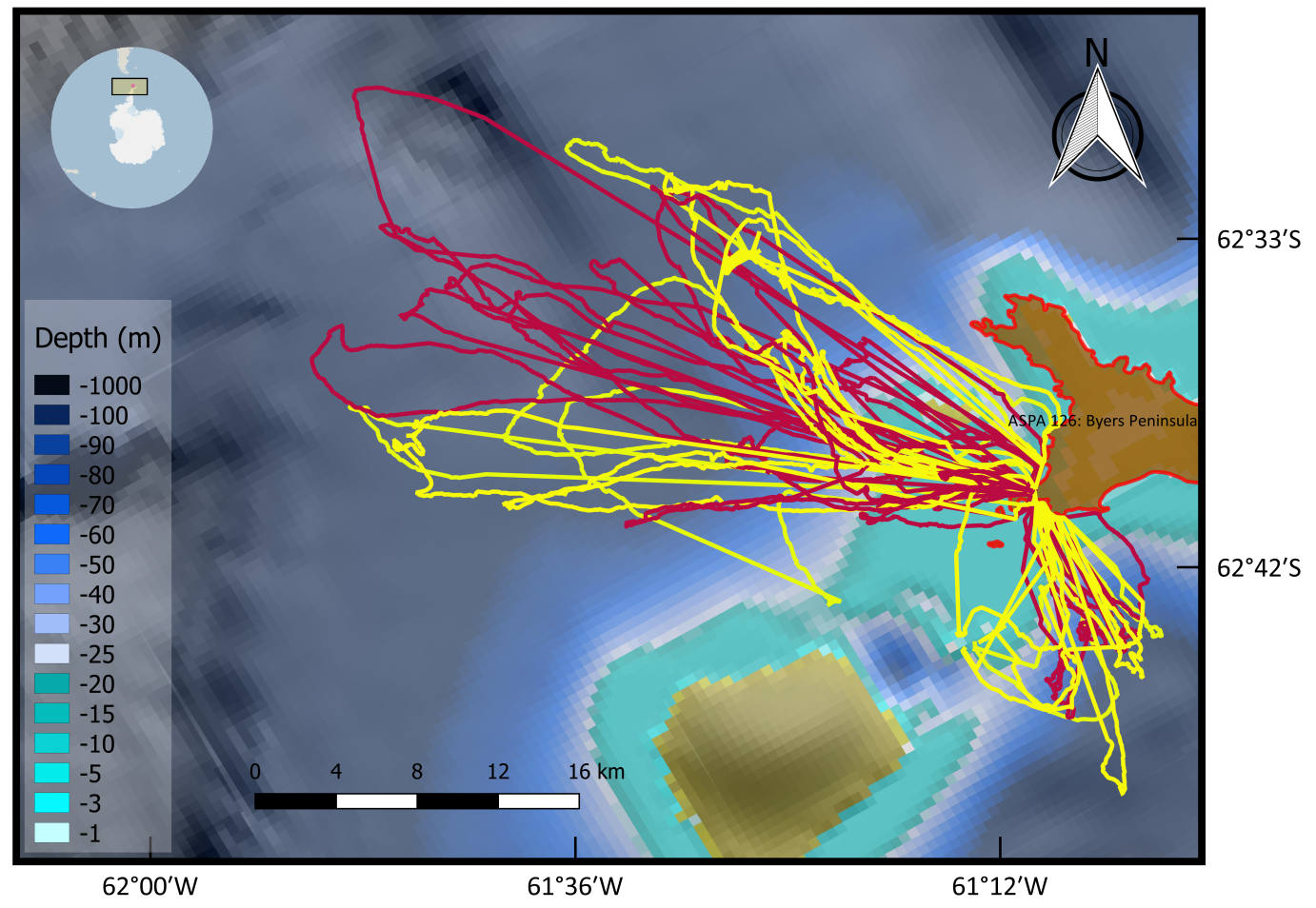


B)


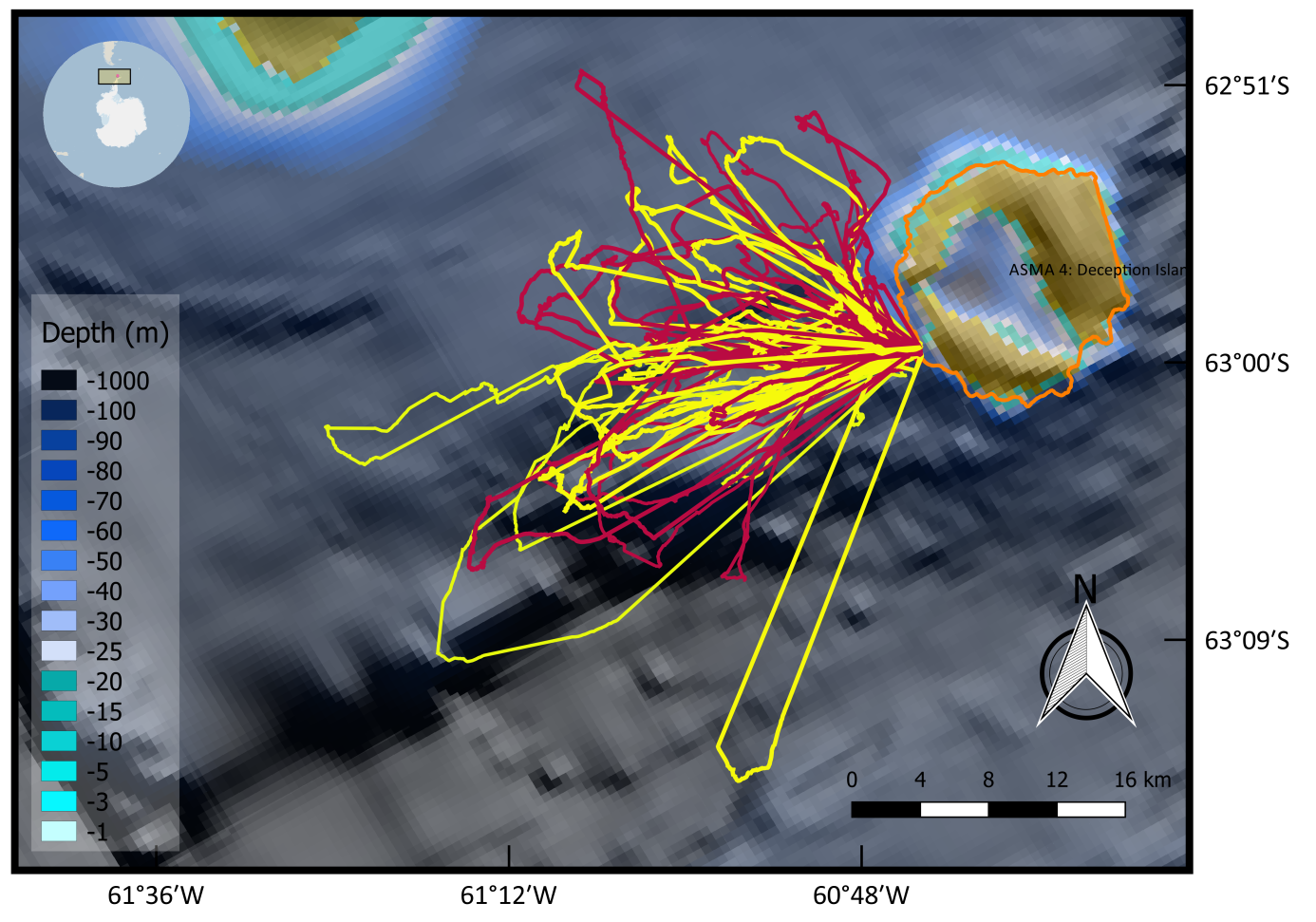


**Figure S6**. Example of dive profiles and tri-axial acceleration data during three consecutive dives by gentoo penguins *Pygoscelis papua* breeding at Devils Point, Byers Peninsula, Livingston Island, South Shetland Islands, Antarctica, during chick guard (December 2016). Acceleration data correspond to each of the three spatial axes: x, surge (green), y, heave (red), and z, sway (blue). Dive depth is given in metres and 0 (zero) corresponds to the water surface.


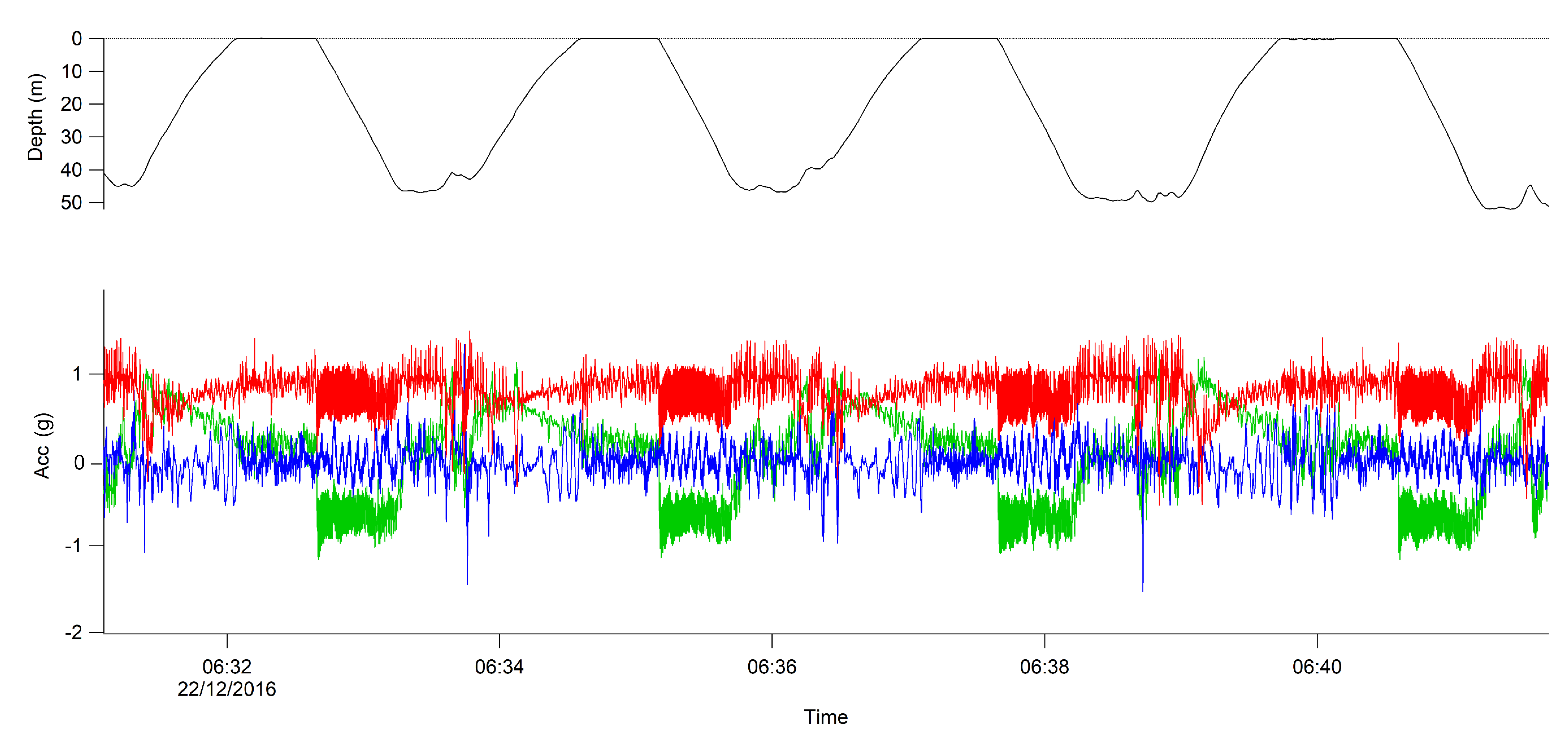


**Figure S7.** The distribution of foraging parameter data used for the calculations of energy landscapes in gentoo penguins *Pygoscelis papua* breeding at Devils Point, Byers Peninsula, Livingston Island, South Shetland Islands, Antarctica, during chick guard (December 2016). See also **Table 1**.


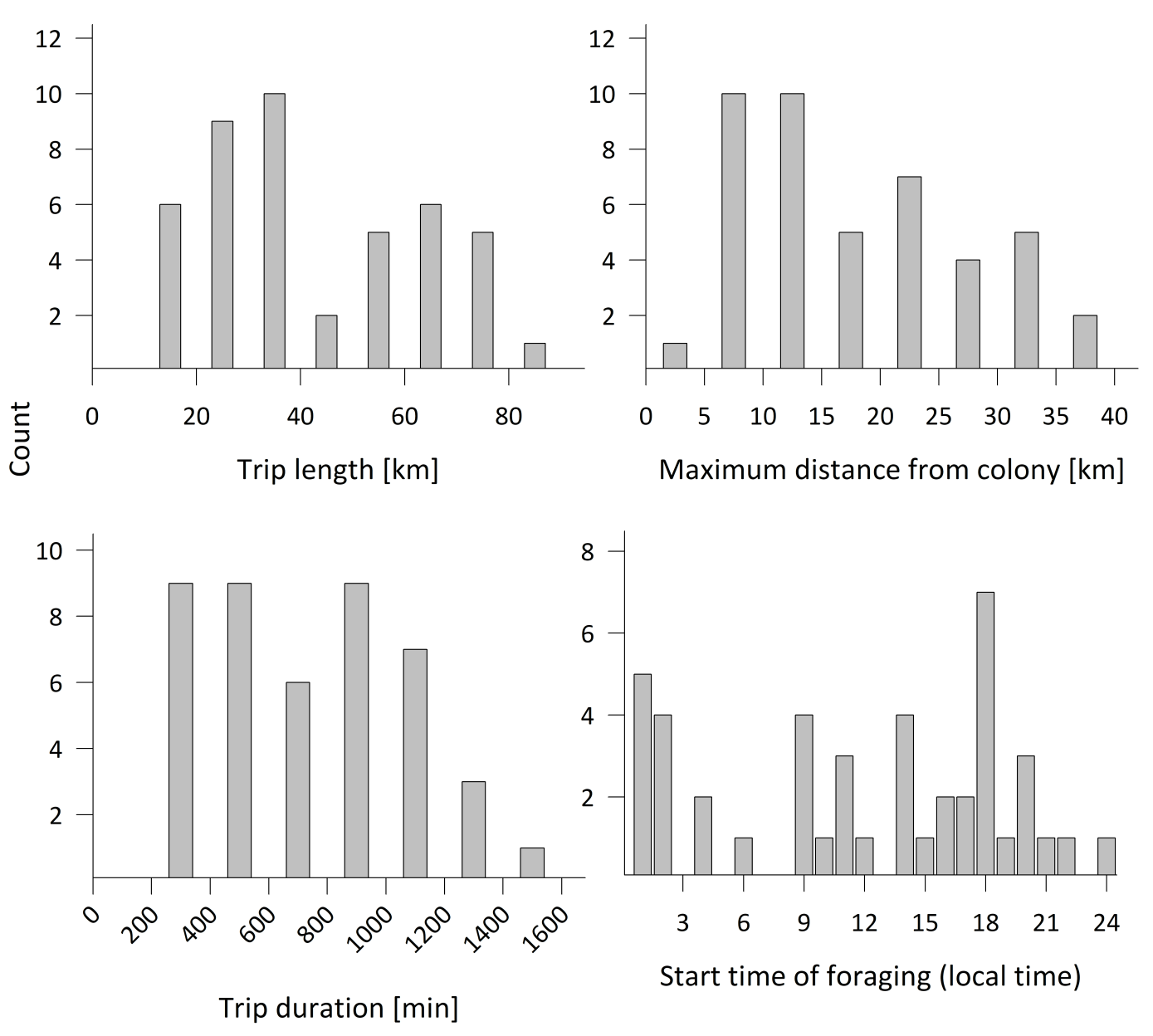


**Figure S8.** The distribution of foraging parameter data used for the calculations of energy landscapes in chinstrap penguins *Pygoscelis antarcticus* breeding at Vapour Col rookery, Deception Island, South Shetland Islands, Antarctica, during chick guard (January 2017). See also **Table 1**.


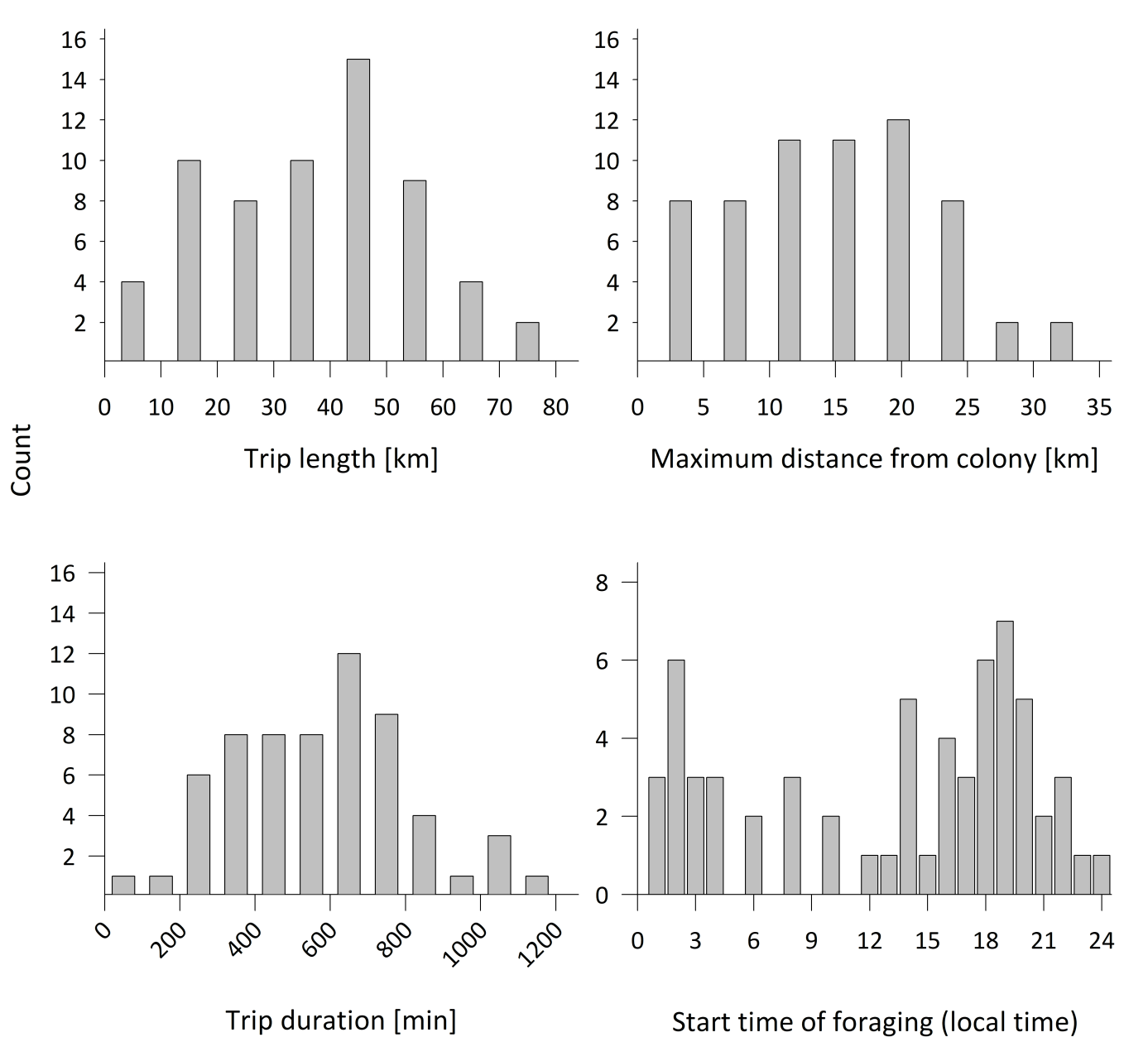


**Figure S9.** The relationship between the sum of Overall Dynamic Body Acceleration (ODBA) during dive and maximum dive depth for benthic dives (based on the index of benthic diving behaviour, intra-depth zone; IDZ) carried out by gentoo penguin *Pygoscelis papua* breeding at Devils Point, Byers Peninsula, Livingston Island, Antarctica, during chick guard (December 2016). Details for the regression curve are given in **Table S2**.


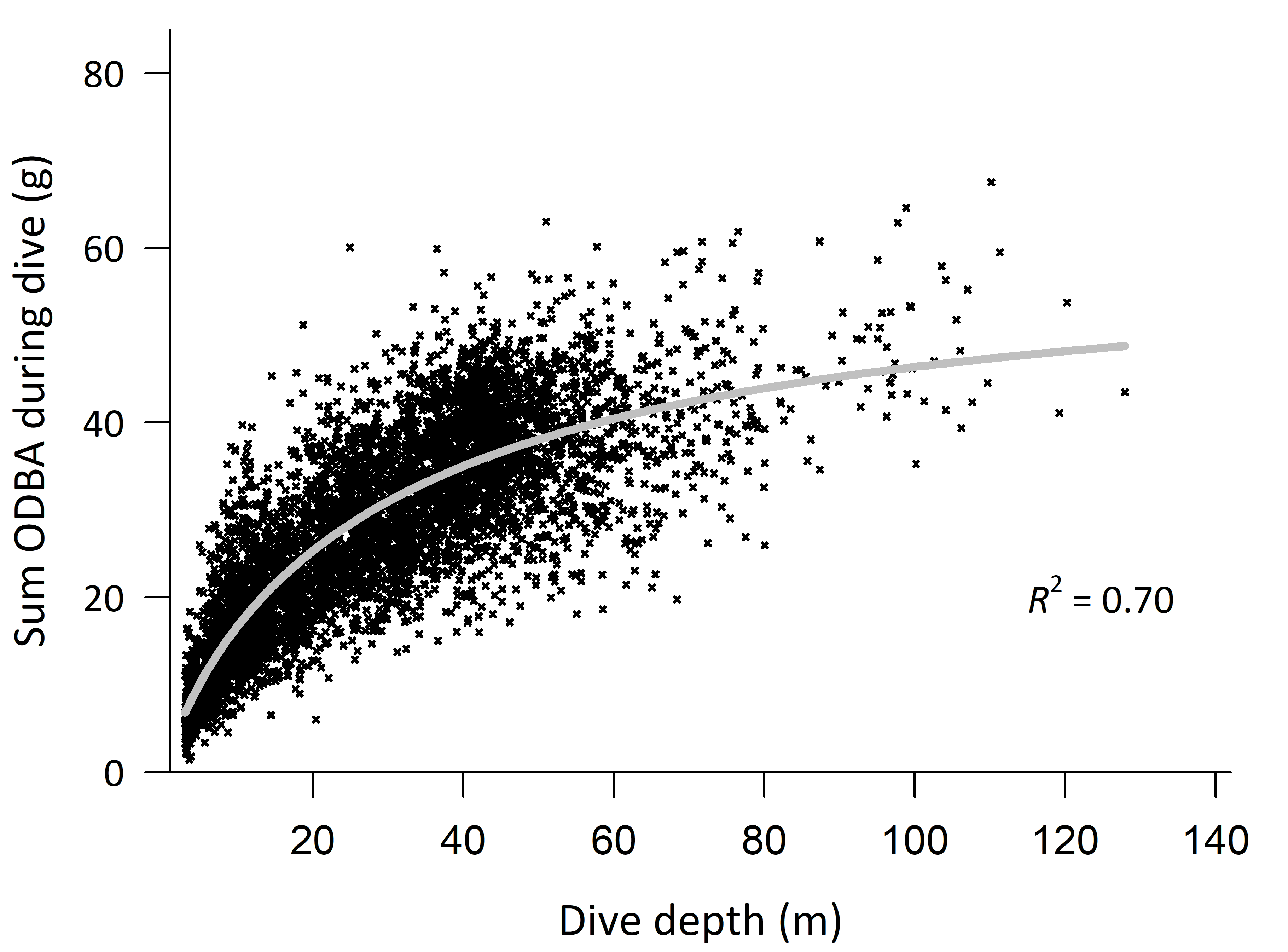


**Figure S10.** The relationship between the sum of Overall Dynamic Body Acceleration (ODBA) during dive and maximum dive depth for pelagic dives (based on the index of benthic diving behaviour, intra-depth zone; IDZ) carried out by gentoo penguin *Pygoscelis papua* breeding at Byers Peninsula, Livingston Island, Antarctica during chick guard (December 2016). Details for the regression curve are given in **Table S2**.


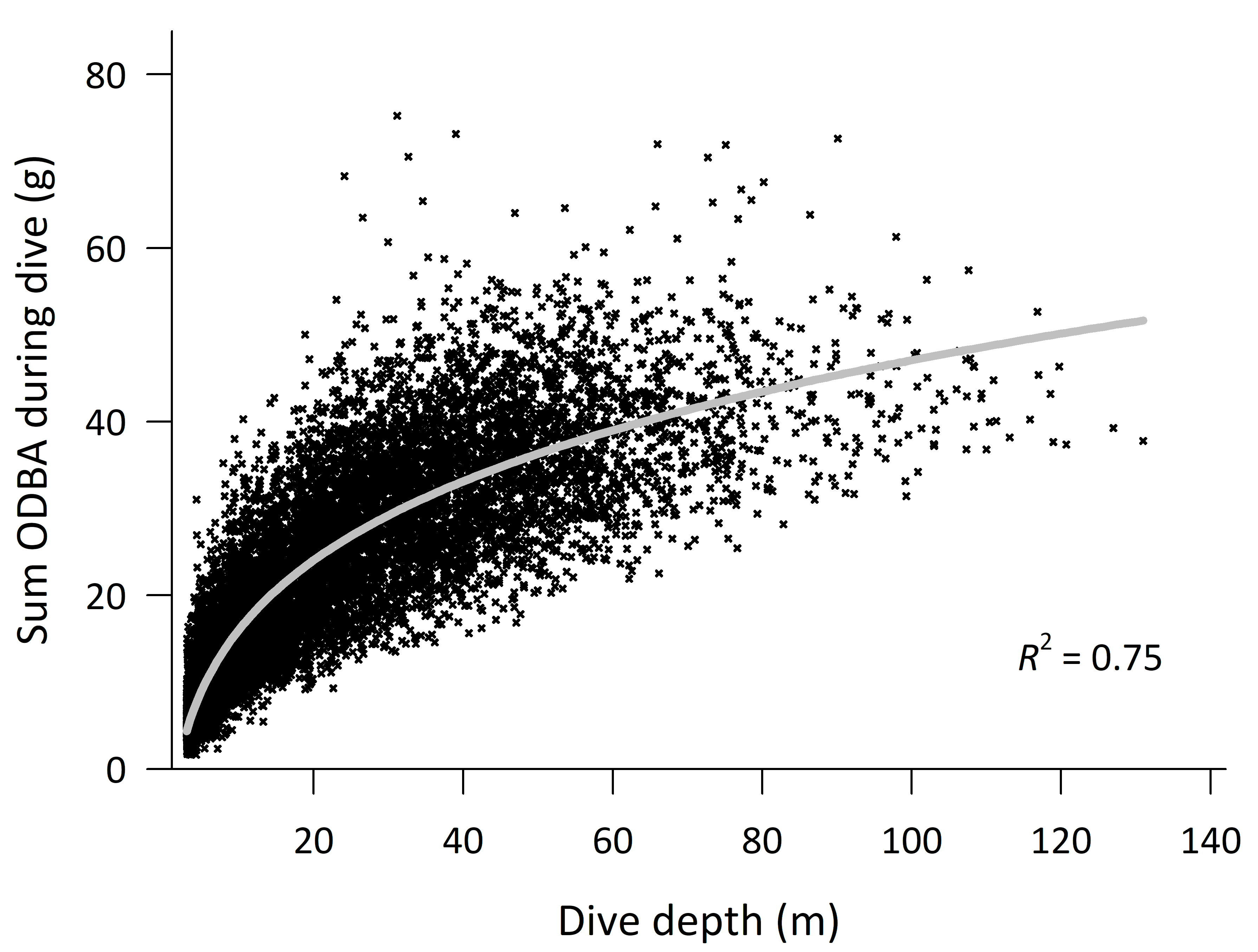


**Figure S11.** The relationship between the sum of Overall Dynamic Body Acceleration (ODBA) during dive and maximum dive depth for benthic dives (based on the index of benthic diving behaviour, intra-depth zone; IDZ) carried out by chinstrap penguins *Pygoscelis antarcticus* breeding at Vapour Col rookery, Deception Island, South Shetland Islands, Antarctica, during chick guard (January 2017). Details for the regression curve are given in **Table S2**.


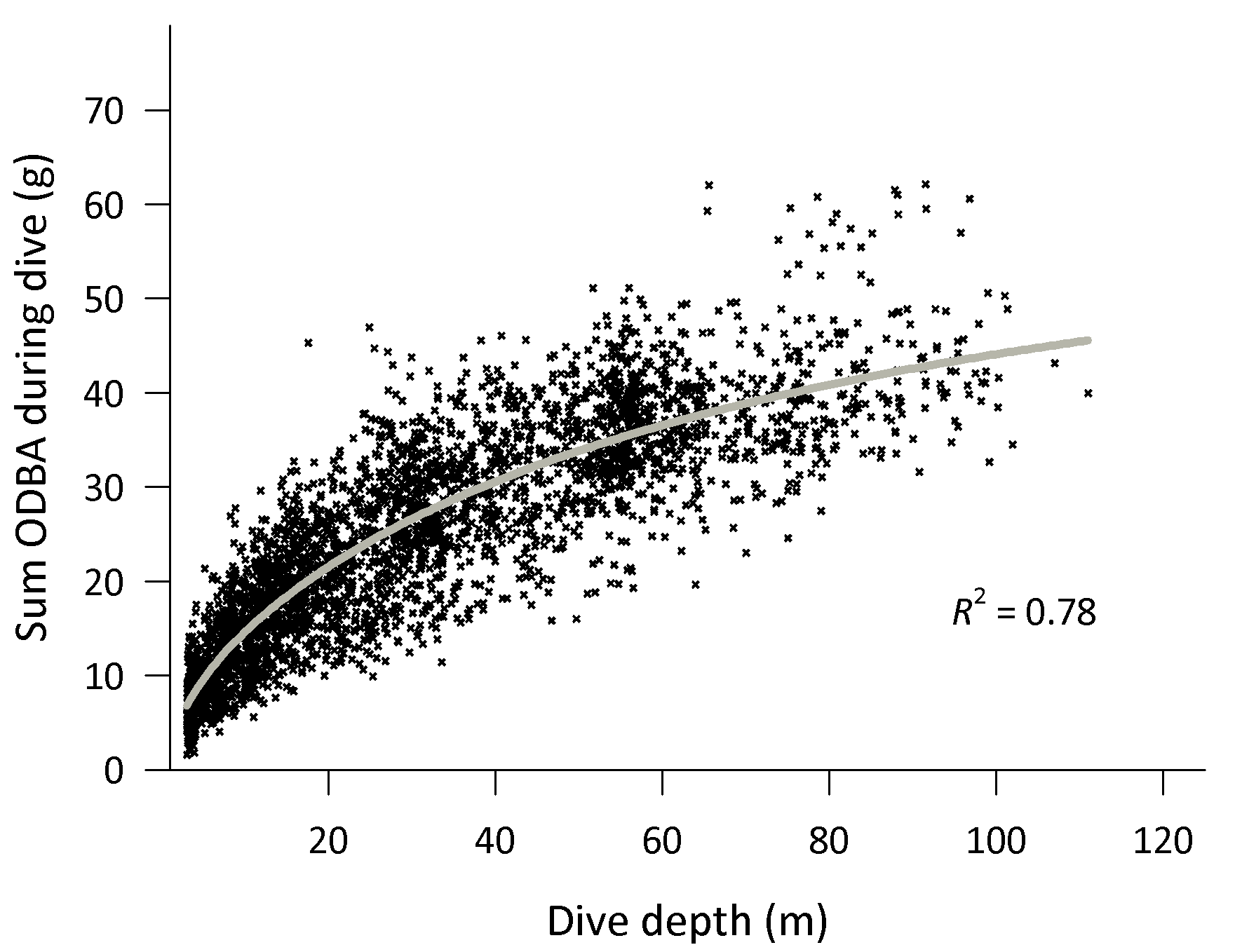


**Figure S12.** The relationship between the sum of Overall Dynamic Body Acceleration (ODBA) during dive and maximum dive depth for pelagic dives (based on the index of benthic diving behaviour, intra-depth zone; IDZ) carried out by chinstrap penguins *Pygoscelis antarcticus* breeding at Vapour Col rookery, Deception Island, South Shetland Islands, Antarctica, during chick guard (January 2017). Details for the regression curve are given in **Table S2**.


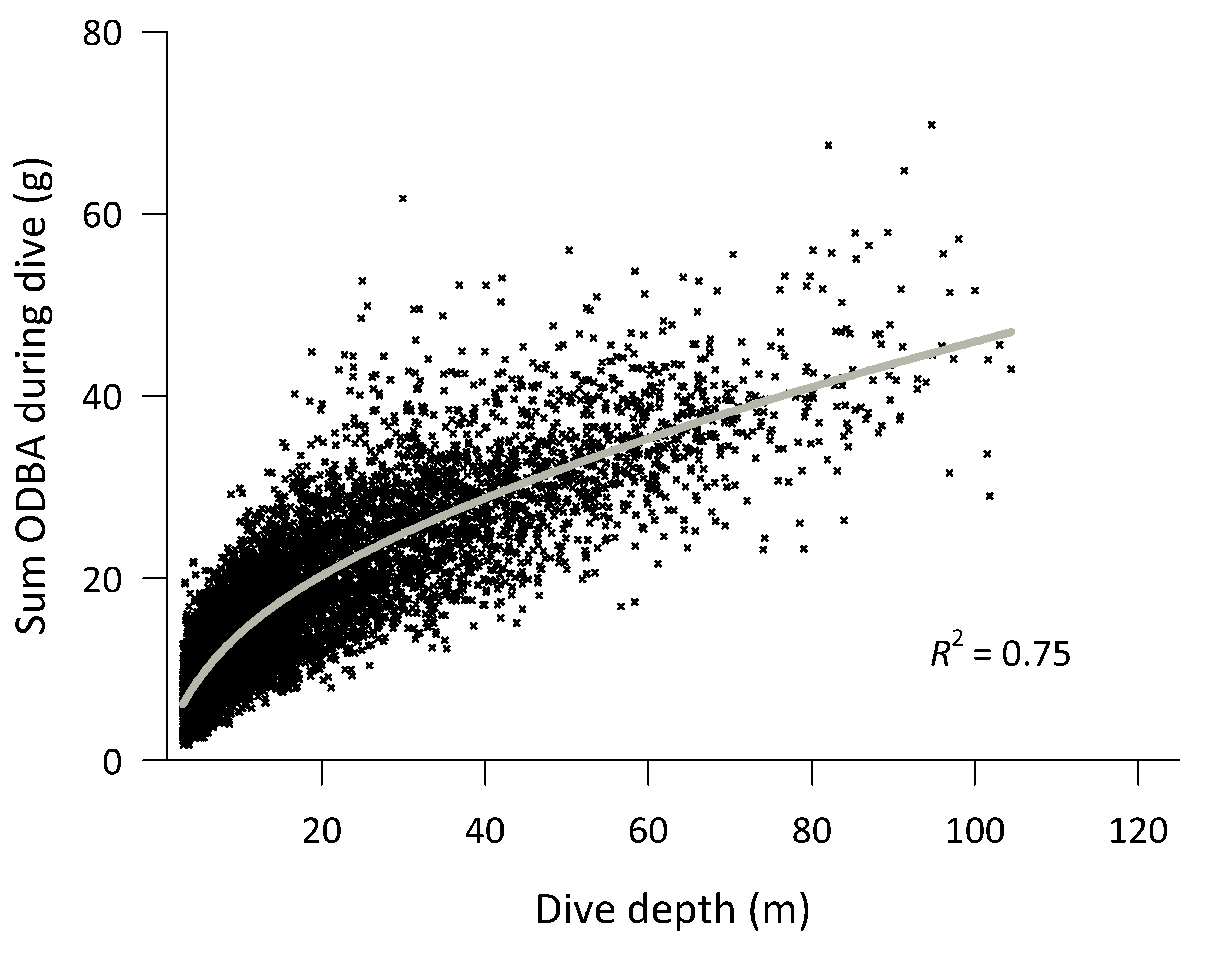


**Figure S13.** The relationship between the number of dives per trip and the maximum distance from the colony during a foraging trip carried out by gentoo penguin *Pygoscelis papua* breeding at Byers Peninsula, Livingston Island, Antarctica (chick guard; December 2016). Details for the regression curve are given in **Table S3**.





**Figure S14.** The relationship between the bottom time and the event maximum depth for benthic dives (based on the index of benthic diving behaviour, intra-depth zone; IDZ) carried out by gentoo penguin *Pygoscelis papua* breeding at Byers Peninsula, Livingston Island, Antarctica, during chick guard (December 2016). Details for the regression curve are given in **Table S4**.

**
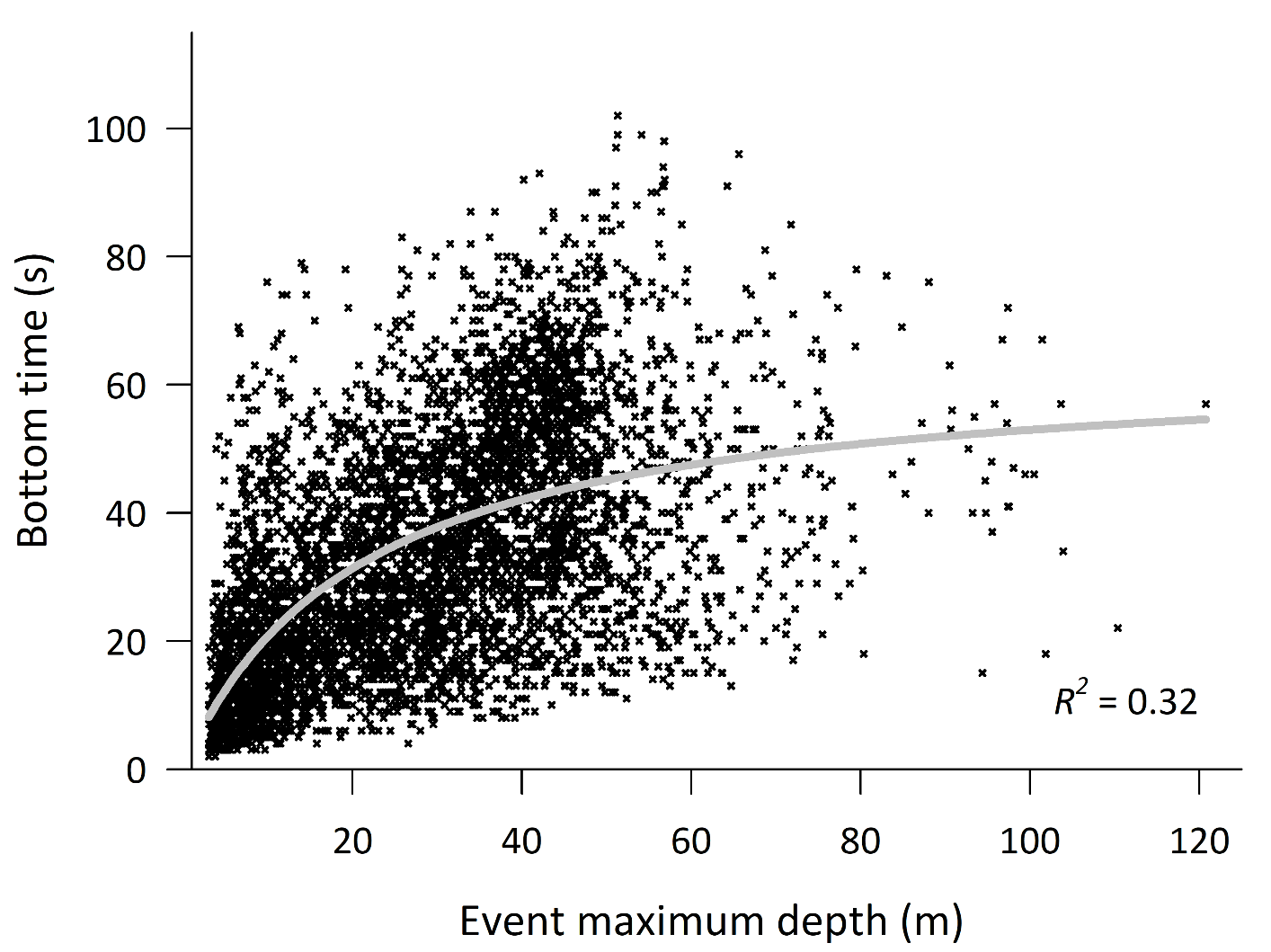
**

**Figure S15.** The relationship between the bottom time and the event maximum depth for pelagic dives (based on the index of benthic diving behaviour, intra-depth zone; IDZ) carried out by gentoo penguin *Pygoscelis papua* breeding at Byers Peninsula, Livingston Island, Antarctica, during chick guard (December 2016). Details for the regression curve are given in **Table S4**.


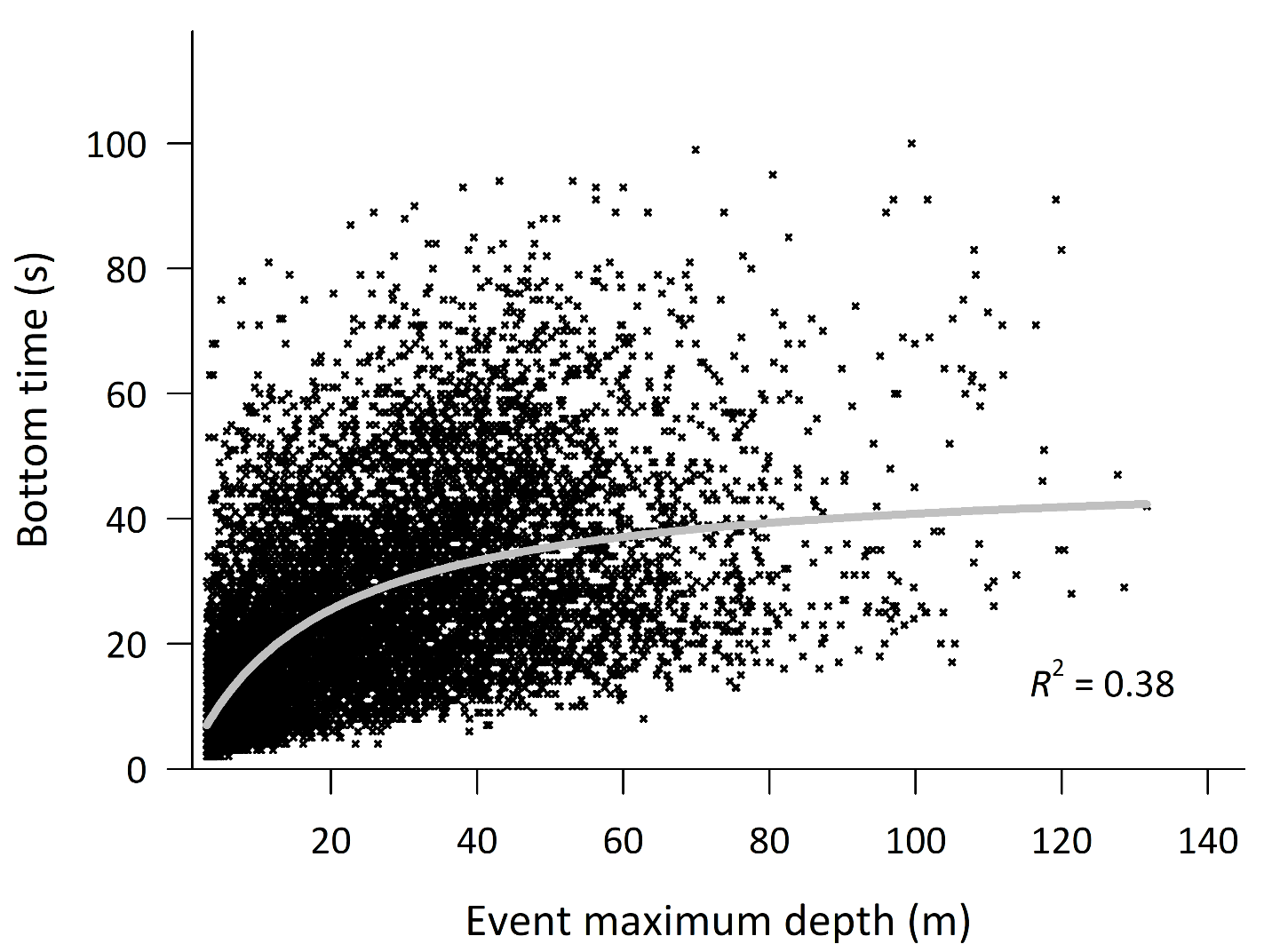


**Figure S16.** The relationship between the bottom time and the event maximum depth for benthic dives (based on the index of benthic diving behaviour, intra-depth zone; IDZ) carried out by chinstrap penguins *Pygoscelis antarcticus* breeding at Vapour Col rookery, Deception Island, South Shetland Islands, Antarctica, during chick guard (January 2017). Details for the regression curve are given in **Table S4**.


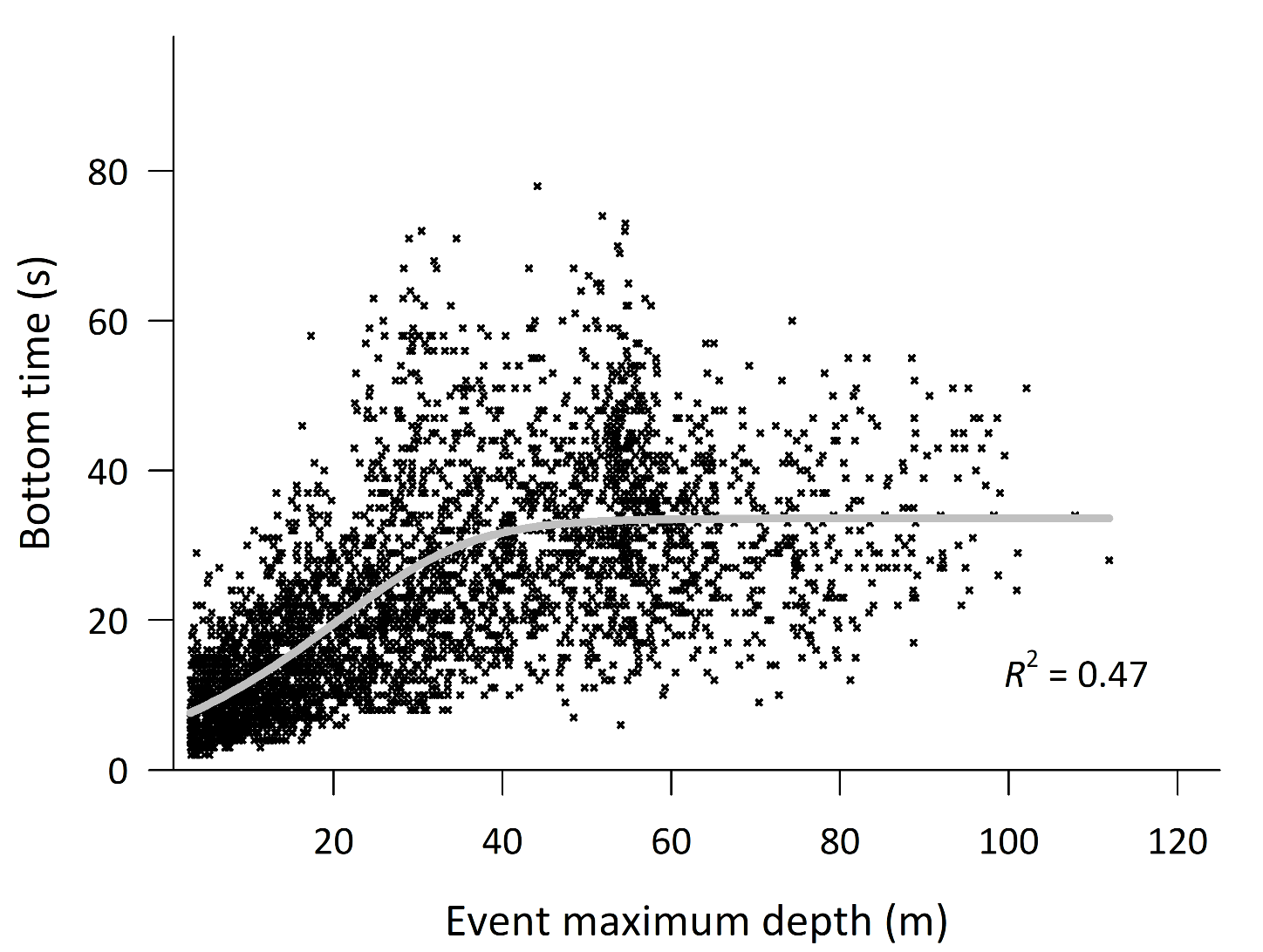


**Figure S17**. The relationship between the bottom time and the event maximum depth for pelagic dives (based on the index of benthic diving behaviour, intra-depth zone; IDZ) carried out by chinstrap penguins *Pygoscelis antarcticus* breeding at Vapour Col rookery, Deception Island, South Shetland Islands, Antarctica, during chick guard (January 2017). Details for the regression curve are given in **Table S4**.


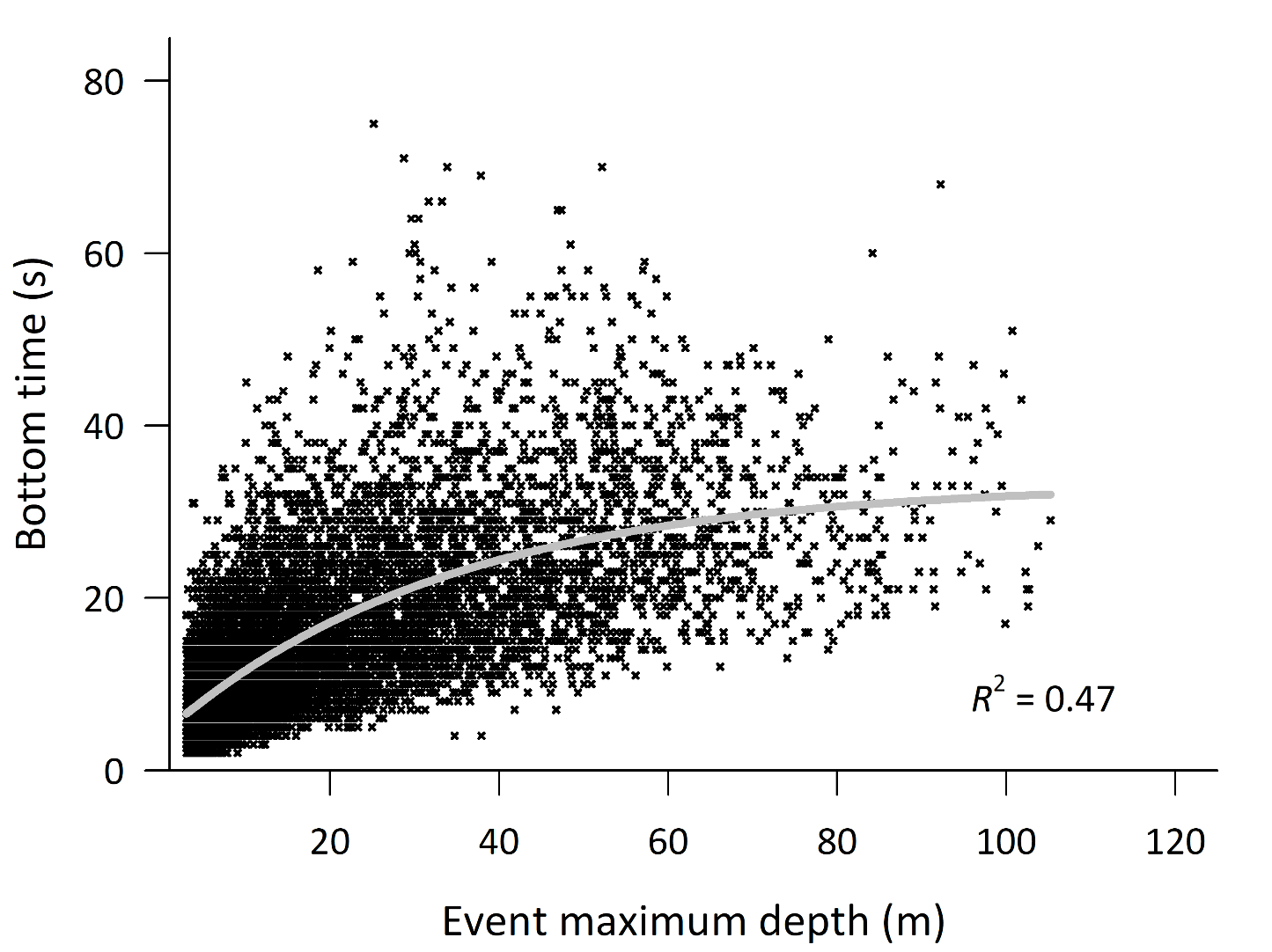


**Figure S18**. Location and size of other colonies of gentoo penguins *Pygoscelis papua* (degrees of red dots), chinstrap penguins *Pygoscelis antarcticus* (degrees of blue dots), and Antarctic Fur Seals *Arctocephalus gazella* (degrees of brown dots) in the South Shetland Islands, Antarctica. The locations and size of the Fur Seal colonies were obtained from Hucke-Gaete et al. (2004), while those from the penguin colonies were obtained from Naveen et al. (2000) and the Mapping Application for Penguin Populations and Projected Dynamics (MAPPPD) (Humphries et al. 2017) available at <http://www.penguinmap.com> . Size for penguins: pairs. Size for fur seals: individuals. Foraging trip coded as in Fig. 2A.


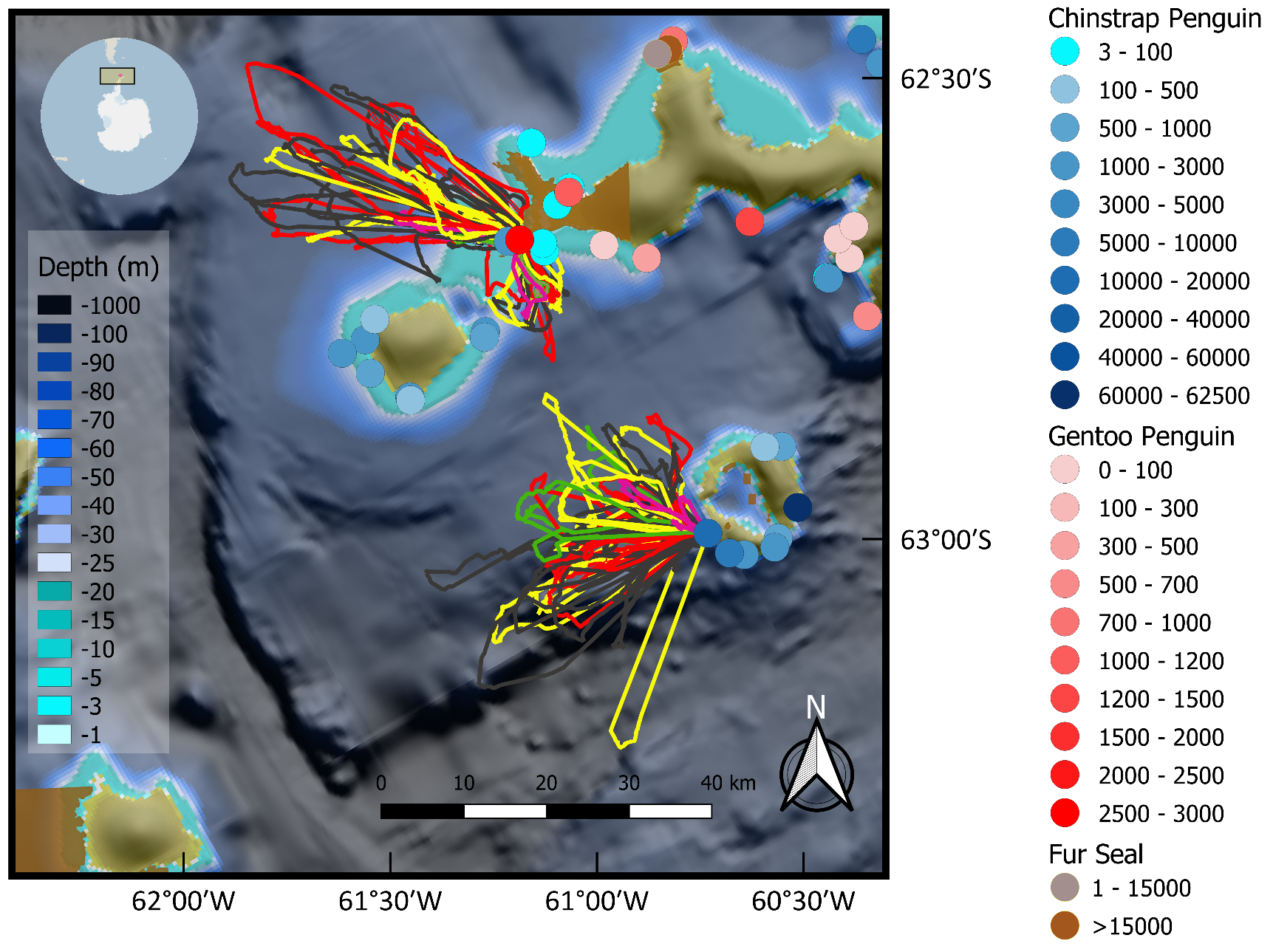


**Figure S19**. Kernel density distribution of dive locations and bathymetry. The 50% core areas are denoted by black lines, while 95% home ranges by yellow lines. Kernel density distributions represent the places where the penguins spent most of their forging time. Data from gentoo penguins *Pygoscelis papua* breeding at Devils Point, Byers Peninsula, Livingston Island, South Shetland Islands, Antarctica, during chick guard (December 2016), is further coded in short (dashed lines) and long trips (solid lines). Data from chinstrap penguins *Pygoscelis antarcticus* breeding at Vapour Col rookery, Deception Island, South Shetland Islands, Antarctica, during chick guard (January 2017) is denoted by solid lines only, as no distinction between short and long trips could be found. Depth (in m) is based on data from the International Bathymetric Chart of the Southern Ocean (IBCSO; Arndt et al. 2013).


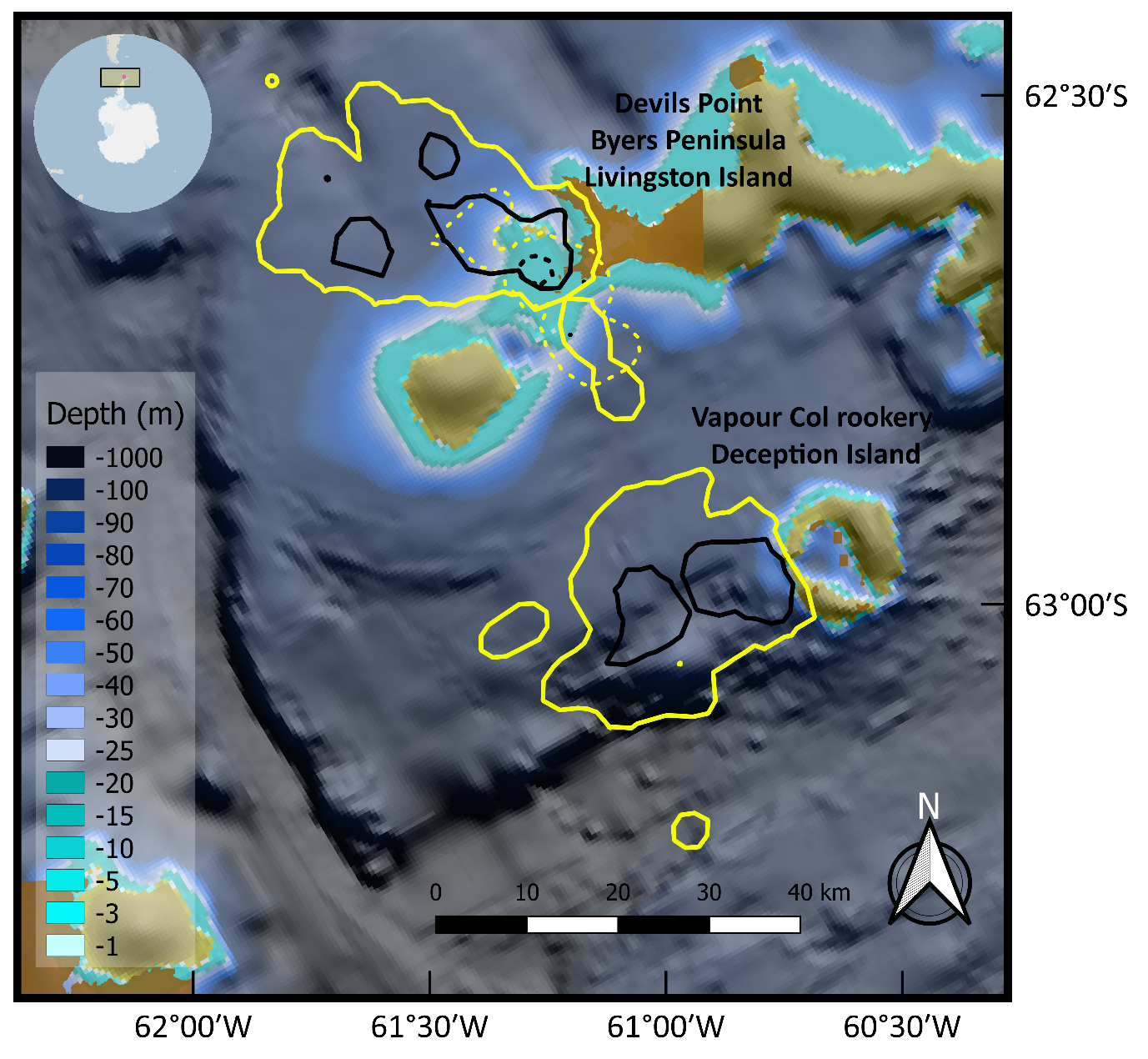


**Figure S20**. Energy landscapes based on the bathymetry around New Island, Falkland/Malvinas Islands, and the mass-specific total cost of foraging (diving plus commuting) by gentoo penguins *Pygoscelis papua* relative to the bottom time (in J kg^-1^ s^-1^), considering the different proportion of benthic and pelagic dives carried out by the penguins from the South End colony during the 2013 breeding season. For further details see Masello et al. (2017).


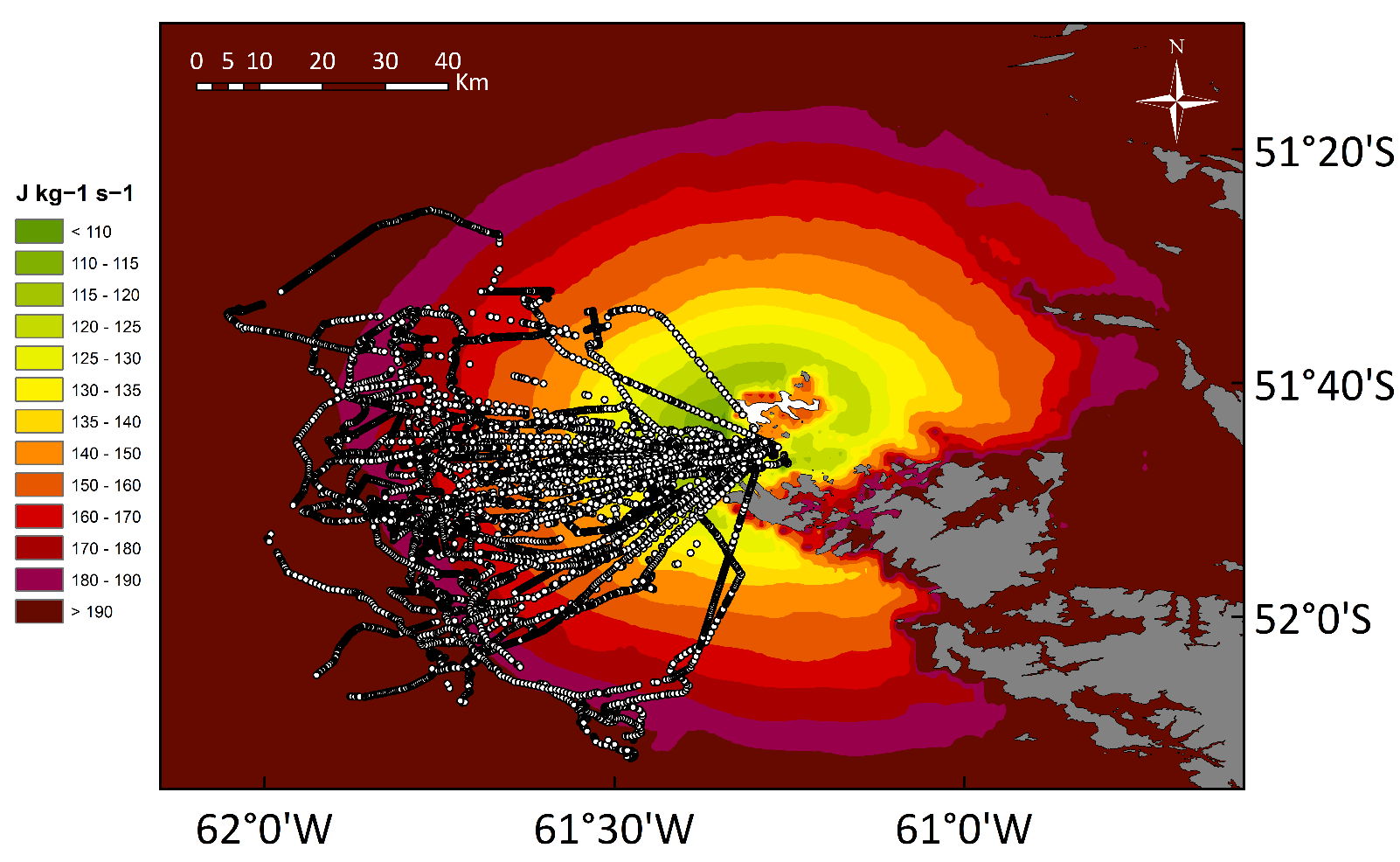


**Figure S21**. Energy landscapes based on the bathymetry around New Island, Falkland/Malvinas Islands, and the mass-specific total cost of foraging (diving plus commuting) by gentoo penguins *Pygoscelis papua* relative to the bottom time (in J kg^-1^ s^-1^), considering the different proportion of benthic and pelagic dives carried out by the penguins from the South End colony during the 2014 breeding season. For further details see Masello et al. (2017).

**
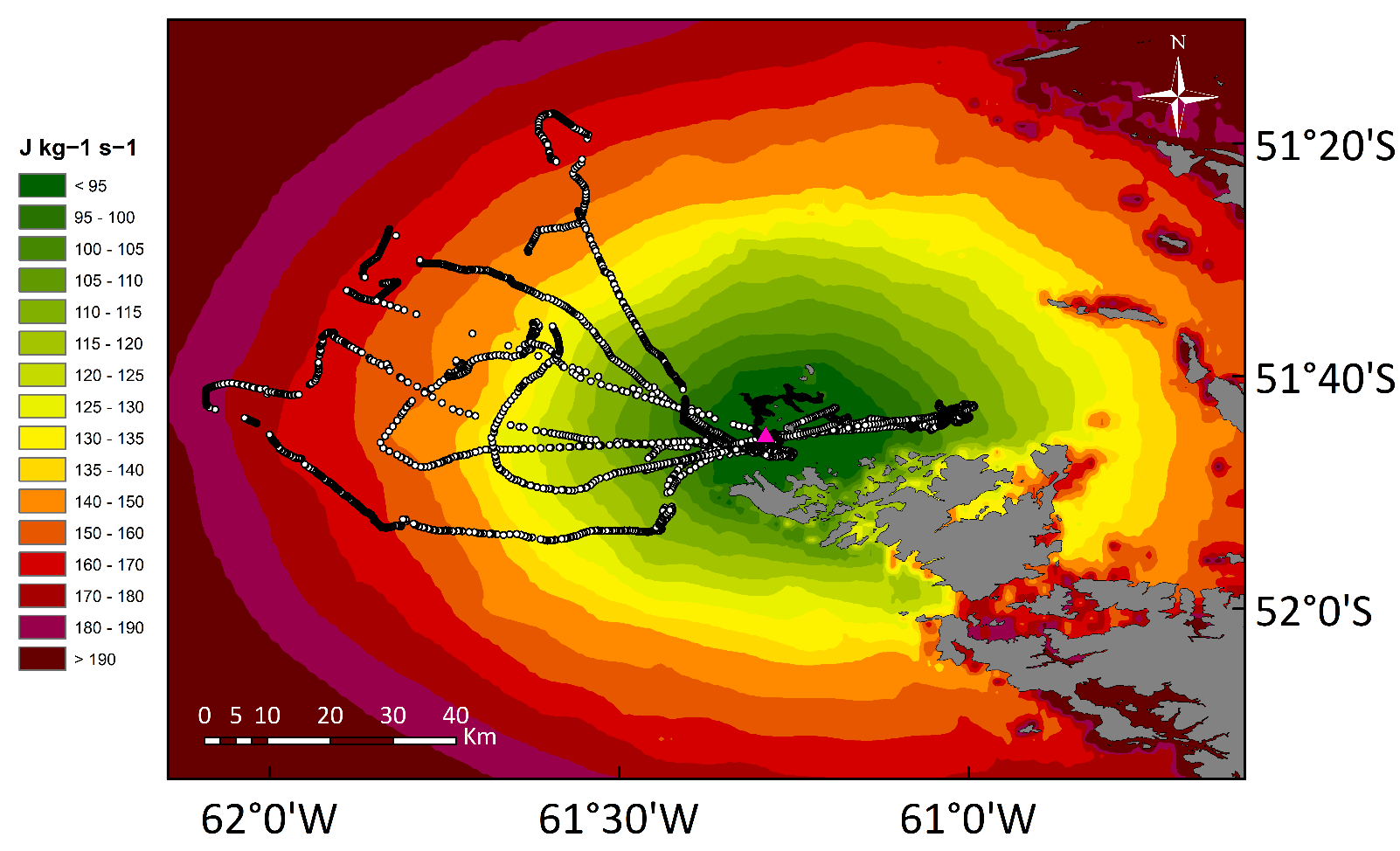
**

**Figure S22**. Energy landscapes based on the bathymetry around New Island, Falkland/Malvinas Islands, and the mass-specific total cost of foraging (diving plus commuting) by gentoo penguins *Pygoscelis papua* relative to the bottom time (in J kg^-1^ s^-1^), considering the different proportion of benthic and pelagic dives carried out by the penguins from the North End colony during the 2014 breeding season. For further details see Masello et al. (2017).


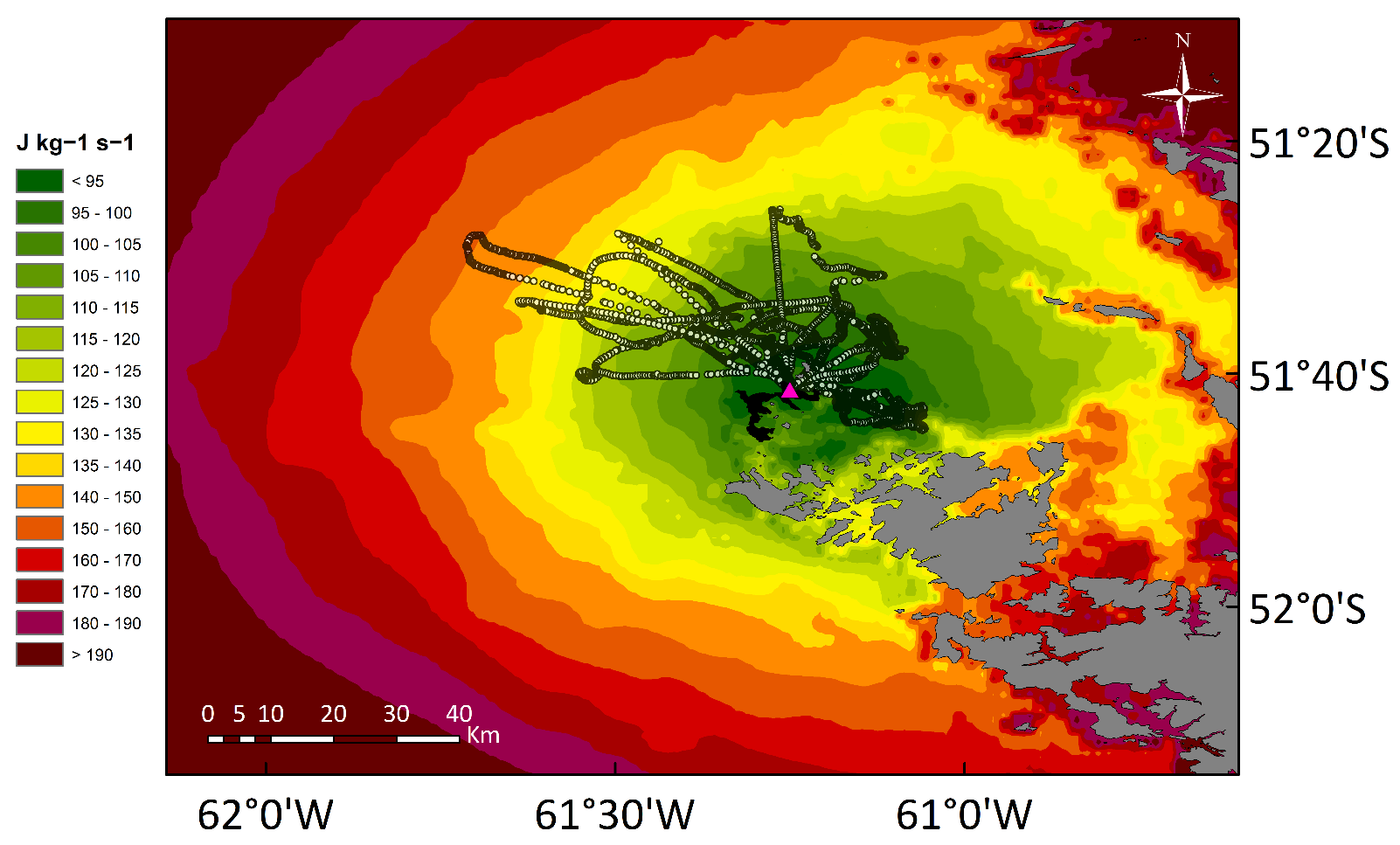


**Figure S23**. Diet composition using non-metric multidimensional scaling (NMDS) of molecular operational taxonomic units (MOTUs). Data corresponds to gentoo penguins *Pygoscelis papua* at Devils Point, Byers Peninsula, Livingston Island, South Shetland Islands, Antarctica, during chick guard (Dec 2016), and chinstrap penguins *Pygoscelis antarcticus* at Vapour Col rookery, Deception Island, South Shetland Islands, Antarctica, during chick guard (Jan 2017). A) includes the identity of the prey consumed, while B) the ellipses and C) the convex hulls connecting similar categories. Gentoo: black dots and lines, and dark grey shade. Chinstrap: royal blue dots and lines, and royal blue shade.


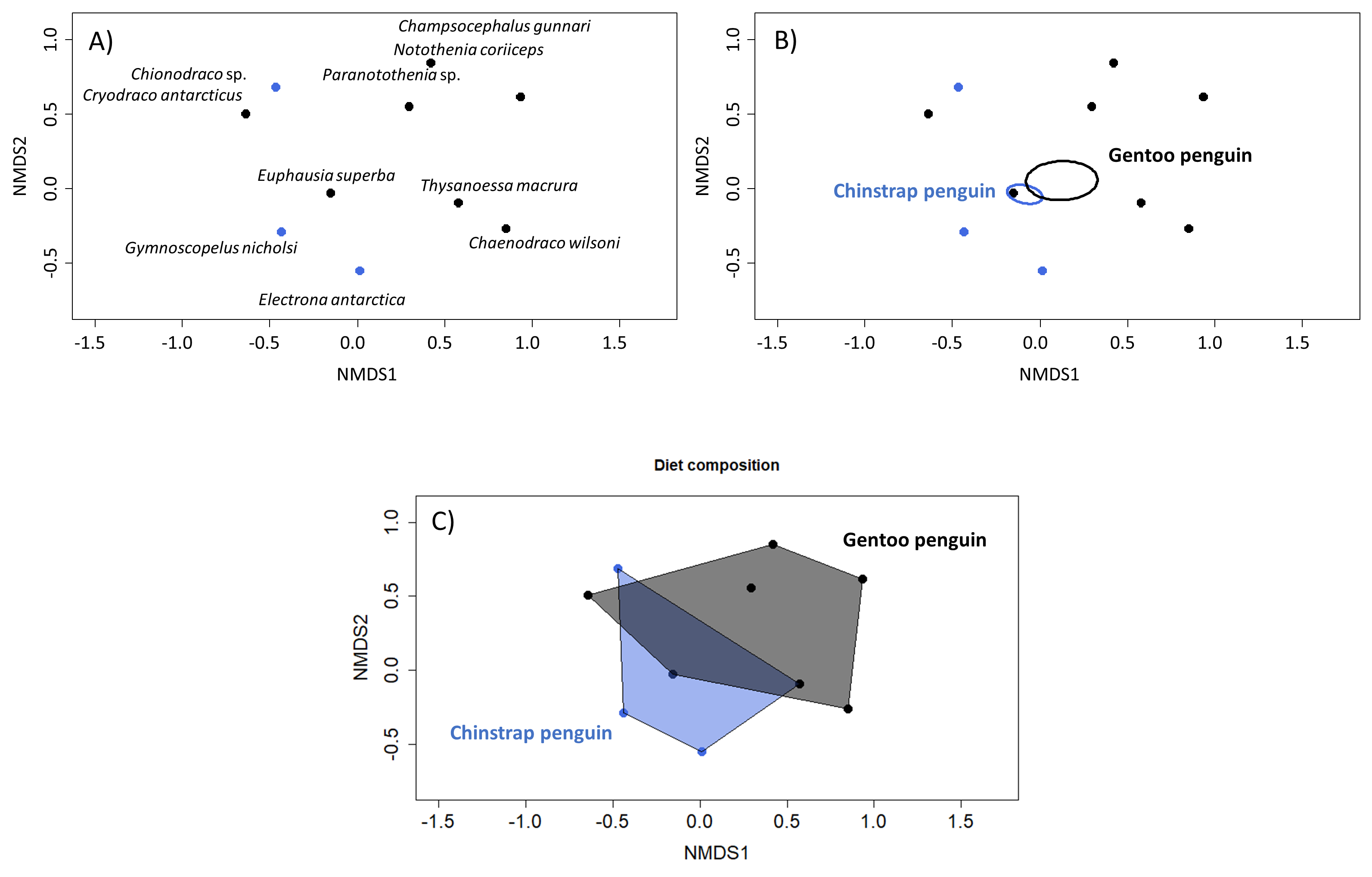


**Figure S24.** Diet composition using non-metric multidimensional scaling (NMDS) of molecular operational taxonomic units (MOTUs). Data corresponds to gentoo penguins *Pygoscelis papua* at Devils Point, Byers Peninsula, Livingston Island, South Shetland Islands, Antarctica, during chick guard (Dec 2016), and chinstrap penguins *Pygoscelis antarcticus* at Vapour Col rookery, Deception Island, South Shetland Islands, Antarctica, during chick guard (Jan 2017). A) includes the identity of the prey consumed, while B) the ellipses and C) the convex hulls connecting similar categories. The categories included are gentoo adults (back and dark grey), gentoo chicks (red), chinstrap adults (royal blue), and chinstrap chicks (cyan; not visible A) and C).


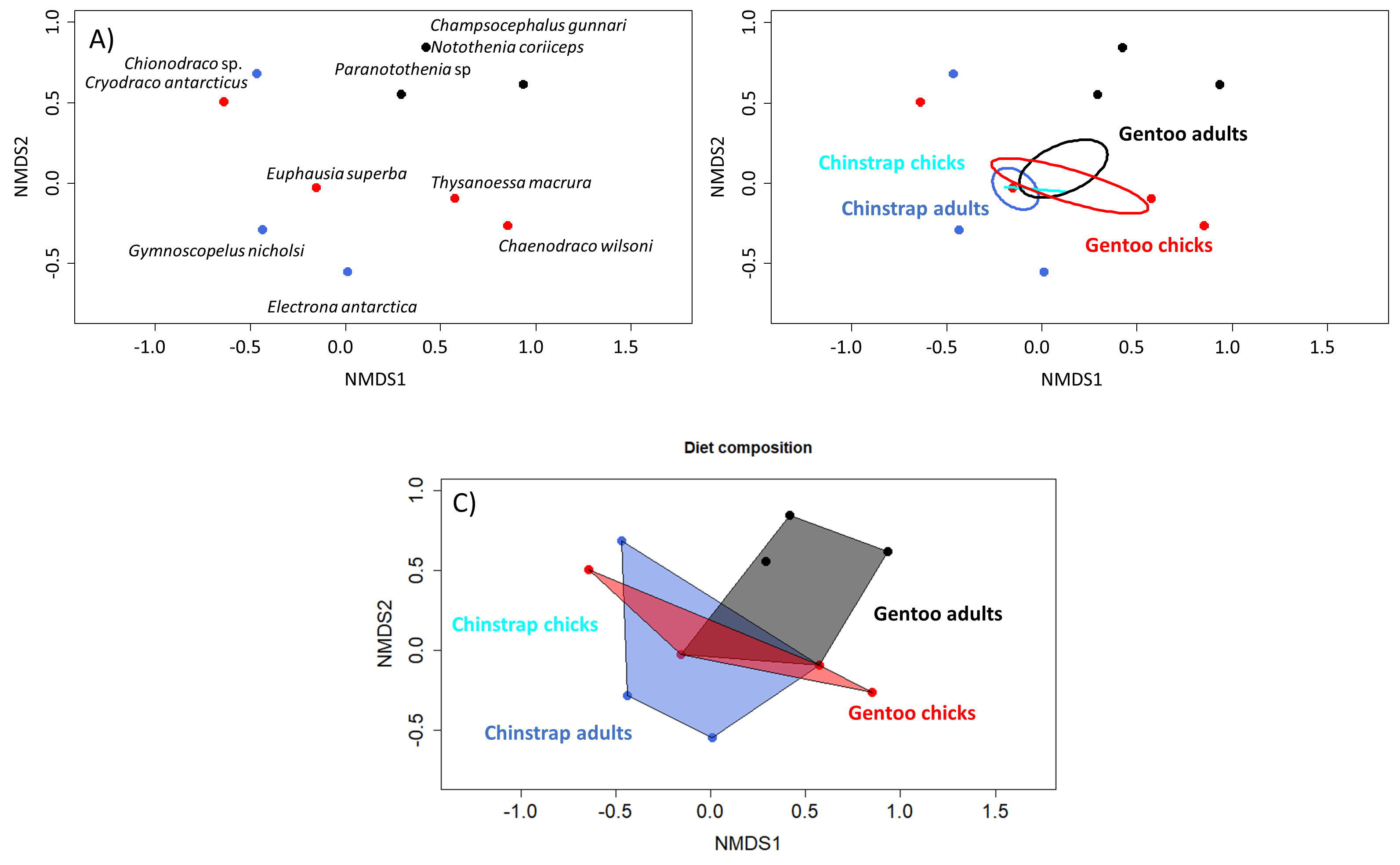


**Figure S25.** Diet composition using non-metric multidimensional scaling (NMDS) of molecular operational taxonomic units (MOTUs). Data corresponds to gentoo penguins *Pygoscelis papua* at Devils Point, Byers Peninsula, Livingston Island, South Shetland Islands, Antarctica, during chick guard (Dec 2016). A) includes the identity of the prey consumed, while B) the ellipses and C) the convex hulls connecting similar categories. The categories included are adults (black and dark grey) and chicks (red).


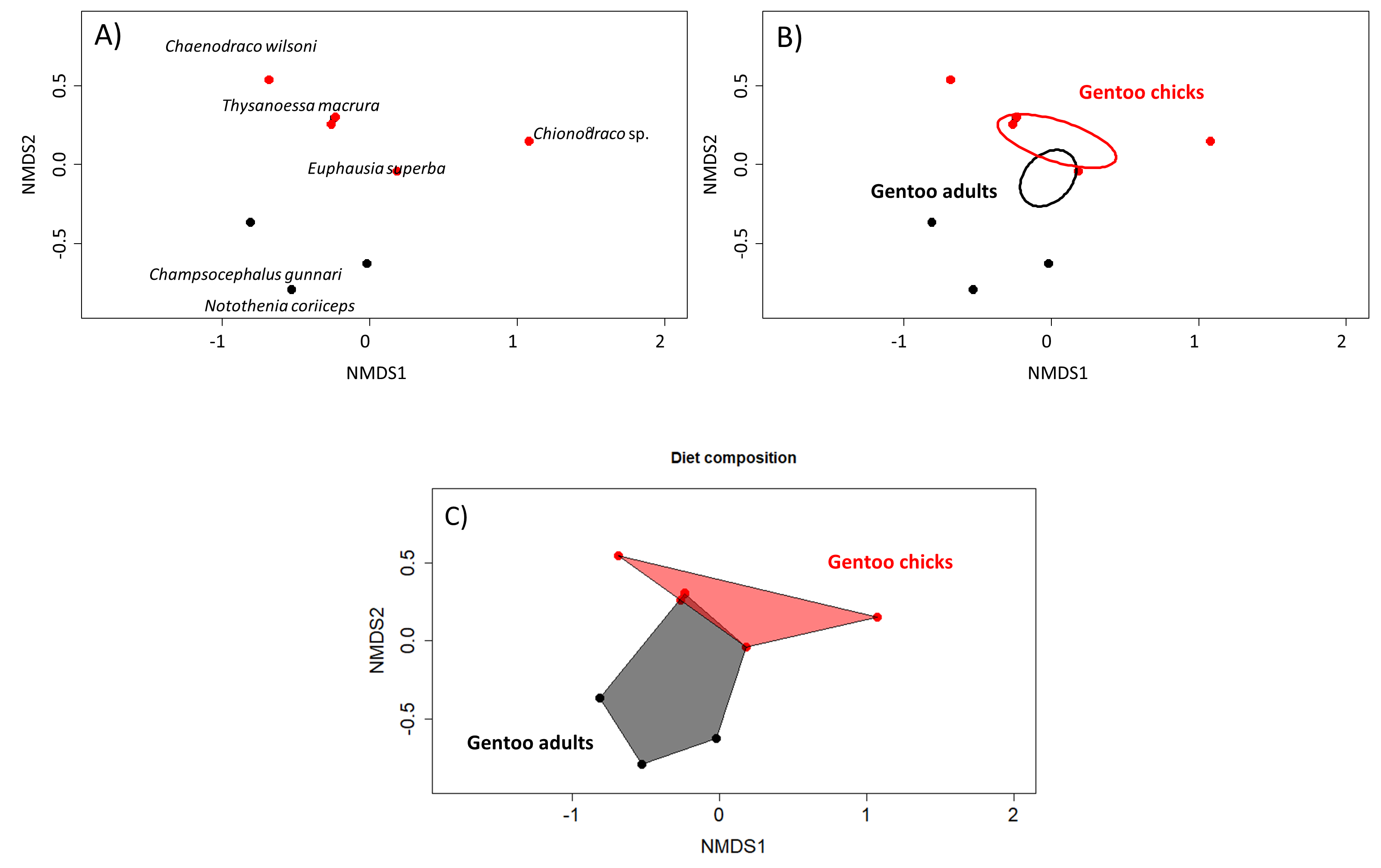


**Figure S26**. Diet composition using non-metric multidimensional scaling (NMDS) of molecular operational taxonomic units (MOTUs). Data corresponds to chinstrap penguins *Pygoscelis antarcticus* at Vapour Col rookery, Deception Island, South Shetland Islands, Antarctica, during chick guard (Jan 2017). A) includes the identity of the prey consumed, while B) the ellipses and C) the convex hulls connecting similar categories. The categories included are adults (royal blue) and chicks (cyan).


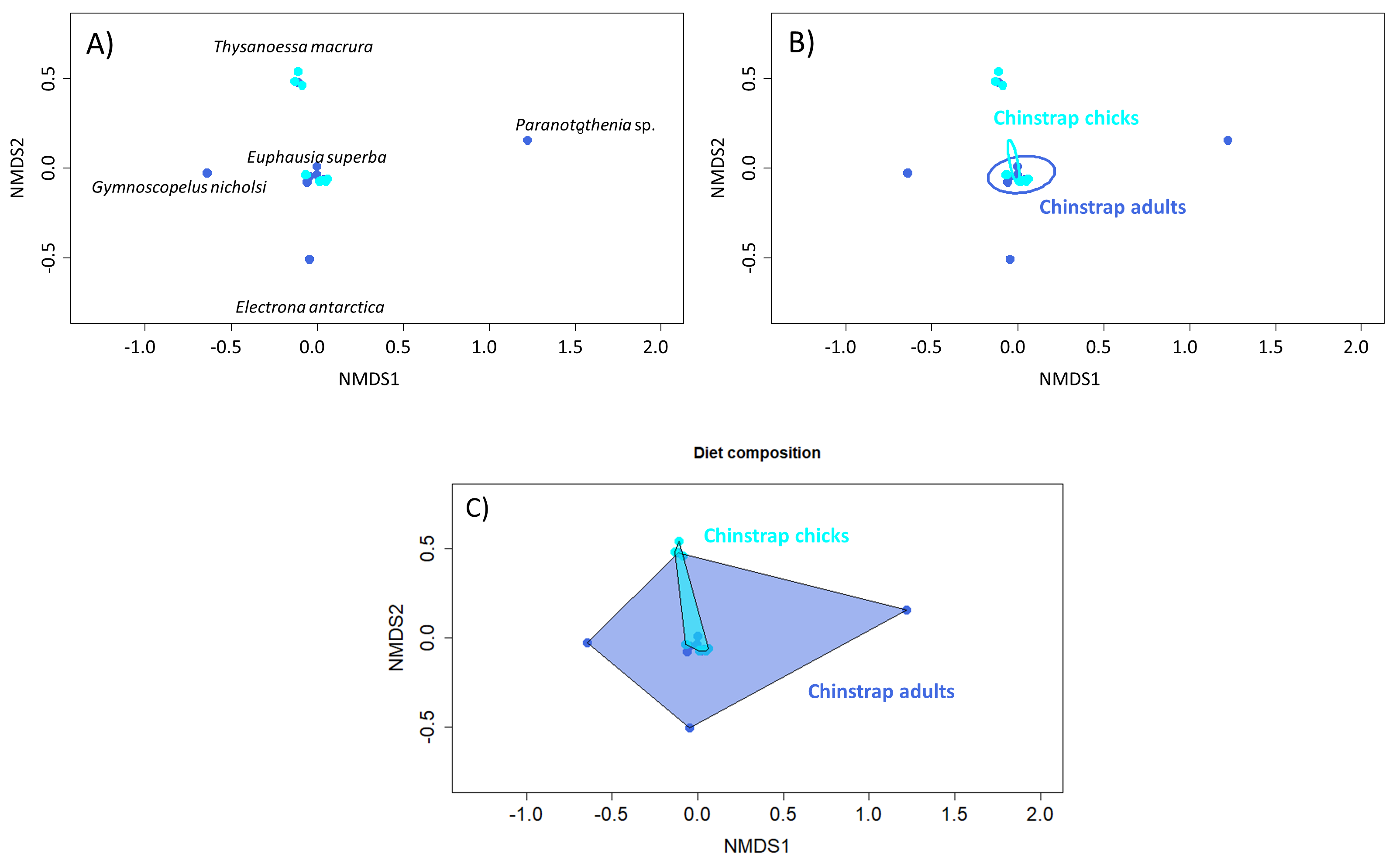


**Figure S27**. Boxplot of the ratio of two types of leucocytes, the heterophils and lymphocytes (H/L ratio), belonging to chinstrap penguins *Pygoscelis antarcticus* at Vapour Col rookery, Deception Island, South Shetland Islands, Antarctica, during chick guard (Jan 2017), and gentoo penguins *Pygoscelis papua* at Devils Point, Byers Peninsula, Livingston Island, South Shetland Islands, Antarctica, during chick guard (Dec 2016). Boxplots include medians, whiskers indicating variability outside the upper and lower quartiles, and outliers (circles).


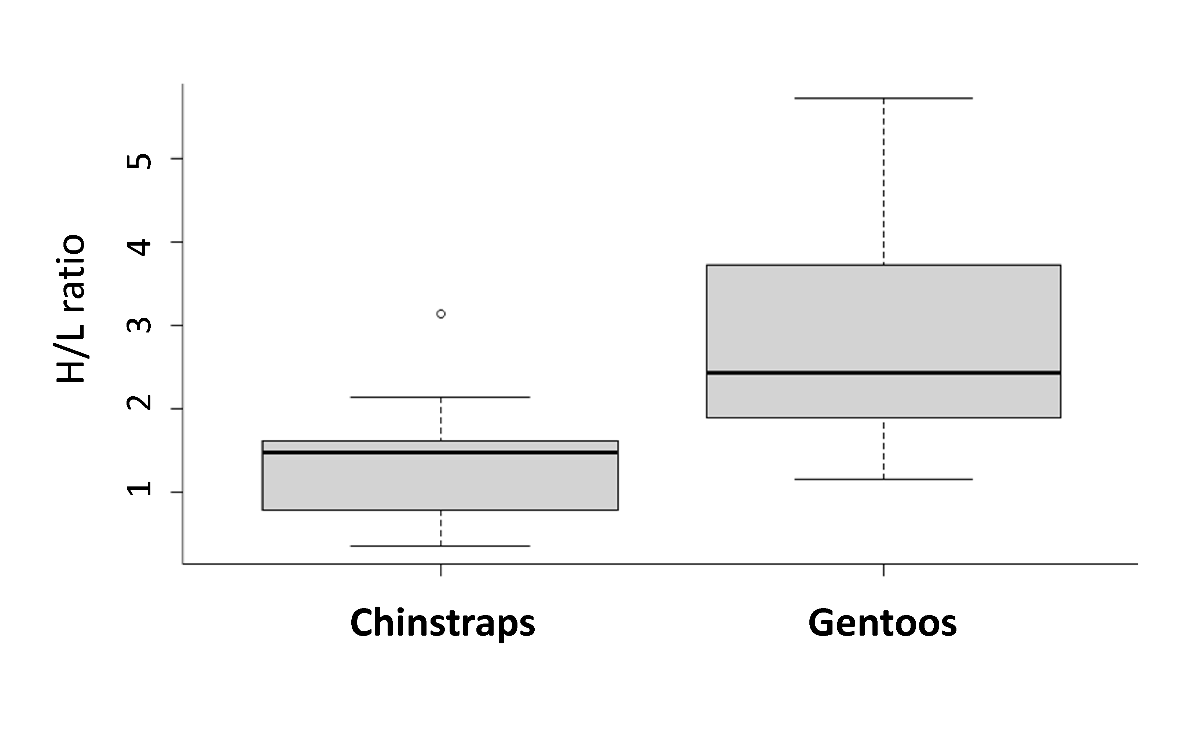


**Figure S28**. Dive depth of pelagic dives corresponding to gentoo penguins *Pygoscelis papua* breeding at New Island (Falkland/Malvinas Islands), during chick guard (December) in 2013 and 2014, gentoo penguins breeding at Devils Point, Byers Peninsula, Livingston Island, South Shetland Islands, Antarctica, during chick guard (December 2016), and chinstrap penguins *Pygoscelis antarcticus* breeding at Vapour Col rookery, Deception Island, South Shetland Islands, Antarctica, during chick guard (January 2017).





**Figure S29**. Standardised abundance of the Antarctic Krill *Euphausia superba* obtained from the KRILLBASE (Atkinson et al. 2017) for the sector between 60−65°S and 55−65°W (A), and Antarctic Krill catches for the CCAMLR Area 48 (B).





**Additional Methods**

*Molecular analysis of the diet*

In the case of the penguins from Antarctica, we extracted deoxyribonucleic acid (DNA) from the faecal samples using the QIAamp DNA Stool Mini Kit (Qiagen) following the protocol provided with the kit. Considering previously published information on the diet of South Shetland Islands penguins, we used primers targeting Euphausiidae, Osteichthyes and Metazoa (Additional file 1, Table S8; Volkman et al. 1980, Jabłoński 1985, Panasiuk et al. 2020). We performed polymerase chain reaction (PCR) amplifications in single reactions using Multiplex PCR Kits (Qiagen). In this case, we prepared next generation sequencing (NGS) libraries at the facilities of the Justus Liebig University Giessen (Germany) using the NEBNext Ultra DNA Library Prep Kit for Illumina (New England Biolabs, Ipswich, MA). The PCR product was pyrosequenced at SeqIT (Kaiserslautern, Germany). Samples from Antarctica were used individually in library preparations and thus, we obtained information at the individual level. Index PCRs were performed to individually mark PCR products with specific indices (P5/P7 combinations) added to the sequencing adapters. The 30 µl index PCR reactions for the Multiplex protocol consisted of 7.5 μl Multiplex PCR Mastermix, 2.1 μl of forward primer (10 µM) and 2.1 μl of reverse primer (10 µM), 3 μl template, and 15.3 μl PCR grade water. Thermal cycling conditions were 91°C for 1 min, followed by 7 cycles of: 95°C for 10 s, 60°C for 30 s, and 72°C for 30 s, and a final extension at 72°C for 5 min.

We processed the samples from New Island at the facilities of the Cardiff School of Biosciences, Cardiff University and at the Biomolecular Analysis Facility-Sheffield, Natural Environment Research Council (U.K.). We extracted DNA from the New Island samples, using the QIAamp DNA Stool Mini Kit (Qiagen), following Zeale et al. (2011). Samples corresponded to both adults and chicks (Additional file 1, Table S6). To test for DNA extractions success, we amplified all the extractions, as well as positive and negative controls, with Metazoa general primers (Leray et al. 2013). We used pyrosequencing libraries to comprehensively target all the major potential prey species, preparing the libraries with NEBNext Ultra DNA Library Prep Kit for Illumina (New England Biolabs, Ipswich, MA). To ensure good coverage and resolution of the potential prey consumed by the penguins, we selected five primer sets (Additional file 1, Table S8; Masello et al. 2010, 2017, Handley et al. 2016). We prepared four pyrosequencing libraries using primers targeting Osteichthyes, Cephalopoda, Amphipoda and Metazoa, all of which were previously used for marine predators such as fish (Leray et al. 2013) or seabirds (Deagle et al. 2007, Horswill et al. 2018) and amplify target prey sequences between 180 and 375 base pairs (Additional file 1, Table S8). We also tested Euphausidae primers in a subset of New Island samples (n = 79). However, we established the presence of Euphausidae with certainty only in less than 2% of the samples and hence, we did not include an Euphausidae library for pyrosequencing. Due to initial funding restrictions, we pooled the samples from New Island into seven different groups for Metazoa and Osteichthyes primers comprising a combination of colony (South End, SE, and North End, NE), breeding season (2013−4, 2014−5) and age group (adult, first hatched chick, second hatched chick, unknown): 2013−4 SE adults, 2013−4 SE first hatched chick, 2013−4 SE second hatched chick, 2013−4 SE unknown, 2014−5 SE all, and 2014−5 NE all. We did not split the samples from 2014 by age group due to small sample sizes. We created five pools for Cephalopoda and Amphipoda primers to separate the different colonies and years but ignored differences between age groups due to small sample sizes since fewer individuals would have consumed these prey types. For each primer library, we pooled samples in approximately equimolar proportions with reference to the brightness of bands on a 1.5% agarose gel stained with SYBR®Safe (Thermo Fisher Scientifc, Paisley, UK) when compared to a standardized 100‐bp ladder. Then, we purified the pooled samples using a QIAquick PCR Purification kit. We quantified each pool of samples using the different primers with Qubit and verified quality control using the TAPE/BioAnalyzer to confirm purification success, peak size and DNA concentration of each library; we combined Metazoa and Osteichthyes primer pools into one pool and Amphipoda and Cephalopoda into a second pool based on band sizes. We combined the samples considering the number of individual birds pooled into each one and the respective concentration. As library controls and to obtain information about any potential contamination, we included PCR negative controls. New Island PCR products were sequenced on Illumina (Illumina, Inc.) at the Biomolecular Analysis Facility-Sheffield, Natural Environment Research Council (U.K.).

**Additional References**

Arndt JE, Schenke HW, Jakobsson M, Nitsche FO, Buys G, Goleb yB, Rebesco M, Bohoyo F, Hon gJ, Black J, Greku R, Udintsev G, Barrios F, Reynoso‐Peralta W, Taisei M, Wigley R. 2013. The International Bathymetric Chart of the Southern Ocean (IBCSO) Version 1.0—A new bathymetric compilation covering circum‐Antarctic waters. *Geophysical Research Letters* 40: 3111-3117.

Atkinson A, Hill SL, Pakhomov EA, Siegel V, Anadon R, Sanae C, Daly KL, Downie R, Fielding S, Fretwell P, Gerrish L, Hosie G, Jessopp MJ, So K, Krafft BA, Loeb V, Nishikawa J, Peat HJ, Reiss CS, Ross RM, Quetin LB, Schmidt K, Steinberg DK, Subramaniam RC, Tarling GA, Ward P. 2017. KRILLBASE: a circumpolar database of Antarctic krill and salp numerical densities, 1926-2016. *Earth System Science Data* 9: 193-210.

Deagle BE, Tollit DJ, Jarman SN, Hindell MA, Trites AW, Gales NJ. 2005. Molecular scatology as a tool to study diet: analysis of prey DNA in scats from captive Steller sea lions. *Molecular Ecology* 14: 1831-1842.

Deagle BE, Gales NJ, Evans K, Jarman SN, Robinson S, Trebilco R, Hindell MA. 2007. Studying Seabird Diet through Genetic Analysis of Faeces: A Case Study on Macaroni Penguins (*Eudyptes chrysolophus*). *PLoS One* 2: e831.

Handley JM, Baylis AM, Brickle P, Pistorius P. 2016. Temporal variation in the diet of gentoo penguins at the Falkland Islands. *Polar Biology* 39: 283–296.

Horswill C, Jackson JA, Medeiros R, Nowell RW, Trathan PN, O'Connell TC. 2018. Minimising the limitations of using dietary analysis to assess foodweb changes by combining multiple techniques. *Ecological Indicators* 94: 218-225.

Humphries GRW, Naveen R, Schwaller M, Che-Castaldo C, McDowall P, Schrimpf M, Lynch HJ. 2017. Mapping Application for Penguin Populations and Projected Dynamics (MAPPPD): data and tools for dynamic management and decision support. *Polar Record* 53: 160-166.

Jabłoński PG. 1985 The diet of penguins on King George Island, South Shetland Islands. *Acta Zoologica Cracoviensia* 29: 117-186.

Jarman SN, Redd KS, Gales NJ. 2006. Group-specific primers for amplifying DNA sequences that identify Amphipoda, Cephalopoda, Echinodermata, Gastropoda, Isopoda, Ostracoda and Thoracica. *Molecular Ecology Notes* 6: 268-271.

Leray M, Yang JY, Meyer CP, Mills SC, Agudelo N, Ranwez V, Boehm JT, Machida RJ. 2013. A new versatile primer set targeting a short fragment of the mitochondrial COI region for metabarcoding metazoan diversity: application for characterizing coral reef fish gut contents. *Frontiers in Zoology* 10: 1-14.

Masello JF, Mundry R, Poisbleau M, Demongin L, Voigt CC, Wikelski M, Quillfeldt P. 2010. Diving seabirds share foraging space and time within and among species. *Ecosphere* 1: art19.

Masello JF, Kato A, Sommerfeld J, Mattern T, Quillfeldt P. 2017. How animals distribute themselves in space: variable energy landscapes. *Frontiers in Zoology* 14: e33.

Medeiros-Mirra RJ. 2010. The Migration Strategy, Diet & Foraging Ecology of a Small Seabird in a Changing Environment. PhD Thesis: Cardiff University.

Naveen R, Forrest S, Dagit R, Blight L, Trivelpiece W, Trivelpiece S. 2000. Censuses of penguin, blue-eyed shag, and southern giant petrel populations in the Antarctic Peninsula region, 1994–2000. *Polar Record* 36: 323-334.

Panasiuk A, Wawrzynek-Borejko J, Musiał A, Korczak-Abshire M. 2020. Pygoscelis penguin diets on King George Island, South Shetland Islands, with a special focus on the krill Euphausia superba. *Antarctic Science* 32: 21-28.

Volkman NJ, Presler P, Trivelpiece W. 1980. Diets of Pygoscelid Penguins at King George Island, Antarctica. *Condor* 82: 373-378.

Zeale MRK, Butlin RK, Barker GLA, Lees DC, Jones G. 2011. Taxon-specific PCR for DNA barcoding arthropod prey in bat faeces. *Molecular Ecology Resources* 11: 236-244.
